# Systemic targeting of aberrant neovascular tufts using trehalose-dendrimer nanocarriers for the treatment of proliferative retinopathies

**DOI:** 10.7150/thno.120357

**Published:** 2026-01-01

**Authors:** Anu Rani, Shivantika Bisen, Rishi Sharma, Aqib Iqbal Dar, Anamika Sharma, Anubhav Dhull, Nina Palmer, Kenneth John Goody, Nikhlesh K Singh, Anjali Sharma

**Affiliations:** 1 Department of Chemistry, College of Arts and Sciences, Washington State University, 1470 NE College Ave, Pullman, WA, 99164, USA.; 2 Integrative Biosciences Center, Wayne State University, Detroit, MI, 48202, USA.; 3 Department of Ophthalmology, Visual and Anatomical Sciences, School of Medicine, Wayne State University, Detroit, MI, 48202, USA.

**Keywords:** dendrimer, targeted drug delivery, retinal neovascularization, neovascular tufts targeting, proliferative retinopathies

## Abstract

**Rationale:** Proliferative retinopathies are the leading causes of blindness worldwide. Current treatment paradigms rely heavily on intravitreal injections of anti-vascular endothelial growth factor A (anti-VEGFA) agents, which, despite their efficacy, are associated with ocular complications and patient discomfort. To address these challenges, we have developed a novel mixed-layered trehalose-functionalized dendrimer (Tre-D) conjugated with Axitinib, a multi-receptor tyrosine kinase inhibitor, (Tre-D-Axitinib) for systemic delivery to aberrant neovascular tufts in retina.

**Methods:** Tre-D is synthesized through a scalable and convenient synthetic methodology using click chemistry. The *in vitro* cytocompatibility, uptake and angiogenesis assays are carried out in Human Retinal Microvascular Endothelial Cells (HRMECs), Human umbilical vein endothelial cells (HUVECs) and macrophages (RAW-Blue). The *in vivo* uptake and efficacy of Tre-D-Axitinib are evaluated in a mouse model of oxygen induced retinopathy (OIR) *via* intraperitoneal (IP) administration of the treatment.

**Results:** The Tre-D demonstrates inherent targeting to neovascular tufts in an OIR mouse model. Tre-D-Axitinib leads to increased vaso-obliteration in the ischemic retina. The IP administration of Tre-D-Axitinib effectively reduces pathological retinal neovascularization, tuft formation, and vessel anastomoses while showing minimal off-target effects and rapid renal clearance. Mechanistic studies reveal that Tre-D-Axitinib inhibits VEGFA-induced proliferation, migration, and angiogenesis in human retinal endothelial cells.

**Conclusions:** To date, there are no organic nanoparticles that localize selectively in aberrant neovascular tufts at the site of pathology in retina when systemically administered. By eliminating the need for invasive intravitreal injections and addressing systemic toxicities, Tre-D-Axitinib introduces a novel systemic nanotherapeutic strategy with broad implications for treating ischemic retinopathies.

## Introduction

Proliferative retinopathy, also known as pathological retinal neovascularization (NV), is a significant contributor to moderate to severe vision impairment across various age groups. This condition manifests in several forms, that include retinopathy of prematurity (ROP) in infants, diabetic retinopathy (DR) in adults, and neovascular age-related macular degeneration (nAMD) in elderly people 1-3. The underlying mechanism of these diseases involves aberrant angiogenic responses to ischemia or hypoxia. However, this process often leads to pathological NV in the vitreous, resulting in interference with light transmission and subsequent vision impairment. This aberrant NV is driven by the high levels of vascular endothelial growth factor A (VEGFA), resulted due to hypoxia. One of the primary treatment modalities for advanced stages of retinopathy is laser photocoagulation 4. This technique effectively targets and destroys the abnormal blood vessels that contribute to further retinal damage. While laser treatment has proven effective in many cases, it is not without limitations. There are instances where the underlying pathological processes continue to progress despite treatment, emphasizing the urgent need for alternative therapeutic strategies 4.

The advent of intravitreal injections of anti-VEGFA agents has transformed the management of neovascular retinal diseases. These therapies specifically inhibit the action of VEGFA, a key player in the angiogenic process. VEGFA remains a key molecular target of many current anti-angiogenic regimens because it is the most well-studied angiogenic and permeability factor involved in proliferative retinopathies 5-8. However, the intravitreal injection procedure can be painful and carries risks of post-injection complications, such as elevated intraocular pressure, intraocular inflammation, and even endophthalmitis 9-14. Even after intravitreal delivery, anti-VEGF therapies face challenges in adequately reaching the sites of active angiogenesis, thereby limiting their therapeutic effectiveness.

Axitinib, a potent multi-receptor tyrosine kinase inhibitor (TKI), targets vascular endothelial cell growth factor receptor 1-3 (VEGFR 1-3), platelet derived growth factor receptor beta (PDGFR-β) and KIT receptors 15. Axitinib received the United States Food and Drug Administration (FDA) approval in 2012 for treatment of renal cell carcinoma. An injectable suspension of Axitinib is currently being investigated for the treatment of AMD using a suprachoroidal microinjector platform (NCT04626128). Multiple *in vivo* studies in mouse, rat, and rabbit models have demonstrated the therapeutic potential of Axitinib in treating NV within corneal, retinal, and choroidal tissues 16-21. Due to its potent pan-VEGFR inhibitory activity, Axitinib may offer advantages over current anti-VEGFA agents, which are associated with the upregulation of VEGF-C and VEGF-D 22. This compensatory upregulation of alternative VEGF ligands may contribute to tachyphylaxis and the development of refractory disease in clinical settings 21. Although Axitinib and related VEGF TKIs are effective in cancer therapy, their application in ocular NV is hindered by poor bioavailability in the posterior segment of the eye. Another reason Axitinib needs to be delivered precisely in the body is because it can lead to side-effects including hypertension, and cerebrovascular ischemic events 23-25.

Currently, a wide range of advanced therapeutic modalities, including topical formulations, systemic approaches, and other non-invasive technologies, are being actively investigated to address the challenges associated with intravitreal injections and to enhance the efficacy and durability of anti-VEGF-A therapies for the treatment of corneal and retinal NV 26-30. These innovative strategies seek to reduce the frequency of invasive intravitreal injections, enhance patient compliance, and minimize associated complications such as infection and retinal detachment. Additionally, nanotechnology offers a promising avenue for developing more effective treatment strategies by precise delivery of drugs to targeted locations 31, 32. Various nano-sized delivery systems, including polymers, dendrimers, nanowires, microneedles, and lipid-based nanoparticles, have shown potential in delivering therapeutic agents directly to specific cells in the retina and cornea 33-36. However, a majority of these interventions still rely on intraocular injections or local delivery methods, indicating a need for further development and refinement in nanotechnology applications for the ocular therapies. An ideal smart drug delivery platform for treating retinal NV would efficiently cross the blood-retinal barrier and selectively target aberrant neovascular areas through a minimally invasive route. It should be able to deliver anti-VEGFA therapy directly to the affected endothelial cells, remain localized at the injury site for extended periods, effectively suppress NV in the retina, and be efficiently cleared from systemic circulation to minimize off-target toxicity.

Among various nanoparticles, dendrimers represent excellent attributes for drug delivery applications 31, 32, 37-41. These tree-like hyperbranched and monodispersed macromolecules can be synthesized in a tailor-made fashion with complete control on the physiochemical and pharmacological properties. The peripheral groups can be harnessed to conjugate a variety of therapeutic molecules, imaging dyes, and targeting ligands 42-44. Currently, no organic nanoparticles have been shown to selectively target aberrant neovascular tufts at retinal pathology sites following systemic administration.

Here, we report a unique mixed layered trehalose-surfaced dendrimer (Tre-D), that is composed of biocompatible building blocks and, upon systemic administration in oxygen-induced retinopathy (OIR) mouse model, selectively targets aberrant neovascular tufts while showing no accumulation in normal retinal vasculature. We further conjugate Axitinib on the surface of Tre-D to develop Tre-D-Axitinib conjugate. Our findings indicate that a single intraperitoneal (IP) injection of Tre-D-Axitinib may help attenuate abnormal blood vessel growth or pathological angiogenesis in ischemic retinopathies by selectively targeting neovascular tufts. This could lead to better non-invasive treatment options for proliferative retinopathies. This targeted approach maximizes Axitinib's efficacy while minimizing systemic side effects associated with the free drug. In addition, it will create a new set of treatment options that may lessen the side effects of intravitreal injections.

## Materials and Methods

### Chemistry - experimental section

The details related to the synthesis of dendrimer and conjugates including instrumentation, materials, synthetic procedures, characterization, and formulation stability studies are provided in the supporting information file.

### *In vitro* studies - experimental section

#### Materials and reagents

Phosphate buffer saline (PBS), 4′,6- diamidino-2-phenylindole (DAPI), and 3-(4,5- dimethylthiazol-2-yl)-2,5 diphenyltetrazolium bromide (MTT), were procured from Aaron Chemicals. Dulbecco's Modified Eagle Medium (DMEM) was purchased from Cytiva, Fetal bovine serum (FBS) was obtained from Gibco Scientific. All the cell lines were procured from American Type Culture Collection (ATCC). Angiogenesis and BrdU cell proliferation assay kit was purchased from Abcam. All reagents were used as received with no further modification.

#### VEGFR2 inhibition studies

Tre-D and Tre-D-Axitinib conjugates were tested against KDR/VEGFR2 (Invitrogen, catalog# PR5992C) in 10-dose IC_50_ triplicate mode with a 3-fold serial dilution starting at 10 (Tre-D-Axitinib) or 100 μM (Tre-D) on dendrimer basis. The reference inhibitor, staurosporine, was assayed in a 10-dose IC₅₀ format with serial 4-fold dilutions starting from 20 μM. Reactions were performed at 10 (Tre-D-Axitinib) or 100 μM (Tre-D) ATP. Compounds were pre-incubated with kinase and substrate (pEY + Mn; Sigma, Catalog# P7244-250MG) mixtures at room temperature, and then the reaction was initiated by the addition of radioisotopically-labeled ATP (33P-ɣ-ATP). The reaction mixtures were spotted onto filter paper that selectively bound the radiolabeled catalytic product (³³P-substrate). Unreacted ³³P-ATP was removed by washing the filters. Kinase activity was quantified as the percentage of remaining activity in test samples relative to vehicle (dimethyl sulfoxide) controls. IC₅₀ values and curve fits were calculated using GraphPad Prism software. The experiments were performed in triplicates.

#### Cell culture

Human Retinal Microvascular Endothelial Cells (HRMVECs; #ACBRI 181), were purchased from Cell Systems, Kirkland, WA. We tested these cells for their EC authenticity by immunostaining them for CD31 and vWF. The HRMVECs were regularly checked for mycoplasma contamination. These HRMVECs were grown in EGM2 medium with amphotericin B (0.25 μg/mL) and gentamycin (10 μg/mL) and kept at 37°C in a humidified incubator (95% air and 5% CO_2)_. The cells were synchronized in a serum-free media for approximately 24 h to achieve quiescent state.

#### Cell uptake studies

For evaluating the mechanism of Tre-D and Tre-D-Axitinib uptake by HUVECs, a cell uptake study was carried out following our previously published protocols with some modifications 31,32

#### Cell viability assay

To evaluate the efficacy of the Tre-D-Axitinib conjugate under *in vitro/in vivo* settings, we performed primary cellular compatibility (3-(4,5-dimethylthiazol-2-yl)-2,5-diphenyltetrazolium bromide) (MTT) assay on two cell lines, Human umbilical vein endothelial cells (HUVECs) and macrophages (RAW-Blue) following previous reports 31, 32,45. We next seeded the grown cells on a 96 well plate (10^4^ cells/well). A CO_2_ incubator was then used to grow the cells overnight. The medium was aspirated, and cells were treated at different concentrations (1, 5, 25, and 50 µM) for 48 h. In the next step, we washed the cells using PBS solution. The cells were then incubated with 10 µL MTT (5 mg/mL) for ~ 3 h. The MTT was removed, followed by the addition of ~150 µL DMSO (culture grade) to each well. Finally, a UV-vis based read-out was taken at 570 nm to evaluate the cellular viability using BioTek, synergy microplate reader (Thermo Scientific). Experiments were performed in triplicates.



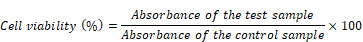



#### Angiogenesis assay

For evaluating the anti-angiogenesis efficacy of the Tre-D-Axitinib conjugate, an angiogenesis assay was performed *in vitro* according to the kit protocol using the Angiogenesis Assay Kit (ab204726). Briefly almost 50 µL of the provided kit matrix (ECM) was added to the each of the black colored clear bottom 96 well-plate for 1 h at 37 ℃ in a CO_2_ incubator, till it gets solidified. The cultured HUVEC cells were seeded into these wells at a density of 1×10^5^ cells/well and grown overnight at 37 ℃. The following day, these cells were incubated with the corresponding Tre-D-Axitinib and free Axitinib at different concentrations (1, 5, 25 and 50 µM), and dendrimer control (Tre-D, 50 µM) for 12 h at 37 ℃ along with the control wells with added matrix. The media was carefully aspirated without disrupting the matrix and washed with the provide elution buffer. Finally, the cells were stained with the dye from the kit for ~30 min at 37 ℃ and imaging was carried out using inverted fluorescence microscope (Ziess Axiovert fluorescence microscope at 5x magnification). The images were captured using a digital camera mounted to the microscope by zooming out to get the image of the whole wells. After the imaging was done, the images were analyzed using Carpentier et al.'s (2020) angiogenesis plug for ImageJ software 46, (NIH, v1.54j). The interaction between cells was expedited using ImageJ-programmed macro language for visualizing the micrographs. The plug-in automatically identified and analyzed different angiogenic parameters including tube length, junctions, branches, meshes, segments and extremities for determining the angiogenic capacities of corresponding dendrimers onto the HUVEC cells. All the measurements were performed in triplicates.

#### Cell proliferation assay

For evaluating the effect of dendrimers on the cell proliferation of the HUVEC cells, a kit-based BrdU cell proliferation assay was performed. For this, almost 2×10^5^ cells were seeded in a 96-well plate and put in a CO_2_ incubator for growth (37 °C, 5% CO_2_). Following this, the HUVECs were treated with the Tre-D-Axitinib conjugate at different concentrations (1, 5, 25 and 50 µM) along with the same concentration of Axitinib, and the highest concentration of Tre-D control (50 µM) for 12 h. Approximately 20 µL of BrdU reagent was added to all the sample wells and incubation was done for 25 min in CO_2_ incubator. The sample solution was carefully aspirated, and wells were washed properly using plate wash buffer. We, next, fixed the cells using the fixing solution provided in the kit for 20 min followed by washing again. For each of the sample wells, 100 μL anti-BrdU detector antibody was added, and incubation was done for 1 h at 37 °C. This was then followed by washing and gentle drying afterwards. After this, 100 μL/well goat anti-mouse IgG peroxidase antibody was added to the wells. Further, all solutions were aspirated, and the wells were then washed several times and dried properly. To each well, 100 μL of chemiluminescent substrate was applied and incubated in the dark at RT for ~10 min. The luminescence reading was immediately taken using a Synergy H1 hybrid multi-mode microplate reader (BioTek Instruments). All the measurements were carried out in triplicates; with proper controls, blank (media alone) and background (cells only, no BrdU reagent).

HRMVEC proliferation was evaluated using BrdU Flow Kit (#559619, BD Biosciences). The HRMVECs were grown in 60-mm petri dishes (2 × 10⁶ cells/mL) until the desired confluence. The cells were further treated with BrdU ( 1mM solution) for 2 h at 37 °C. In the next step the cells were fixed and permeabilized followed by washing with BD Perm/Wash™ buffer and incubated on ice for 15 min. Following an additional wash, cells were fixed again with BD Cytofix/Cytoperm™ buffer for 5 min at RT. DNA was then exposed by incubating the cells with DNase solution for 1 h at 37 °C. After washing, the cells were incubated with 50 μL of FITC-conjugated anti-BrdU antibody for 20 min at RT. They were then washed again, treated with 20 μL of 7-AAD solution, and gently suspended in 1 mL of staining buffer for analysis. The prepared samples were subsequently analyzed on a BD LSR II flow cytometer.

#### Cell migration assay

The effect of Tre-D-Axitinib and free drug on the migration of HRMVECs and HUVECs in presence or absence of VEGFA were carried out using scratch/cell migration assay following the published reports with slight modifications 47. In brief, 2 × 10^5^ cells were seeded in a 12 well plate and grown overnight in a CO_2_ incubator maintaining the temperature at 37 ℃. The media was aspirated, cells were washed using 1X PBS and a clean scratch was made using 200 µL micropipette tips manually followed by washing again to remove the scratched cells. Fresh media (supplemented with ~1% FBS) was added to the cells along with the dendrimers at corresponding concentrations (1, 5, 25 and 50 µM) were incubated with this media. Light microscopy imaging was performed at 0, 24 and 48 h intervals to see the changes in the migration behavior under the influence of dendrimers, using Zeiss Axiovert 135 TV microscope or EVOS M5000 Imaging System at 4x magnification (Thermo Fisher Scientific, Waltham, MA). The anti-migration efficacy of the dendrimers was evaluated using ImageJ (NIH, v1.54j) plugin with high throughput image analysis of *in vitro* scratch. Samples triplicates were taken with proper controls. Wound closure percentage was calculated as: [total wound area at 0 h - wound area at 24 h/total wound area at 0 h] x 100.

#### Spheroid sprouting assay

The three dimensional spheroid sprouting assay was evaluated in the HRMVECs following our previously published literature 48. In this procedure, HRMVECs were first stained with BCECF-AM (10 μM). To generate spheroids containing approximately 400 cells each, 8×10⁴ cells were suspended in 4 mL of EBM2 medium supplemented with 20% methocel. After this, 25 mL of cell suspension were added dropwise on a 150 mm culture plate and incubated for 24 h in a CO_2_ incubator at 37°C. Next, the spheroid grown were washed with 1X PBS (pH 7.4) and stored in ice-cold methocel containing 10% FBS. Following this, 4 ml of a collagen solution (1 mg/ml in EBM2) was mixed with the spheroid solution, and 250 µL of the resulted solution was pipetted in a 24-well cell culture plate and incubated for 1 h at 37°C in CO_2_ incubator for collagen to solidify/polymerize. Following 1 h incubation, each of these wells that had spheroids implanted, 250 µL of EGM2 medium was added. The plate was kept in CO_2_ incubator at 37°C for 48 h. Finally, the spheroids were comprehensively imaged using confocal laser scanning microscopy on Zeiss LSM 800 confocal microscope.

#### Tube formation assay

For evaluating the effect of developed dendrimer conjugate on endothelial cell angiogenesis, growth factor-reduced matrigel (Cat no. 35420, Corning Life Sciences) was utilized 49. We transfected control and specific siRNAs into HRMVECs, quiesced, suspended them, and then added them to a 24-well Matrigel-coated culture dish and grown for 6 h at 37°C in a CO_2_ incubator. The tube formation was evaluated using a digital inverted microscope (EVOS M5000 imaging system, Thermo Fisher Scientific). After qualitative microscopy, the micrographs were quantitatively analyzed using NIH ImageJ (1.43) software to measure the tube lengths.

### *In vivo* studies - experimental section

#### Experimental animals

For the *in vivo* assessment of the Tre-D-Axitinib dendrimer, C57BL/6J mice were obtained from The Jackson Laboratory (Maine, USA). The animals were housed and bred in the animal facility (Wayne State University DLAR) in Detroit, Michigan, USA. They were provided with unlimited access to food and water and maintained under a 12-h light-dark cycle. The study involved both male and female mouse pups between postnatal day 12 (P12) and postnatal day 17 (P17). All experimental procedures were reviewed and approved by the Institutional Animal Care and Use Committee of Wayne State University (IACUC-23-07-5936) and were carried out in strict compliance with the National Institutes of Health (NIH) Guide for the Care and Use of Laboratory Animals.

#### Sex as a biological variable

In our study, we used both male and female mice. We have not observed any differences in both sexes and thus did not considered sex as a biological variable.

#### Intraperitoneal (IP) injections

Mice pups were IP administered with 50 µL of Tre-D, or Tre-D-Cy5, or Tre-D-Axitinib or Tre-D-Axitinib-Cy5 at P12 using a 30 G needle.

#### Immunofluorescence staining

After hyperoxia exposure, mouse pups received an intraperitoneal injection (IP) of Tre-D-Cy5 at postnatal day 12 (P12). At P17, the pups were euthanized, and the eyes were collected and embedded in optimal cutting temperature (OCT) compound. Retinal cryosections (10 μm thick) were prepared from the central region. To determine the distribution of dendrimers in the retina, sections were immunostained with rat anti-mouse CD31 (1:100) followed by Alexa Fluor 488-conjugated secondary antibodies. The stained sections were imaged using a Zeiss LSM 800 confocal microscope, and images were processed with Zen software (Carl Zeiss Imaging Solutions GmbH).

#### Histology examination

At P17, the livers and kidneys of mice exposed to OIR were fixed in phosphate-buffered formaldehyde (4% v/v), and then were embedded in OCT. The cryosections were prepared and stained with hematoxylin and eosin (H&E).

#### Oxygen-induced retinopathy (OIR)

At postnatal day 7 (P7), mouse pups and their dams were placed in a BioSpherix chamber for five days, where they were exposed to 75% oxygen (from P7 to P12). After this period, they were returned to room air. Age-matched littermates that remained in room air (21% oxygen) throughout the experiment served as controls. On P17, the pups were euthanized, and their eyes were collected and fixed. The retinas were carefully dissected, stained with rhodamine-labeled isolectin B4, and subsequently flat-mounted for confocal imaging. Retinal NV was quantified using Nikon NIS-Elements Advanced Research software. Neovascular areas were pseudocolored in red, and the extent of NV was calculated as the ratio of fluorescence intensity within the neovascular regions to the total retinal fluorescence intensity, expressed as a percentage. The avascular area (%) was determined by dividing the avascular region by the total retinal area and multiplying by 100. Mouse pups weighing less than 6 g at P17 were excluded from the analysis, and the weights of all included pups were within a 0.5 g range. To minimize bias, researchers performing the outcome assessments, including lesion evaluation, were blinded to the group assignments.

#### Liver and kidney function analysis

C57BL/6J mouse pups were subjected to OIR, and at postnatal day 12 (P12), the pups received an IP injection of 50 µL of phosphate-buffered saline (PBS) and Tre-D-Axitinib-Cy5 (20 mg/kg Bwt). At P17, blood samples from the mouse pups were collected, and serum alanine aminotransferase activity (ALT, E-BC-K235-M, Elabscience, Houston, Texas, USA), aspartate aminotransferase activity (AST, E-BC-K236-M, Elabscience), and creatinine levels (E-BC-K188-M, Elabscience) were quantified in accordance with the manufacturer's instructions. The ALT and AST activity were measured in IU/L (international units per liter), while creatinine levels were measured in μmol/L.

### Statistics

All experiments were conducted at least three times. Data are presented in the graphs as mean ± standard deviation (SD). The normality of data distribution was evaluated using the Kolmogorov-Smirnov test with Lilliefors' correction before performing parametric analyses. Differences between two groups were analyzed using unpaired t-tests, while comparisons among multiple groups were assessed using one-way ANOVA followed by Bonferroni's post hoc correction. Statistical analyses were performed using GraphPad Prism 9, and a p-value of < 0.05 was considered statistically significant.

### *Ex vivo* dendrimer quantification

All major organs were slowly thawed on ice and then weighed. Defined portions of tissues from each organ were weighed and collected in Eppendorf tubes. The tissues were homogenized in methanol at a ratio of 1 mL per 100 µg of tissue using stainless steel beads and a tissue homogenizer. Following homogenization, the tubes were centrifuged at 4 °C. The supernatant was collected in protein LoBind tubes and was stored at -80 °C. The fluorescence intensity was measured using a Fluoromax spectrofluorophotometer. Cy5 fluorescence (λex = 645 nm, λem = 662 nm) was recorded and corrected for background signals from control tissues. Fluorescence values were then quantified using calibration curves of Tre-D-Axitinib-Cy5 generated at different slit widths.

## Results and Discussion

In this study, we aim to design an innovative dendrimer-based smart, systemic, and minimally invasive delivery system that can precisely deliver anti-VEGFA therapies to diseased NV areas in retina. This approach seeks to replace current painful regimen of intraocular injections of anti-VEGF drugs, which are the first-line treatment for retinal NV. The complexity in the design of nanomedicines is a major bottleneck for their large-scale production and scalability and often lead to failure during clinical development. Therefore, it is crucial to rationally design disease-directed nanotherapies with simplified yet efficient synthetic approaches to ensure their rapid translation from bench to bedside.

### Rationale for the development of Tre-D technology

**Figure 1:**
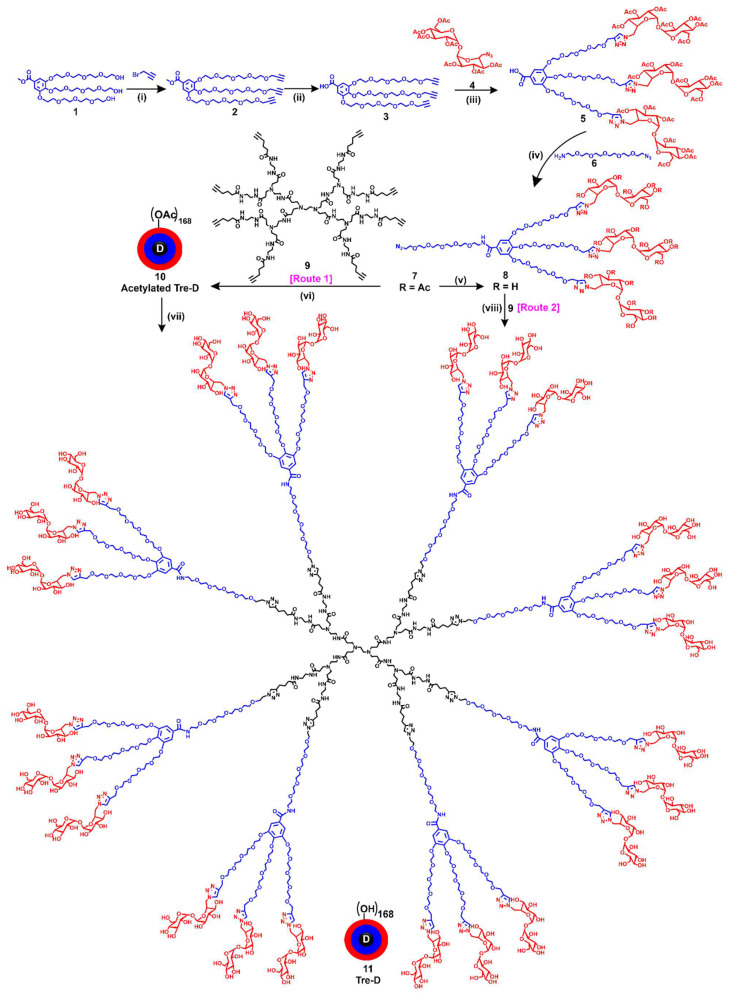
** Synthesis of Trehalose dendrimer (11).** *Reagent and conditions* (i) DMF, NaH, RT, 3 h, 75% (ii) LiOH^.^H_2_O, THF-H_2_O, RT, 24 h, 78% (iii) Copper(II) sulfate pentahydrate, sodium ascorbate, DMF, 50 ^o^C, 12 h, 90% (iv) EDC^.^HCl/HOBt, DCM, RT, 1.5 h 85% (v) Sodium methoxide, DCM, MeOH, RT, overnight, 92% (vi) Copper(II) sulfate pentahydrate, sodium ascorbate, DMF, 40 ^o^C, MW, 15 h, 85% (vii) NaOMe, MeOH, RT, 16 h, 90% (viii) Copper(II) sulfate pentahydrate, sodium ascorbate, DMF, 80 ^o^C, MW, 15 h, 86%.

Chemically, the Tre-D is composed of three distinct layers at each generation **(Fig. 1)**. Tre-D is a second-generation (G2) dendrimer where the first generation polyamidoamine (PAMAM) dendrimer serves as the central core, the second generation is constructed from biocompatible gallic acid-derived building units, and trehalose (a disaccharide of glucose) molecules. We used trehalose as the peripheral layer of Tre-D for several reasons. Trehalose is a biocompatible, FDA approved material which is used in pharmaceuticals as a stabilizing agent due to its ability to stabilize biological molecules and proteins. More specifically, among the various biocompatible sugar-based materials, we selected trehalose for the development of Tre-D dendrimer platform for Axitinib delivery to neovascular tufts due to its well-documented inhibitory effects on VEGF-induced angiogenesis and vascular proliferation 50. Trehalose has also been shown to suppress fibroblast proliferation by inducing apoptosis and to reduce conjunctival NV 51. Additionally, trehalose has been a part of many eye-drop formulations to provide and enhance their shelf-life stability 52, 53.

Tre-D is constructed in fewer reaction steps, maintaining orthogonality, enabling the rapid formation of highly dense and structurally uniform dendrimer (**Fig. 1**). In contrast, traditional dendrimer synthesis methods for producing commercially available dendrimers are often time-consuming, labor intensive, low yielding, and synthetically complex. For example, the synthesis of a widely used fourth-generation PAMAM dendrimer typically requires 6 to 8 weeks of meticulous work, involving 8 to 10 reaction steps to generate 64 hydroxyl groups at the surface 54. To overcome these challenges, we developed an expedited convergent synthetic methodology, incorporating mixed layers of building blocks at each dendrimer generation. This design features an 8-armed core and a dendron with 21 hydroxyl groups, efficiently connected using highly efficient Cu(I)-catalyzed azide-alkyne cycloaddition (CuAAC) reaction. We have previously demonstrated the use of CuAAC to assemble complex dendritic structures without structural defects, an achievement that remains a significant challenge with commercially available dendrimers 31, 32, 37. We designed the Tre-D around biocompatible and GRAS building units, selected to mitigate the risk of cytotoxicity or other adverse effects. The dendrimer is composed of ~38 wt% trehalose, ~36 wt% short oligoethylene glycol (OEG), ~10 wt% triazole, ~10 wt% core, and ~6 wt% gallic acid.

The developed procedure for Tre-D synthesis is significantly faster and more efficient than conventional dendrimer synthesis approaches reported in the literature, which are typically time-consuming, multi-step, and low yielding. This streamlined approach aligns with sustainability goals, promoting safer and more eco-friendly practices in the manufacturing of pharmaceuticals. The presence of trehalose, a highly water-soluble disaccharide, at dendrimer surface resulted in a high solubility of Tre-D as approximately 800 mg/mL in aqueous solutions. Apart from bringing hydrophilicity in the molecule, the resulting Tre-D carries 168 hydroxyl groups on the periphery of the dendrimer which can be modified by variety of chemistries for the attachment of drugs and therapeutics, biomolecules, proteins, antibodies, or genes. Moreover, we use stable chemical linkages in the dendrimer backbone to avoid degradation under physiological conditions and provide intact clearance of the dendrimer (~5 nm) from off-target organs.

### Synthesis and characterization of Tre-D

**Figure 2:**
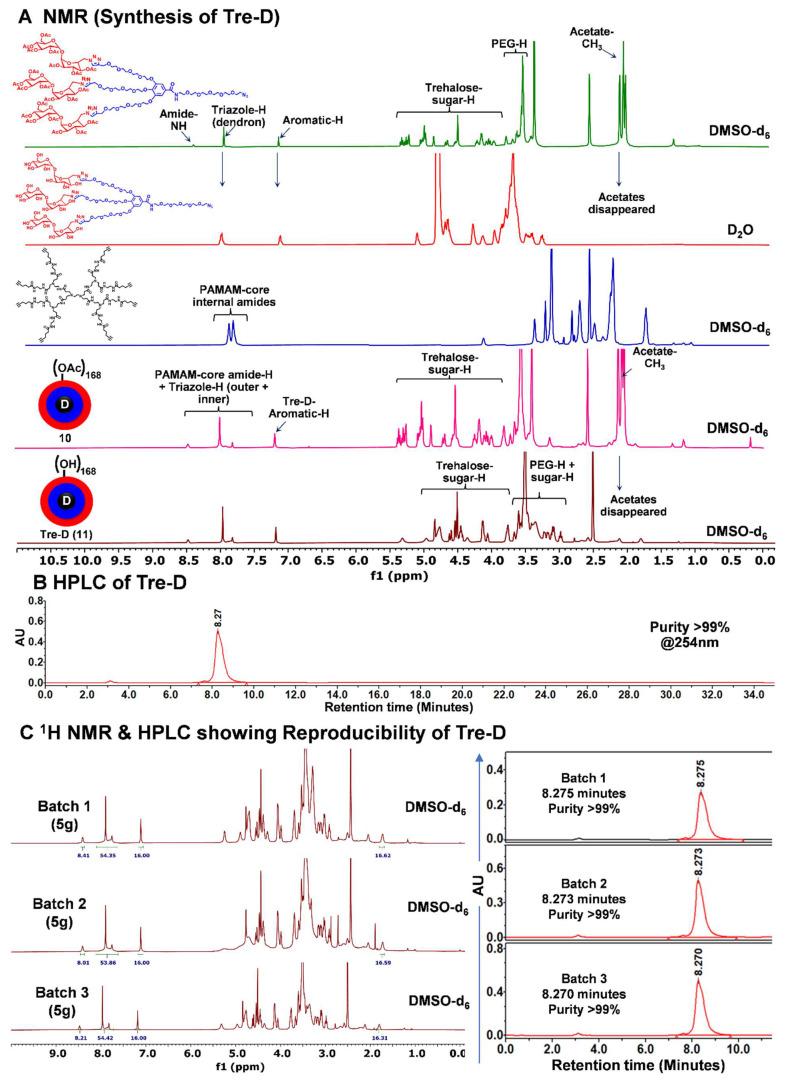
**NMR and HPLC characterization of Tre-D. (A)** ^1^H NMR spectra of intermediate dendrons, hyper-core, protected and deprotected Tre-D dendrimers showing the disappearance and appearance of characteristic protons; **(B)** HPLC chromatogram of Tre-D showing >99% purity; **(C)** ^1^H NMR spectra and HPLC traces demonstrate reproducibility in the structure and purity among three different (5g) batches of Tre-D.

The synthesis pathway towards Tre-D started with the preparation of a clickable dendron **8**, as depicted in **Fig. 1**. The process initiated with the propargylation of tri-hydroxyl gallate (**1**) using sodium hydride in anhydrous DMF, yielding compound **2**. The successful synthesis of compound **2** was confirmed by ^1^H NMR, which exhibited a characteristic alkyne peak (3H) at δ 2.43 ppm (**Fig. S1**). In the next step, the hydrolysis of the methyl ester **2** afforded compound **3** in 78% yield. Next, the CuAAC reaction between tri-propargyl gallic acid **3** and peracetylated trehalose azide **4** afforded compound **5**. The successful completion of the click reaction was confirmed by the disappearance of the alkyne proton signal at δ 2.31 ppm and the emergence of a characteristic triazole proton resonance (3H) at δ 8.00 ppm in the ¹H NMR spectrum. (**Fig. S8**). Additionally, the ^1^H NMR spectrum showed distinct acetate protons (63H) corresponding to the three trehalose sugars, appearing at δ 1.95-2.05 ppm. Subsequently, the EDC coupling between carboxylic acid **5** and azido-PEG-5-amine **6** afforded dendron **7** in 85% yield. The successful formation of compound **7** was confirmed by the presence of a characteristic amide proton at δ 8.46 ppm, along with signals from the OEG protons in the ^1^H NMR spectrum (**Fig. 1 and 2A**). De-*O*-acetylation of dendron 7 was carried out under Zemplén transesterification conditions using sodium methoxide in methanol, yielding the fully deprotected trehalose dendron 8 in 92% yield. The successful completion of the reaction was verified by the disappearance of acetate protons in the δ 1.95-2.04 ppm region (**Fig. 1 and 2A**). Next, we synthesized the 8-armed alkyne-functionalized G-1 PAMAM core (**9**) using our previously published procedure 31.

The characterization of dendrimers poses a significant challenge in synthetic chemistry due to their complex, highly branched structures and presence of huge number of protons which often produce dense and overlapping signals in ^1^H NMR spectra. Identifying key peaks in spectroscopic analysis is crucial for verifying the structural integrity and composition of the synthesized macromolecule. For ease of characterization, we synthesized Tre-D using two synthetic routes. We first opted to perform the reaction using acetate-protected trehalose azide dendron (**7**) (*Route 1*, **Fig. 1**). In the protected synthetic route, the octa-alkyne G-1 PAMAM dendrimer core (**9**) and the acetate-protected trehalose-azide dendron (**7**) was clicked together using CuAAC reaction. Conventional CuAAC conditions were employed where a catalytic amount of CuSO_4_·5H_2_O (5 mol % per alkyne) and sodium ascorbate (10 mol % per alkyne) was used, and the reaction was conducted under microwave (MW) irradiation at 40 °C for 15 h. This method yielded acetate-protected Tre-D (**10**) in 90% yield. The successful formation and the structure of the product were confirmed by the proton NMR showing the presence of triazole protons at δ 7.93 ppm and acetate protons at δ 1.82-2.00 ppm, corresponding to the attachment of 24 trehalose sugar units (**Fig. 1, 2A and S17**). This approach was particularly advantageous in characterizing the dendrimer, as the huge peaks from acetate protons occupy a region in the ^1^H NMR spectrum that avoids signal interference.

The acetates were removed under Zemplén transesterification conditions using NaOMe in methanol, yielding the final G2 Tre-D (**11**) with 168 hydroxyl terminal groups in 90% yield. The complete deacetylation was confirmed by the ¹H NMR spectrum, which showed the disappearance of acetate proton signals, indicating the formation of the fully deprotected product (**Fig. 1, 2A and S19**). For route 2, we performed the CuAAC reaction between the core (**9**) and the deprotected trehalose-azide dendron (**8**) under similar conditions (*Route 2*, **Fig. 1**), leading to the formation of the final Tre-D (**11**). Both synthetic routes using protected and deprotected dendrons consistently produced excellent and reproducible results. Purification of the final Tre-D was accomplished through dialysis using a 3.5 kDa membrane. HPLC analysis of the dendrimer (**11**) confirmed purity exceeding 99% (**Fig. 2B**). The characterization of Tre-D and its intermediates was conducted using a range of techniques, including ^1^H NMR, ^13^C NMR, HPLC, MALDI-TOF, and HRMS (**Fig. S1-S22**). As shown in **Fig. 2A**, the ^1^H NMR spectra display the appearance and disappearance of characteristic peaks corresponding to various intermediates, which simplify the interpretation of the dendrimer's structure. Tre-D demonstrates exceptional water solubility (~800 mg/mL), which makes it a promising nanocarrier for hydrophobic drugs, hence eliminating the need for additional excipients for final drug products.

One of the primary challenges in translating nano-therapeutics into clinical applications is developing synthesis processes that are both reproducible and scalable. To overcome the batch-to-batch related heterogeneity associated with macromolecular synthesis, we kept the design of Tre-D very simple and straightforward, and stitched the building blocks together with highly facile and efficient chemical transformation, CuAAC. To evaluate the reproducibility of Tre-D synthesis, we produced several 5-gram-scale batches of Tre-D and compared their ^1^H NMR spectra along with purity *via* HPLC analysis. The ^1^H NMR spectra from three independent batches (**Fig. 2C**) consistently confirmed the presence of 24 trehalose dendrons on the surface, indicating the formation of defect-free dendrimer. Additionally, the HPLC chromatogram of each batch revealed peaks at the same retention time (8.2 minutes), further confirming batch-to-batch purity (**Fig. 2C**)**.** The high and consistent yields across batches confirm the robustness of this synthetic method, suggesting its scalability for potential clinical translation.

**Figure 3:**
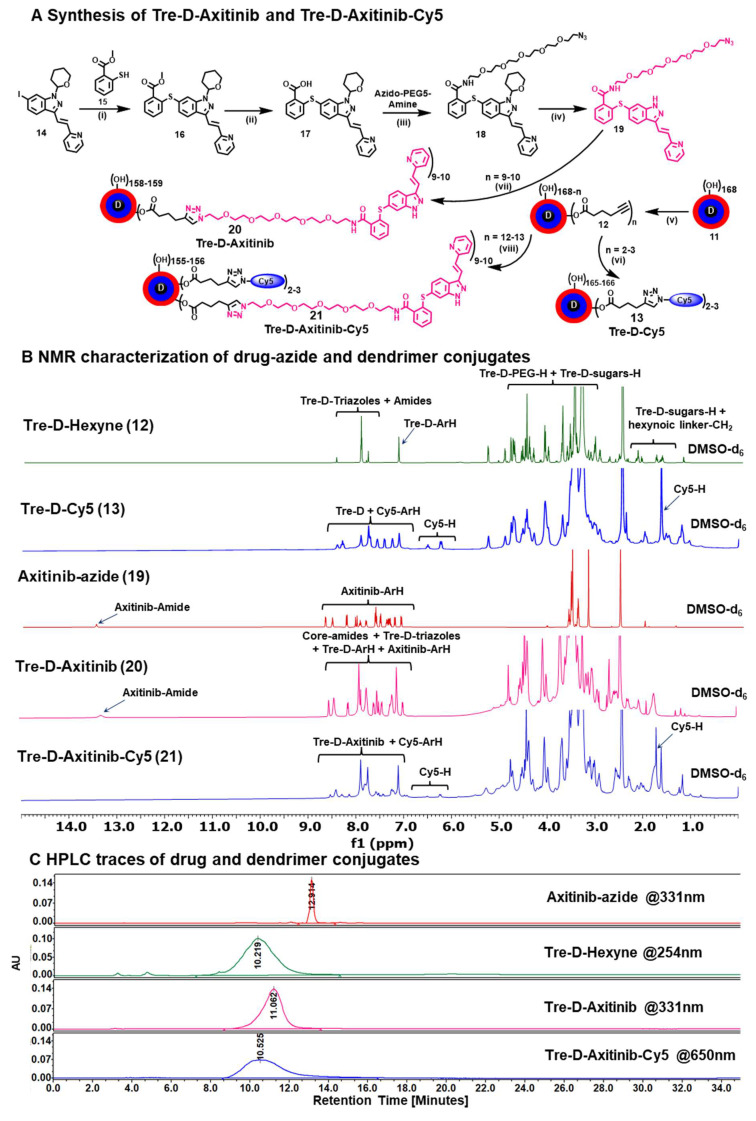
**Synthesis and characterization of fluorescently labeled and unlabeled TreD-Axitinib conjugates. (A)** Synthesis of Tre-D-Cy5, Axitinib azide, Tre-D-Axitinib and Tre-D-Axitinib-Cy5 conjugates*, Reagents and conditions:* (i) Pd(dppf)_2_Cl_2˙_DCM, K_3_PO_4_, DMF, 80 ^o^C, 12 h, 82%; (ii) NaOH, MeOH:THF:H_2_O, RT, 2 h, 90%; (iii) HATU, DIPEA, DMF, RT, 1 h, 70%; (iv) TFA, 60 ^o^C, 1 h, 75%; (v) Hexyn-5-oic acid, EDC.HCl, DMAP, and DMF, 24 h, RT, 90%; (vi) Cy5-azide, Copper(II) sulfate pentahydrate, sodium ascorbate, DMF:H_2_O, 40 ^o^C, 10 h, 89%, (vii) Copper(II) sulfate pentahydrate, sodium ascorbate, DMF:H_2_O, RT, 24 h, 86%; (viii) Copper(II) sulfate pentahydrate, sodium ascorbate, DMF:H_2_O, RT, 24 h, 88%; **(B)**^ 1^H NMR stacked spectra of Tre-D-Hexyne (**12**), Tre-D-Cy5 (**13**), Axitinib-azide (**19**), Tre-D-Axitinib (**20**), and Tre-D-Axitinib-Cy5 (**21**) conjugate showing the appearance of characteristic protons; **(C)** HPLC chromatogram of Axitinib-azide (red), Tre-D-Hexyne (green) and Tre-D-Axitinib (pink), and Tre-D-Axitinib-Cy5 (blue) conjugates showing significant shift in retention time and >99% purity.

For *in vitro* and *in vivo* assessment of cellular uptake and organ biodistribution, Tre-D was surface labeled with the near-infrared fluorescent dye cyanine 5 (Cy5). The attachment of the Cy5 tag was achieved by introducing 3-4 linker arms of 5-hexynoic acid through an esterification reaction with the hydroxyl groups at the periphery of Tre-D. This reaction utilized EDC as a coupling reagent, resulting in the formation of compound **12** (**Fig. 3A**). The successful introduction of the 5-hexynoic linker was confirmed *via* ^1^H NMR analysis, which revealed additional methylene protons in the aliphatic region of the dendrimer (**Fig. S23**). HPLC analysis further demonstrated a change in retention time, shifting from 8.2 minutes for Tre-D to 10.2 minutes for the modified Tre-D-Hexyne (**12**), confirming the successful attachment (**Fig. S24**). In the next step, the acetylene-functionalized Tre-D-Hexyne (**12**) was conjugated with azide-terminated Cy5 using a CuAAC click reaction, yielding the fluorescently labeled Tre-D-Cy5 (**13; Fig. 3A**).

The successful conjugation of Cy5 to Tre-D was verified by the appearance of characteristic Cy5 proton peaks in the ^1^H NMR spectrum (**Fig. 3B**). Quantitative estimation of the number of Cy5 molecules linked to each dendrimer molecule was performed by proton integration, indicating the successful attachment of approximately 2-3 Cy5 molecules (**Fig. 3B and S25**). Following conjugation, Tre-D-Cy5 exhibited an HPLC chromatogram at 650 nm, corresponding to the absorption wavelength of Cy5, with a purity greater than 99%. Additionally, the HPLC retention time shifted from 10.2 minutes to 9.4 minutes upon Cy5 conjugation (**Fig. S26**).

### Synthesis and characterization of Trehalose dendrimer-Axitinib (Tre-D-Axitinib) conjugate

Next, we synthesized Tre-D-Axitinib, a conjugate of Tre-D with Axitnib. Axitinib is a highly potent TKI, known for its selective inhibition of VEGFR 1, 2, and 3, as well as other kinases such as c-KIT and PDGFRα/β, which are implicated in angiogenesis 55. Axitinib is investigated as administered *via* intraocular routes for treating eye-related disorders due to its inability to cross the blood-retinal barrier. Moreover, its clinical use is hampered by poor water solubility (0.2 μg/mL) and side-effects. By using Tre-D dendrimers for targeted delivery of Axitinib to aberrant neovascular tufts, we aim to develop non-invasive therapies for retinal disorders.

To synthesize the Tre-D-Axitinib (Tre-D-Axitinib) conjugate (**20**), we initiated by partially modifying the hydroxyl groups of the Tre-D through esterification with 5-hexynoic acid (**Fig. 3A**). This reaction introduced approximately 12 acetylene arms (**12**) into the dendrimer structure which was confirmed *via* ^1^H NMR and HPLC. For the subsequent conjugation of Axitinib to Tre-D, a clickable linker preferably having terminal azide functionality was required on Axitinib, which posed a challenge due to the lack of functional handle on it. An in-depth analysis of Axitinib's structure-activity relationship reveals that the pyrazole core, which interacts with the hinge region of the VEGFR2 domain, plays a crucial role in its anti-angiogenic activity 56. Literature reports indicate that modification at the methyl amide portion of the molecule is well-tolerated and maintains Axitinib's anti-angiogenic properties 57. Thus, we strategically modified the methyl amide bond on the phenyl ring of Axitinib through amide coupling with azido-PEG5-amine, preserving the amide bond's interaction with VEGFR2 while adding an azide terminating linker. This is worth noting here that the conjugation of Axitinib in Tre-D-Axitinib conjugate is through non-cleavable amide bond and the Tre-D-Axitinib conjugate is designed as an anti-VEGF therapy.

The synthesis of the azide-terminated Axitinib analogue (**compound 19**) is depicted in **Fig. 3A**. To introduce an azide linker *via* an amide bond into the Axitinib structure, the synthesis began with the palladium-catalyzed cross-coupling of commercially available aryl iodide compound **14** with methyl-2-mercaptobenzoate **15** using Pd(dppf)Cl_2˙_DCM in DMF, affording methyl benzoate **16** in 73% yield. The structure of compound **16** was confirmed by the presence of methyl ester protons at δ 3.89 ppm in the ^1^H NMR spectrum and the expected mass peak. Hydrolysis of ester **16** with sodium hydroxide yielded the free carboxylic acid **17**. EDC-mediated coupling of **17** with azido-PEG5-amine afforded compound **18** in 76% yield, as confirmed by the disappearance of the methyl ester signal and the appearance of OEG protons in the ^1^H NMR spectrum. Finally, deprotection of the tetrahydropyran group on the pyrazole using trifluoroacetic acid afforded compound **19** in 70% yield. The structure of compound **19** was validated by the disappearance of the THP signals and the appearance of the NH proton in the pyrazole ring at δ 13.35 ppm, along with OEG protons in the range of δ 3.40-3.75 ppm (**Fig. 3B and S37**). The spectral details (NMR, HPLC, and mass spectrometry) for the intermediates and the final conjugates are presented in the supporting information **(S27-S39)**.

After synthesizing azido-PEG5-Axitinib (**19**), we carried out its click reaction with Tre-D-hexyne (**12**) using previously established CuAAC conditions, successfully obtaining the Tre-D-Axitinib (**20**) conjugate (**Fig. 3A**). Purification was performed by dialysis using a 3 kDa membrane, first in 20% DMF/H_2_O to remove small-molecule impurities, followed by final dialysis in water, affording Tre-D-Axitinib in 86% yield. The characterization of Tre-D-Axitinib (**20**) conjugate was performed using proton integration method, with the sugar protons of Tre-D-Hexyne **12** (n=~12), observed at δ 1.71-1.80 ppm, serving as a reference (**Fig. 3B**). This analysis indicated that approximately ~9 Axitinib molecules were conjugated to each dendrimer, as confirmed by ¹H NMR spectroscopy (**Fig. 3B** and **S40**). Additionally, we evaluated the purity of Tre-D-Axitinib through HPLC, which demonstrated a purity level exceeding 99% (**Fig. 3C and S43**). There was a clear shift in the retention time upon conjugation of Axitnib-azide (12.9 minutes) to Tre-D-Hexyne (10.2 minutes) to afford Tre-D-Axitinib (11.0 minutes). We next attached a fluorescent tag Cy5 to Tre-D-Axitinib to investigate the cellular uptake mechanisms. The synthesis of Tre-D-Axitinib-Cy5 (**21**) was accomplished *via* a click reaction between Cy5 azide and the alkyne groups on the Tre-D-Axitinib conjugate (**20**). The confirmation of Cy5 attachment was performed through ^1^H NMR analysis, which revealed the presence of Cy5 protons, indicating that approximately two Cy5 molecules were linked to the dendrimer surface (**Fig. 3B and S44**). HPLC analysis demonstrated purity exceeding 99%, with a notable retention time shift from 11.0 minutes to 10.5 minutes after Cy5 conjugation (**Fig. 3C** and** S45**). Additionally, we monitored the HPLC chromatogram of all major intermediates and final dendrimer conjugates at multiple wavelengths [210 (dendrimer), 254 (aromatic), 331 (axitinib), and 650nm (Cy5)] to detect any impurities related to trehalose, axitinib, or Cy5 (**Fig. S46**), suggesting highly pure compounds.

**Figure 4:**
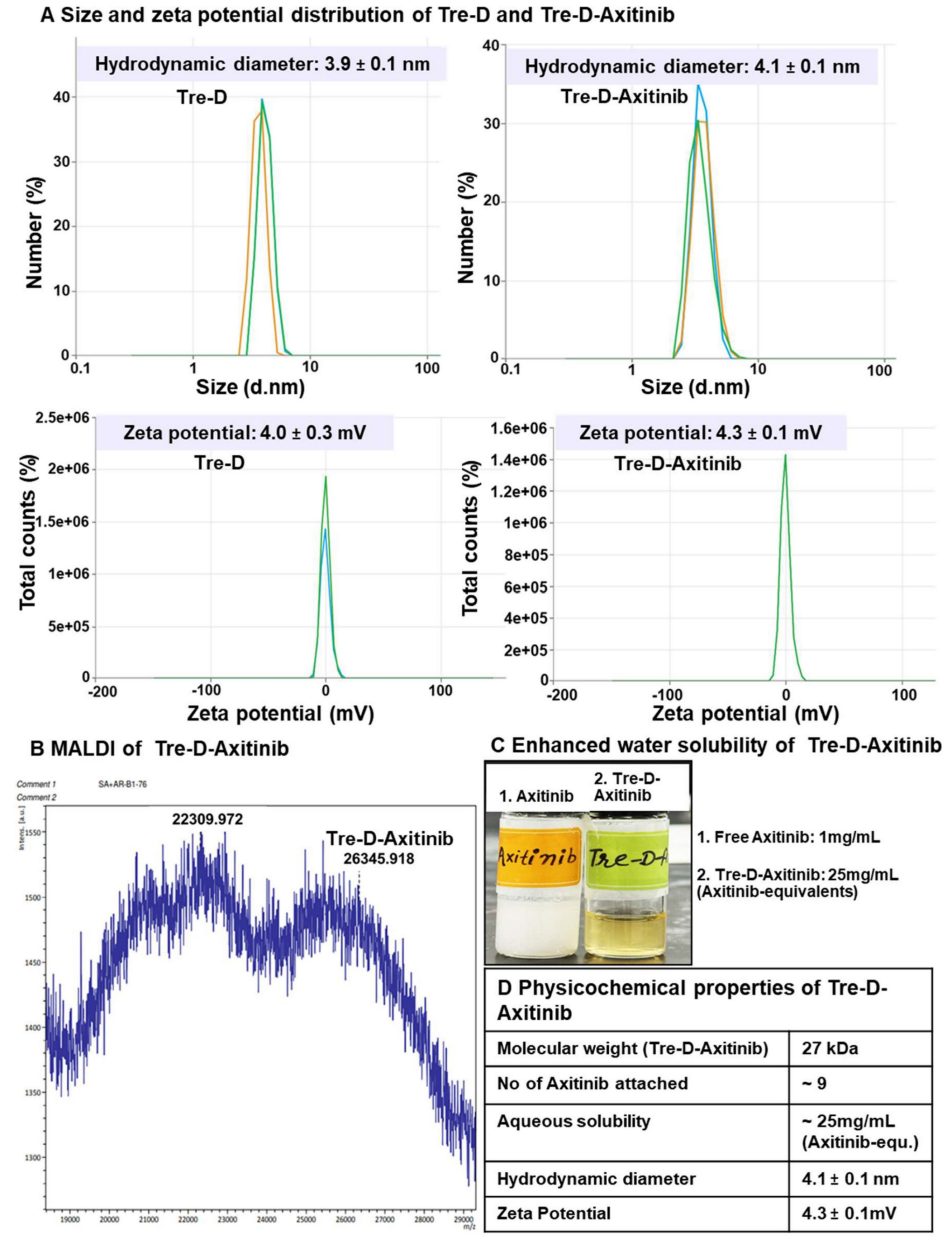
**Physiochemical characterization of Tre-D-Axitinib conjugate. (A)** Size and zeta potential distribution of Tre-D and Tre-D-Axitinib analyzed by dynamic light scattering (DLS) in triplicates; **(B)** MALDI-TOF of Tre-D-Axitinib **(C)** Tre-D-Axitinib has several folds higher water solubility than free Axitinib; **(D)** Table representing physicochemical properties of Tre-D-Axitinib.

Dynamic light scattering (DLS) analysis revealed that Tre-D has a hydrodynamic diameter of approximately 4 nm in the range of renal filtration and has a nearly neutral zeta potential (~+4.0 mV) (**Fig. 4A and 4D**). The conjugation of Axitinib did not significantly alter the size (~4.1 nm) and zeta potential (~+4.3 mV) of Tre-D-Axitinib conjugate. Theoretical molecular weight of Tre-D-Axitinib is 26,556 Da and the MALDI-TOF analysis confirmed the molecular weight of Tre-D-Axitinib. (**Fig 4B and 4D**).

MALDI-TOF analysis of Tre-D-Axitinib (matrix: DHB, linear positive mode) produced a broad molecular ion envelope, which is typical for high-molecular-weight dendrimer-drug conjugates. Such broadness arises from the inherent polydispersity of the conjugation process and the variability in ionization/desorption efficiency for large, heterogeneous molecules in MALDI. These factors can also contribute to a slight apparent underestimation of molecular weight in the spectrum. In our data, the dominant peak cluster centered near ~26.3 kDa is in close agreement with the calculated molecular weight of 26.56 kDa for Tre-D-Axitinib, supporting the expected composition and confirming the successful synthesis of the conjugate. Moreover, the conjugation of Axitinib on dendrimer significantly enhanced the aqueous solubility of Axitinib, which is almost insoluble in water (0.2 µg/mL; **Fig. 4C and 4D**). The solubility of Tre-D-Axitinib based on Axitinib equivalent is ~25mg/mL in water which is almost 125,000 folds more than the free Axitinib.

The shelf stability of the Tre-D-Axitinib formulation was evaluated in PBS at 4°C, and room temperature (25°C) over a 30-day period. Remarkably, the formulation maintained stability throughout this duration, with purities exceeding 99%, as confirmed by HPLC analysis (PDA detector; 331 nm) (**Fig. S47**). Additionally, no shifts in retention time were observed, nor was there any detectable release of Axitinib from the formulation, demonstrating the conjugate's stability under these conditions. We then evaluated the stability of Tre-D-Axitinib at physiological conditions (37 ^o^C, PBS buffer at pH 7.4) over a period of 30 days (**Fig. S48**). No degradation or release of Axitinib was detected, as shown by the HPLC chromatograms (PDA detector; 331 nm) at various time points (**Fig. S47**). This study suggests the structural integrity of the Tre-D-Axitinib conjugate during blood circulation. This non-cleavable conjugate is designed to be effective without the need of Axitinib to be released from the dendrimer. This design prevents premature drug release, reducing the risk of off-target effects and potential toxicity.

### In vitro cellular uptake and VEGFR2 inhibition potential of Tre-D-Axitinib

**Figure 5:**
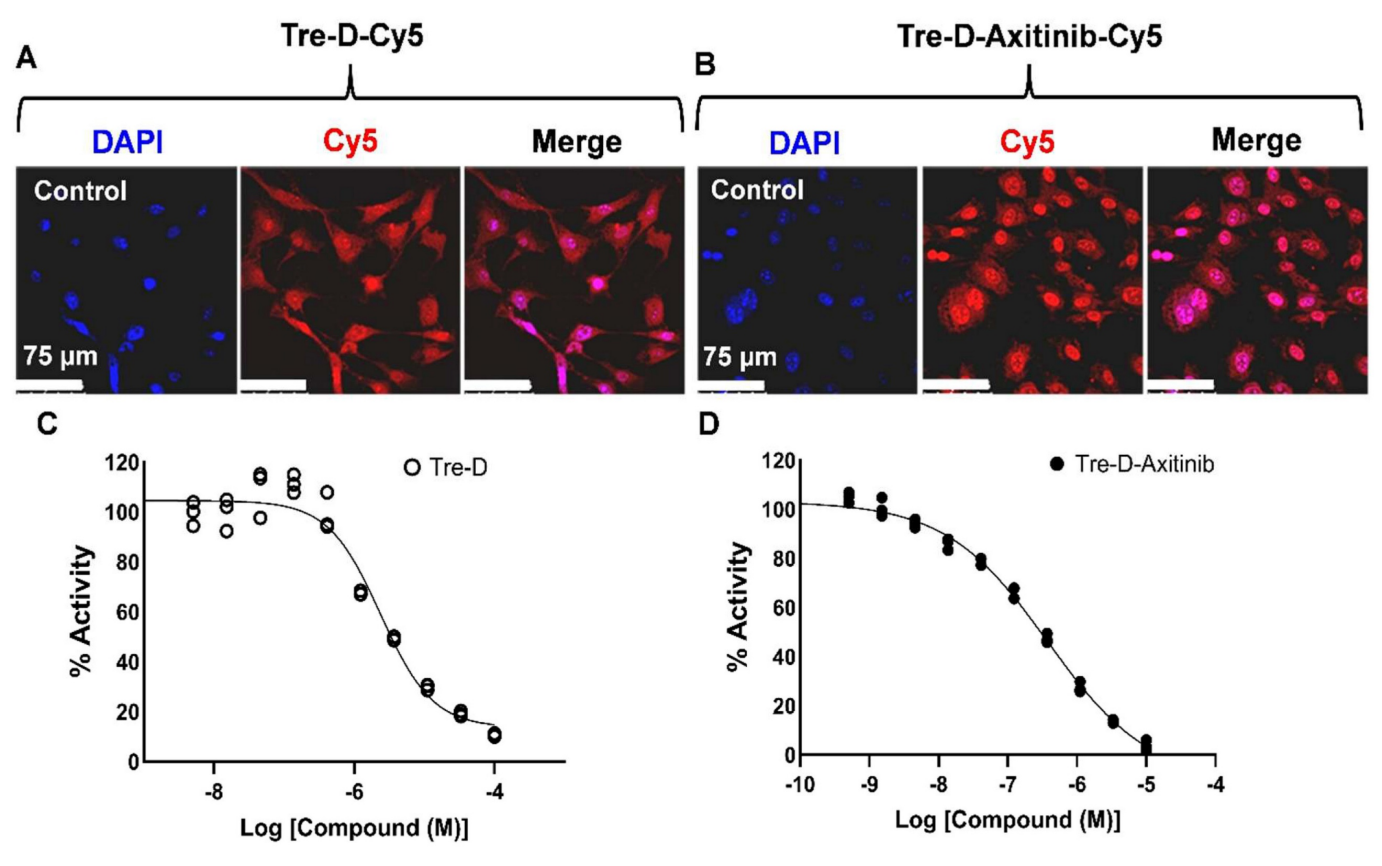
**Cellular uptake and VEGFR2 inhibition potential of Tre-D and Tre-D-Axitinib conjugates.** Confocal micrographs illustrate the uptake of **(A)** Tre-D-Cy5 and **(B)** Tre-D-Axitinib-Cy5 dendrimer in HUVECs, indicated by the red fluorescence of Cy5 within the cells. Nuclei stained with DAPI are shown in blue. The scale bar represents 75 µm. Images are representative of three independent experiments. **(C-D)** IC₅₀ values for VEGFR2 inhibition are shown for **(C)** Tre-D and **(D)** Tre-D-Axitinib.

Before proceeding with the *in vivo* studies, we first assessed the cellular uptake of Tre-D and Tre-D-Axitinib in HUVECs. Fluorescently labeled conjugates, Tre-D-Cy5 and Tre-D-Axitinib-Cy5, were utilized to evaluate the impact of Axitinib conjugation on the uptake of Tre-D in HUVECs. Both Tre-D and Tre-D-Axitinib effectively internalized into the cells, and the conjugation of the hydrophobic Axitinib did not affect the intracellular uptake of Tre-D (**Fig. 5A & 5B**). Subsequently, we examined the VEGFR2 inhibition potential of Tre-D and the Tre-D-Axitinib conjugate. Interestingly, the Tre-D platform itself exhibited some VEGFR2 inhibition activity, with an IC_50_ of approximately 4 µM (**Fig. 5C**) which aligns with literature evidence suggesting that trehalose can inhibit VEGF-induced angiogenesis and vascular proliferation 50. The VEGFR2 inhibition activity of Tre-D-Axitinib was observed to be in nanomolar range (~260 nM; **Fig. 5D**). Considering broad-spectrum inhibition of Axitinib as multiple tyrosine kinases, it is likely that Tre-D-Axitinib exerts its biological effects through a combination of VEGF receptor suppression and interference with other kinase-mediated signaling pathways. Additional studies are warranted to clarify the extent to which each pathway contributes to its overall therapeutic activity.

### Tre-D-Axitinib conjugates reduce angiogenic events in HUVECs

**Figure 6:**
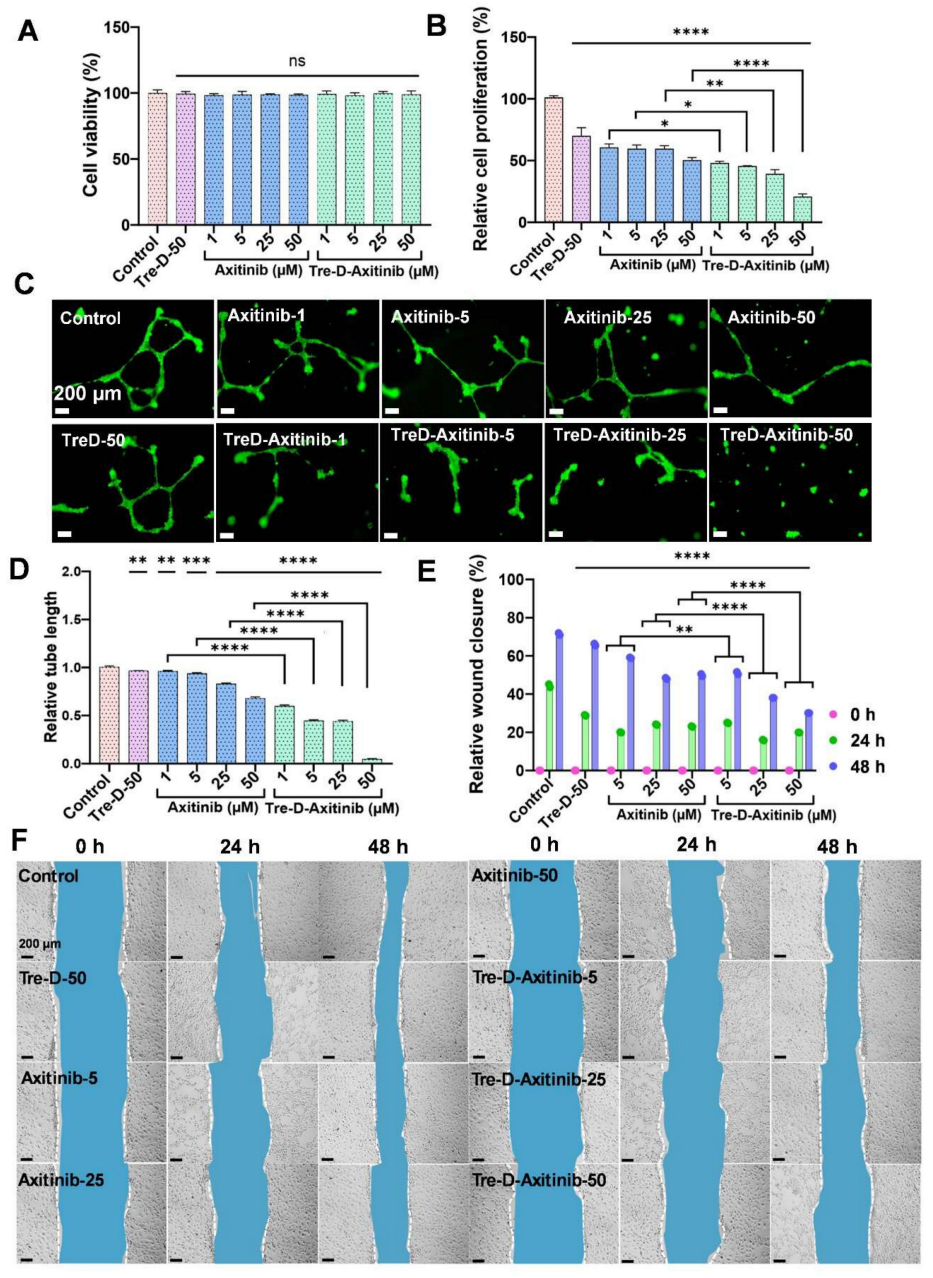
** Effect of Tre-D-Axitinib on angiogenic events and cell migration in HUVECs. (A)** *In vitro* cellular compatibility of HUVECs treated with Tre-D-Axitinib at different concentrations, **(B)** BrdU assay for determining the anti-cell proliferation efficacy of Tre-D-Axitinib on HUVECs. **(C, D)** Microscopy evaluation of anti-angiogenesis effect of Tre-D-Axitinib at differentiation concentrations and corresponding bar graph showing quantitative ImageJ analysis. **(E, F)** Quantitative ImageJ and microscopic evaluation by scratch assay, depicting the *in vitro* anti-migration effect of Tre-D-Axitinib at different concentrations. Data is representation of one of the triplicates, and the scale bar in microscopy images is 200 μm. For cell viability, proliferation and angiogenesis assay, the statistical significance was calculated using ordinary one-way ANOVA, and for cell migration assay, by two-way ANOVA, (****, p < 0.0001; ***, p < 0.001; **, p < 0.01; ns = nonsignificant).

Before proceeding with the efficacy studies, we first evaluated the *in vitro* cellular compatibility of Tre-D control and Tre-D-Axitinib in HUVEC cells (**Fig. 6A**), and RAW macrophages (**Fig. S49**). The results clearly suggest that both control dendrimer (Tre-D) and the Axitinib conjugate (Tre-D-Axitinib) display excellent cytocompatibility at all tested concentrations (1, 5, 25, and 50 µM). Cell proliferation is crucial in the development of proliferative retinopathies, which is characterized by excessive and abnormal retinal vascularization. The increased proliferation leads to the aberrant growth of the blood vessels, resulting in serious visual impairment 58. Previously, it has been shown that Axitinib has potential anti-proliferation effect on the cells by selectively inhibiting VEGF receptor signaling and thereby impairing endothelial cell function and abnormal vascular development 59. Our *in vitro* results indicate that both Axitinib and Tre-D-Axitinib display anti-proliferation effect in a dose dependent manner. In comparison to the untreated control cells (100 % relative cell proliferation), the cell proliferation for free Axitinib and Tre-D-Axitinib at 1, 5, 25 and 50 µM was ~60.7, ~59.7, ~59.6, ~50.5 % and ~48.2, ~45.7, ~39.2, ~20.9 % respectively. The cell proliferation decreased to ~3 and ~5 folds for Axitinib and Tre-D-Axitinib at 50 µM. Moreover, Tre-D-Axitinib is significantly more effective than the free drug (**Fig. 6B**). Interestingly, the Tre-D control (50 µM) also demonstrated anti-proliferation effect to some extent which could be attributed to the VEGFR2 inhibitory potential of Tre-D (**Fig. 6C**). Additionally, upon comparing the anti-proliferative effects of Axitinib and Tre-D-Axitinib, we observed a significant difference in cell proliferation rates. Specifically, Tre-D-Axitinib at concentrations of 1, 5, 25, and 50 μM exhibited approximately ~1.2, ~1.3, ~1.5, and ~2.4-fold greater inhibition of cell proliferation, respectively, compared to the free Axitinib. These findings indicate that Tre-D-Axitinib demonstrates markedly enhanced anti-proliferative activity compared to both free Axitinib and Tre-D.

Next, we evaluated the effect of Tre-D-Axitinib on angiogenesis in HUVECs. It is evident from the fluorescent micrographs that even higher concentrations of free Axitinib (Axi-50) could only decrease the tube length to one-half (less than ~2-fold change) with respect to the control group, as quantified by ImageJ software. However, Tre-D-Axitinib showed a reduction in tube length in a dose dependent manner. Interestingly, the treatment with Tre-D-Axitinib at 50 µM showed a dramatic decrease in tube length (~25 folds) suggesting its potential anti-angiogenic effect on the HUVECs (**Fig. 6C and 6D**). Comparing the efficacy of free Axitinib and Tre-D-Axitinib, the latter exhibited approximately 1.6, 2.1, 1.9, and 13.6-fold reductions in relative tube length at concentrations of 1, 5, 25, and 50 µM, respectively (**Fig. 6C and 6D**). These results indicate that Tre-D-Axitinib possesses superior anti-angiogenic potential in HUVEC cells compared to free Axitinib and naked Tre-D, highlighting its potential as a therapeutic agent in the management of ROP.

Tre-D-Axitinib exhibited a greater therapeutic effect than free Axitinib when treated at the same molar drug concentration. This enhanced efficacy is likely attributable to the dendrimer's multivalent architecture, which presents multiple Axitinib moieties in close spatial proximity, enabling simultaneous engagement with multiple VEGFR2 receptors on the endothelial cell surface. Such multivalent binding (avidity) can enhance receptor clustering, prolong receptor occupancy, and more effectively inhibit VEGFR2-mediated signal transduction, potentially without requiring intracellular release of Axitinib. In this context, cleavage of Axitinib from the Tre-D nanocarrier may not be essential for biological activity. The improved performance of Tre-D-Axitinib at equivalent molar concentrations further supports a multivalent binding mechanism, which could provide a distinct pharmacological advantage over the monovalent binding of free Axitinib.

### Tre-D-Axitinib inhibits cell migration or wound healing in HUVECs

Cell migration is critical in the pathogenesis of proliferative retinopathies, specifically impacting angiogenesis and vascularization within the retina. Abnormal endothelial cell migration is a crucial element in the pathology of proliferative retinopathies, influencing the balance of normal and pathological vascular development 60. The uncontrolled cell migration in proliferative retinopathies leads to the development of unorganized complexes of blood vessels into the vitreous cavity, further causing retinal detachment and vision impairment 61. We next evaluated the impact of Tre-D-Axitinib on cell migration using HUVECs. The qualitative microscopy results suggested a distinct difference between the anti-cell migration effect of the Tre-D control, free Axitinib and the Tre-D-Axitinib conjugate at different time points. At 24 h, as compared to the control group (~44 %), free Axitinib and Tre-D-Axitinib at 5, 25, and 50 µM, the cell migration was found to be ~20, ~24, ~23 % and ~25, ~16, ~20 %, respectively. Furthermore, at 48 h, the cell migration or wound closure rate was observed to be ~71 % (control), and ~59%, ~48%, and ~50% for free drug at tested concentrations of 5, 25, and 50 µg/mL, respectively. Compared to Tre-D, Tre-D-Axitinib at the corresponding concentrations showed reduced migration rates of approximately ~51%, ~38%, and ~30%, respectively. This corresponds to a ~1.2, ~1.3, and ~1.7-fold decrease in cell migration for Tre-D-Axitinib compared to free Axitinib. These data suggest that Tre-D-Axitinib treated HUVECs were less proficient in migrating compared to untreated and Tre-D treated control and free drug (**Fig. 6E and 6F**).

### Qualitative and quantitative biodistribution of systemically administered Tre-D and Tre-D-Axitinib in OIR mouse model

**Figure 7:**
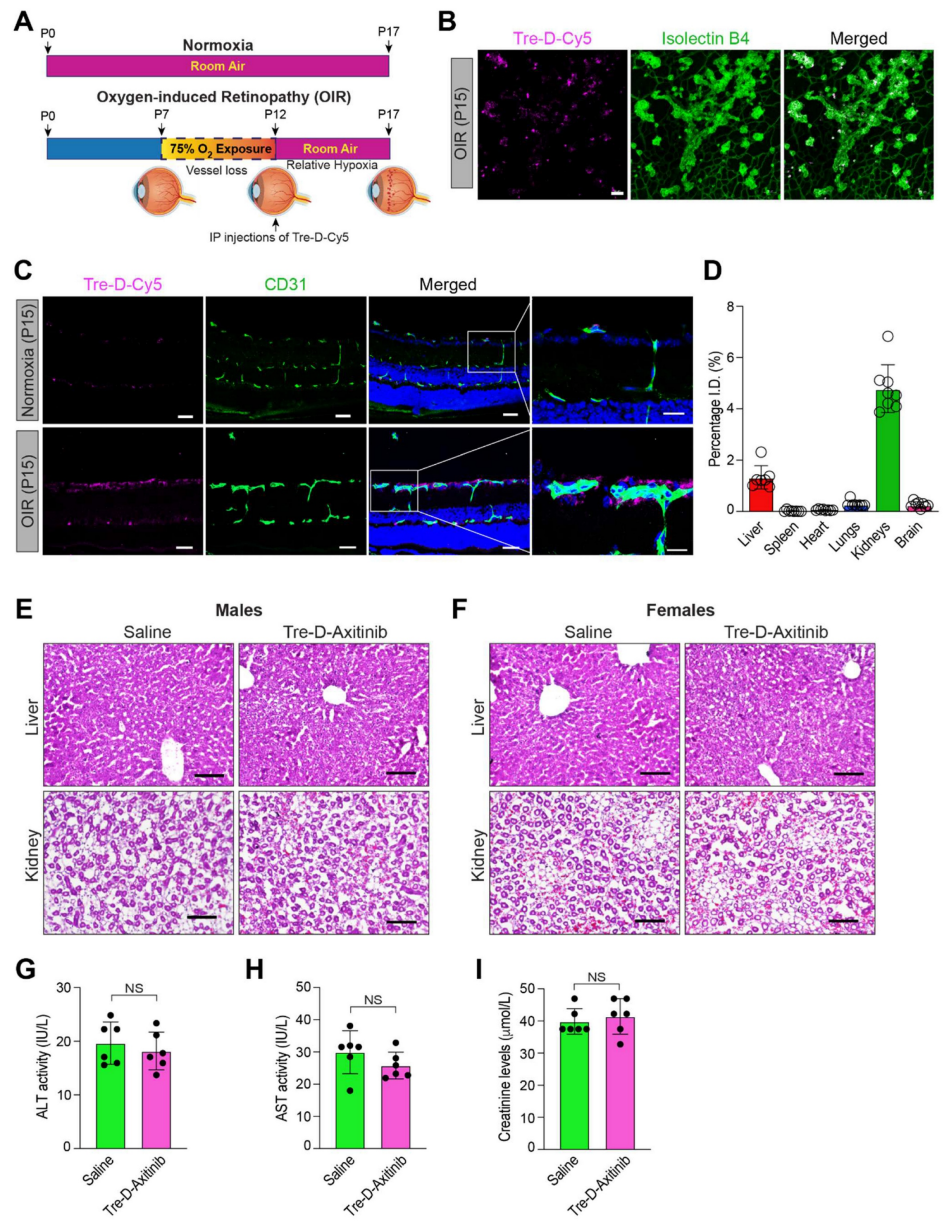
**Retinal localization of Tre-D-Cy5 to neovascular tufts following systemic administration. (A)** Schematic illustrating the mouse OIR model. C57BL/6 mouse pups with their dams were subjected to 75% oxygen from postnatal day 7 to postnatal day 12 and thereafter restored to room air on postnatal day 12. The P12-P17 period represents the neovascular phase. **(B)** C57BL/6 mice pups were exposed to OIR, at P12 the pups were returned to room air, and an IP injection of Tre-D-Cy5 (20 mg/kg Bwt) was given. At P15 eyes were enucleated, retinas isolated, stained with isolectin B4, flat mounts were made and examined for Tre-D-Cy5 in retinal vasculature. **(C)** C57BL/6 mice pups were exposed to normoxia or OIR, administered with Tre-D-Cy5 at P12, and at P15 eyes were enucleated, fixed, and embedded in OCT. The cryosections were prepared and stained with CD-31. **(D)** C57BL/6 mice pups were exposed to OIR. At P12 pups were given IP injection of Tre-D-Axitinib-Cy5 (20 mg/kg Bwt), and at P17 various organs were collected and organ biodistribution of Tre-D-Cy5 was calculated using fluorescence spectroscopy. Since Axitinib alone is not fluorescently labeled, it cannot be detected or quantified using the imaging approach employed in this experiment. Therefore, the inclusion of an Axitinib-alone group would not provide meaningful comparative data in the context of these specific analyses (Fig. 7C & D) and hence was not included as a control in these experiments. **(E, F)** Mice pups were exposed to OIR, and at P12 pups were given an IP injection of PBS (saline) and Tre-D-Axitinib-Cy5 (20 mg/kg Bwt), and at P17 livers and kidneys were collected, fixed, cryosections were made and stained with hematoxylin & eosin (H&E) staining. **(G - I)** The serum ALT activity, AST activity, and creatinine levels were assessed in mouse pups (at P17) that were exposed to OIR and received an IP injection of either PBS or Tre-D-Axitinib-Cy5 at P12. Scale bar represents 50 μm in Fig. B & C, 20 μm in far-right column of Fig. C, and 100 μm in Fig. E & F. NS, non-significant.

Developing systemic therapeutic approaches for retinal diseases is the focus of ongoing research due to the drawbacks associated with intravitreal delivery approaches. To evaluate the significance of Tre-D as a delivery mechanism for retinal targeting when IP administered in proliferative retinopathies, we utilized a mouse model of OIR. In this model, mouse pups are exposed to hyperoxia (75% oxygen) from P7-P12, which induces vessel regression and disrupts normal radial vascular development. Upon returning to room air at P12, the previously avascular retinal regions become hypoxic, triggering the upregulation of angiogenic factors and leading to retinal NV. (**Fig. 7A**). The neovascular phase of this model corresponds to the symptoms of proliferative DR and ROP in humans. We exposed mouse pups to OIR before administering Tre-D-Cy5 intraperitoneally at P12. At P15, we enucleated the eyes, extracted the retina, stained it with isolectin B4, and examined the retinas for Cy5 staining. We observed enhanced accumulation of Cy5 signal in neovascular tufts (**Fig. 7B & 7C**), which suggested that we could use IP injections of Tre-D as a delivery vehicle to target pathological angiogenesis in proliferative retinopathies. We also administered Tre-D-Cy5 to normoxic mice and examined the localization of Tre-D-Cy5 in the retinas of normoxic mice at P15, noting minimal colocalization of Tre-D-Cy5 within the vascular cells (**Fig 7C**). The observed colocalization appears to be confined to the endothelial cells and does not extend outside the vessels in normoxic mice. Before assessing the effect of Tre-D-Axitinib-Cy5 on pathological retinal NV, we evaluated the quantitative distribution of Tre-D-Axitinib-Cy5 in all major organs of OIR-treated mice at P17 following systemic administration at P12. Given that off-target localization and accumulation of nanoparticles poses a significant concern for their clinical application, we thoughtfully designed the Tre-D-Axitinib to be within the size range suitable for renal filtration to prevent unwanted accumulation in major organs. The biodistribution of Tre-D-Axitinib-Cy5 was analyzed in all major organs, including the kidneys, liver, brain, heart, lungs, and spleen (**Fig. 7D**). The animals were perfused with PBS to reduce interference from residual blood and dendrimers retained within the vasculature. The data suggested a minimal off-target uptake that was <1% in all major organs except kidneys which was ~5%. Furthermore, at P17, the liver and kidney sections of the control and Tre-D-Axitinib-treated groups showed no significant damage or difference (**Fig. 7E & 7F**). Additionally, no significant differences were observed in liver and kidney enzyme levels, including Alanine aminotransferase (ALT), aspartate aminotransferase (AST), and creatinine, compared to saline-treated animals **(Fig. G-I)**. These results suggest that the dendrimers are non-toxic to the liver and kidneys in both male and female mice. The combination of targeted retinal uptake and efficient clearance from off-site locations highlights Tre-D as a potential nanoplatform for developing retina-targeted therapies for a range of ocular disorders.

### Systemically administered Tre-D-Axitinib reduces pathological retinal NV in OIR mouse model

**Figure 8:**
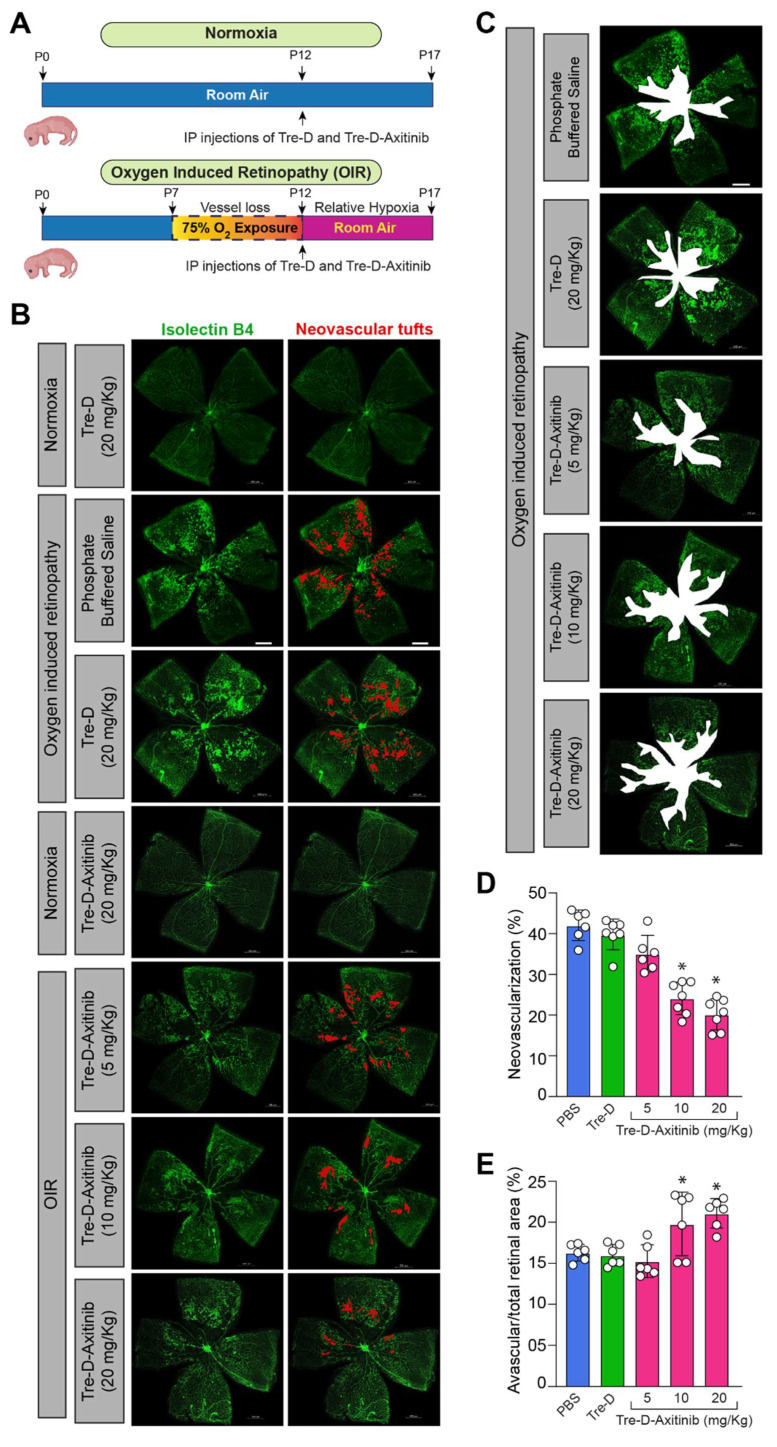
**Tre-D-Axitinib attenuates OIR-induced retinal NV. (A)** Schematic showing the proposed experiments. **(B)** The C57BL/6 mice pups were exposed to OIR and, at P12, administered intraperitoneally with phosphate buffered saline (PBS), or 5, 10, or 20 mg/kg Bwt of Tre-D or Tre-D-Axitinib. At P17 eyes were enucleated, followed by the isolation of retinas. The retinas were then stained with isolectin B4, the flat mounts were prepared and examined for retinal NV. **(C)** The C57BL/6 mice pups were exposed to OIR and, at P12 administered intraperitoneally with PBS or various concentrations of Tre-D or Tre-D-Axitinib. At P17 eyes were enucleated, retinas isolated, stained with isolectin B4, flat mounts were made and examined for avascular area according to methods described by us previously.^48^ **(D & E)** The bar graphs represents NV (%) and avascular/total area (%) of 6 retinas from six individual animals. The values are presented as Mean ± SD. * p < 0.01 vs OIR + Tre-D. Scale bar is 500 μm in panel B, and C.

To find out how Tre-D-Axitinib affects abnormal retinal NV in proliferative retinopathies, we investigated the role of IP injection of Tre-D-Axitinib into normoxic retinas and a mouse model of OIR. We evaluated how it affected neovascular tufts and avascular areas in hypoxic retinas (**Fig. 8A**). We observed that single IP injection of Tre-D-Axitinib at P12, while having no effect on normoxic retinas, significantly reduced OIR-induced retinal NV and showed a reduction in tufts formation or anastomoses in hypoxic retinas when compared to PBS or Tre-D treated animals (**Fig. 8B & 8D**). In addition, we also observed that IP injection of Tre-D-Axitinib resulted in decreased retinal vascularization and led to more widespread area of vaso-obliteration within the ischemic retina (**Fig. 8C & 8E**).

Notably, even though control Tre-D platform demonstrated some VEGFR2 inhibition activity and reduced cell proliferation to some extent *in vitro*, that did not translate *in vivo* at tested concentration. Retinal NV is a clinical symptom of various proliferative retinopathies, including ROP, DR, and the wet form of macular degeneration 5-7. If untreated, retinal NV will ultimately result in visual loss. Intravitreal injections of anti-angiogenic agents are frequently employed to manage these proliferative retinopathies. Intravitreal injections were often associated with endothalmitis, increased intraocular pressure, retinal detachment, vitreous hemorrhage, cataract formation, macular edema, corneal abrasions, and pain or discomfort 62. Recent research shows that diabetic patients who received intravitreal anti-VEGFA injections experienced systemic adverse effects 63. Consequently, ongoing research is being conducted to design therapeutic strategies that can replicate the effects of intravitreal injections via alternative administration routes. These present findings imply that systemic Tre-D-Axitinib administration can be employed to treat ischemic retinopathies, as well as to mitigate the disadvantages of intravitreal injections. These results hold strong promise for developing therapeutic approaches aimed at managing or preventing retinal disorders associated with pathological neovascularization.

### Tre-D-Axitinib attenuates VEGFA-induced angiogenic events in HRMVECs

**Figure 9:**
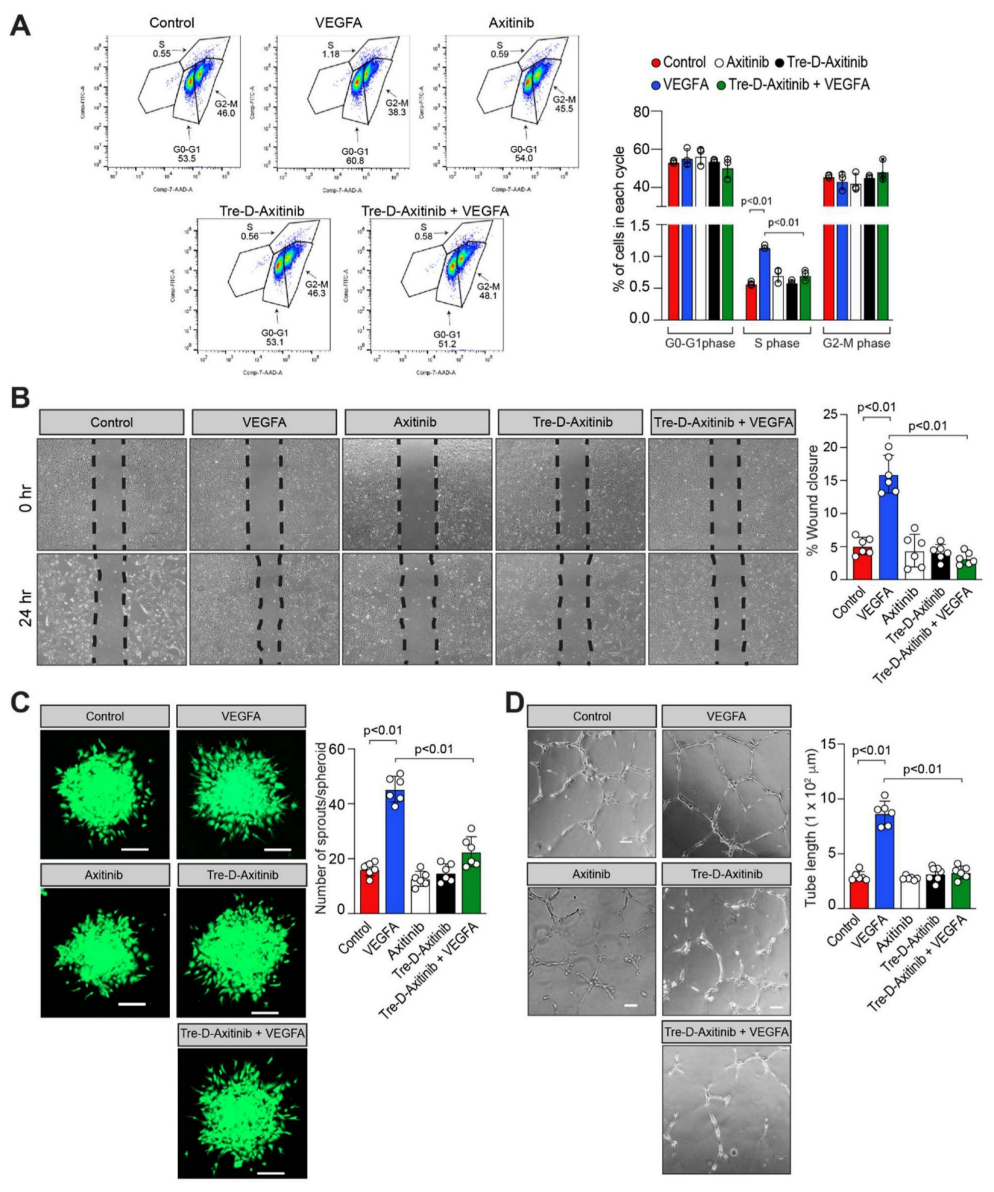
**Tre-D-Axitinib regulates VEGFA-induced angiogenic events in HRMVECs***.* **(A)** HRMVECs were treated with vehicle (PBS), Tre-D (20 ng/ml), or Tre-D-Axitinib (20 ng/ml or or 0.761 nM), followed by the administration of VEGFA at a concentration of 40 ng/mL for 24 h. The proliferation of HRMVECs was assessed using the BrdU proliferation assay. **(B - D)** Quiesced HRMVECs were first treated with Axitinib (50 μM) or Tre-D-Axitinib (20 ng/mL or 0.761 nM) for 30 minutes and thereafter treated with or without VEGFA (40 ng/mL) and subjected to VEGF-induced migration (B), sprouting (C) and tube formation (D) according to methods described by us 48, 49. The bar graphs or dot plots show the quantitative analysis of 3 to 6 independent experiments, expressed as mean ± SD. Scale bar represents 20 μm in figure C and 100 μm in figure D.

OIR is a well-established model in which elevated VEGFA expression has been documented in the retina and anti-VEGFA therapies have been shown to effectively inhibit NV in several diseases. To investigate the functional role of Tre-D-Axitinib in VEGFA-driven angiogenic processes, we examined its effect (20 ng/mL) on VEGFA-induced proliferation, migration, sprouting, and tube formation in HRMVECs. The FITC BrdU assay was employed to assess the impact of Tre-D-Axitinib on VEGFA-induced cell proliferation. As expected, VEGFA treatment significantly increased HRMVEC proliferation compared to vehicle control. However, pretreatment with Tre-D-Axitinib markedly suppressed this VEGFA-induced proliferation, as observed in the Tre-D-Axitinib + VEGFA group (**Fig. 9A**). The effect of Tre-D-Axitinib on VEGFA-induced cell migration was evaluated using a wound healing assay. VEGFA promoted HRMVEC migration, whereas pretreatment with Tre-D-Axitinib significantly inhibited this effect, as shown in the Tre-D-Axitinib + VEGFA group (**Fig. 9B**). The effects of Tre-D-Axitinib on tip cell formation/sprouting and tube formation were evaluated using a 3D angiogenic spheroid assay and a 2D Matrigel assay, respectively. VEGFA stimulation promoted tip cell formation/sprouting and tube formation in HRMVECs, whereas pretreatment with Tre-D-Axitinib significantly reduced these VEGFA-induced angiogenic responses, as observed in the Tre-D-Axitinib + VEGFA group (**Figs. 9C and 9D**). No significant effects of Axitinib or Tre-D-Axitinib alone were observed on HRMVEC proliferation, migration, sprouting, or tube formation (**Figs. 9A-D**). In retinal endothelial cells, VEGFA modulates physiological and pathological angiogenesis in proliferative retinopathies through VEGF receptors activation 64. Previous research indicates that Axitinib inhibits VEGF receptors as well as other tyrosine kinase receptors 65. In our current study, we observed that Tre-D-Axitinib inhibits VEGFA-induced angiogenic signaling, suggesting that its mechanism of action may involve inhibition of VEGF receptor activation. Given the established role of VEGF signaling in promoting retinal angiogenesis 66, and its upregulation in the vasculature of patients with proliferative retinopathies 67, it is plausible that VEGF receptor blockade contributes to the anti-angiogenic effects observed with Tre-D-Axitinib. However, due to Axitinib's known broad-spectrum activity, it remains possible that Tre-D-Axitinib's effects are mediated through a combination of VEGF receptor inhibition and modulation of other tyrosine kinase pathways. Further studies are necessary to delineate the relative contributions of these pathways to the observed therapeutic effects in the OIR model. Furthermore, it has been demonstrated that ischemia and hypoxia (in this case OIR) damage retinal blood vessels and disrupt blood-retinal barrier (BRB) integrity 68. The disruption of BRB integrity enables Tre-D-Axitinib to preferentially accumulate in ischemic retinas, where it subsequently inhibits the activation and downstream signaling of its target receptor tyrosine kinases. This targeted delivery demonstrates the NV-specific therapeutic potential of Tre-D-Axitinib in ischemic retinal tissue. Together, these findings support the potential of Tre-D-Axitinib as a targeted therapeutic for ischemic retinal diseases, although further mechanistic and pharmacokinetic studies are warranted to fully elucidate its mode of action.

## Conclusions

Current interventions for proliferative retinopathies, while effective, face substantial limitations. Intravitreal anti-VEGFA therapies are invasive, require frequent administration, and are associated with ocular complications and systemic adverse effects. Additionally, these therapies often fail to achieve selective targeting of neovascular tufts, limiting their therapeutic efficacy. No organic nanoparticles have yet demonstrated the ability to specifically localize within aberrant neovascular tufts at retinal pathology sites after systemic delivery. Tre-D-Axitinib addresses these gaps by providing a systemic, minimally invasive alternative that combines the selective targeting capabilities of Tre-D with the potent anti-angiogenic effects of Axitinib. Tre-D is designed to have inherent NV targeting abilities without the need of any additional targeting ligands, limiting post-synthetic modifications. Our findings demonstrate that Tre-D-Axitinib significantly reduces pathological NV while leading to increased vaso-obliteration in the ischemic retina. These findings suggest that Tre-D-Axitinib, in addition to reducing pathological retinal NV, may also affect vascular repair, which needs further investigation. The high aqueous solubility, biocompatibility, and scalable synthesis of Tre-D further enhance its clinical viability, presenting a robust alternative to existing intravitreal therapies. The development of Tre-D-Axitinib represents a significant step forward in nanomedicine, offering a transformative solution to the challenges associated with the current treatment of ischemic retinopathies.

## Supplementary Material

Supplementary figures.

## Abbreviations

CuAAC: Copper-catalyzed Azide-Alkyne Cycloaddition
DMF: *N*, *N*-dimethylformamide
EDC: 1-Ethyl-3-(3-dimethylaminopropyl)carbodiimide
G1: generation-1
HOBt: Hydroxybenzotriazole
HRMECs: Human Retinal Microvascular Endothelial Cells
HUVECs: Human umbilical vein endothelial cells
IP: Intraperitoneal
OIR: Oxygen Induced Retinopathy
RT: room temperature
THF: Tetrahydrofuran
MW: microwave
NMR: Nuclear Magnetic Resonance
NV: Neovascularization
PEG: polyethylene glycol
OEG: oligoethylene glycol
