# Supplementary material for: Systemic targeting of aberrant neovascular tufts using trehalose-dendrimer nanocarriers for the treatment of proliferative retinopathies
Source: Theranostics. 2026 Jan 1;16(3):1504–27. doi: 10.7150/thno.120357 (PMC12679583; doi:10.7150/thno.120357)
Supplement: Supplementary file 1 — Supplementary figures. [file thnov16p1504s1.pdf]

Supplementary Information

Systemic targeting of aberrant neovascular tufts using trehalose-dendrimer nanocarriers for the treatment of proliferative retinopathies

Anu Rani,[1#] Shivantika Bisen,[2, 3#] Rishi Sharma,[1#] Aqib Iqbal Dar,[1] Anamika Sharma,[2, 3] Anubhav Dhull,[1] Nina Palmer,[1] Kenneth John Goody,[1] Nikhlesh K Singh,[2, 3]* Anjali Sharma[1]*

[1]*Department of Chemistry, College of Arts and Sciences, Washington State University, 1470 NE College Ave, Pullman, WA, USA 99164.*

[2]*Integrative Biosciences Center, Wayne State University, Detroit, MI, 48202, USA*

[3]*Department of Ophthalmology, Visual and Anatomical Sciences, School of Medicine, Wayne State University, Detroit, MI, 48202, USA*

*Correspondence:

Anjali Sharma, Department of Chemistry, Washington State University, Troy Hall, Rm 222, 1470 E. College Avenue, Pullman, WA 99164, USA. E-mail: anjali.sharma@wsu.edu

Nikhlesh K. Singh, Department of Ophthalmology, Visual and Anatomical Sciences, School of Medicine, Wayne State University, Detroit, MI, 48202, USA. Email: nsingh2@wayne.edu

[#]These authors contributed equally.

Contents:

1. Chemistry-Experimental Section
   - 1.1 Materials & Reagents
   - 1.2 Detailed Instrumentation & methods
   - 1.3 Synthetic procedures & Methods
   - 1.4 Stability studies
2. Supporting Figures

1. Chemistry-Experimental section

1.1 Material and reagents

The chemicals, solvents and all the reagents were ordered from Thermo Scientific, Ambeed, Sigma Aldrich. Analytical-grade reagents and HPLC grade solvents were used for the synthesis of building blocks. For the reaction tracking we used silica gel 60 F254 plates (aluminum foil), with spot visualization achieved *via* UV light or staining reagents. Compounds were isolated and purified through flash column chromatography on silica gel 60 (230–400 mesh). For the purification of dendrimers, we used Spectra/Por dialysis membranes from Repligen.

1.2 Instruments

^1H and ^{13}C NMR spectra were recorded on a Bruker 500 MHz high-resolution NMR spectrometer. Samples were prepared in deuterated solvents such as DMSO-d_6, $CDCl_3$, or D_2O. Chemical shifts (δ) are reported in parts per million (ppm) relative to residual solvent peaks and coupling constants (J) are given in hertz (Hz). The following abbreviations denote multiplicities: s = singlet, d = doublet, t = triplet, q = quartet, and m = multiplet. Microwave-assisted reactions were performed using a Biotage Initiator+ system in sealed microwave vials. High-resolution mass spectrometry (HRMS) analyses for small molecules were carried out by Dr. Yue Li (Department of Chemistry and Biochemistry, University of Maryland–College Park) using electrospray ionization (ESI) on a Bruker MALDI-TOF or Q-TOF instrument, with trans-2-[3-(4-tert-butylphenyl)-2-methyl-2-propenylidene]malononitrile as the matrix.

Particle size and surface charge of dendrimer–drug conjugates were determined by Dynamic Light Scattering (DLS) and zeta potential measurements using a Malvern Zetasizer Nano 90 (Westborough, MA) at 25 °C. For DLS, conjugates were dissolved in deionized water (18.2 MΩ cm) at 0.5 mg/mL, filtered through 0.2 μm syringe filters (Pall Corporation, HT Tuffryn membrane), and transferred to UV-transparent disposable cuvettes (12.5 × 12.5 × 45 mm). Zeta potential measurements were performed on 0.2 mg/mL samples in 10 mM NaCl using identical filtration and analyzed with a Malvern Zetasizer Nanoseries capillary cell.

The purity and stability of small molecules, dendrimers, and dendrimer–drug conjugates were analyzed by high-performance liquid chromatography (HPLC) using a Waters Acquity Arc system (Milford, MA, USA) equipped with binary pumps, a 2998 PDA detector, and a 2475 fluorescence detector. Chromatographic data was processed with Waters Empower software. Separation was achieved on a Waters Symmetry C18 column (300 Å, 5 μm, 4.6 × 250 mm) using a gradient elution from 90:10 to 40:60 (A:B), where solvent A was 0.1% TFA + 5% ACN in water and solvent B was 0.1% TFA in ACN. The gradient increased to 40:60 (A:B) at 15

min, held constant to 30 min, then returned to 90:10 (A:B) at 35 min. The flow rate was maintained at 1 mL/min. Dendrimers and their conjugates were monitored at 210 nm and 254 nm, while Cy5-labeled conjugates were also analyzed at 650 nm.

1.3 Synthetic Procedures

Synthesis of compound 2: Compound **1** (2 g, 1.0 eq, 2.81 mmol) was dissolved in anhydrous DMF (20 mL). To the stirring solution, sodium hydride (505 mg, 4.5 eq, 12.64 mmol, 60% dispersion in mineral oil) was slowly added in portions at 0°C. The solution was stirred for 15 min at 0 °C. This was followed by the addition of propargyl bromide (1.06 mL, 4.0 eq, 11.22 mmol, 80% w/w solution in toluene) at 0°C, and the stirring continued at room temperature for another 3 h. On completion, the reaction was quenched by the addition of saturated ammonium chloride solution and extracted with ethyl acetate (2 × 150 ml). The combined organic layer was washed with chilled brine (2 × 100 ml), dried over Na_2SO_4, and evaporated in vacuo. The crude product was purified by silica flash column chromatography (0-5% MeOH/CH_2Cl_2) to afford 1.7 g of compound **2** (2.06 mmol, 73.3%) as a brown viscous liquid.

^{1}H NMR (500 MHz, $CDCl_3$) δ 7.28 (s, 2H), 4.17-4.25 (m, 12H), 3.84-3.90 (m, 7H), 3.79 (t, J = 5.2 Hz, 3H), 3.63-3.73 (m, 35H), and 2.43 (s, 3H). ^{13}C NMR (125 MHz, DMSO-d_6) δ 166.3, 152.5, 142.3, 124.8, 108.5, 80.8, 77.6, 77.5, 72.8, 72.4, 70.5, 70.4, 70.3, 70.2, 70.1, 70.0, 69.4, 69.0, 68.9, 60.6, 57.94, 52.6. MS (ESI) *m/z*: calculated for $C_{41}H_{62}O_{17}$ $[M+Na]^+$: 849.3885, found 849.3870.

Synthesis of compound 3: To a stirring solution of compound **2** (1 g, 1.0 eq, 1.21 mmol) in THF (10 mL) was added a solution of LiOH.monohydrate (290 mg, 10 eq, 12.10 mmol) in DI water (4 mL) and the reaction mixture was stirred for 24 h at room temperature. The reaction was monitored with thin-layer chromatography (TLC) and the reaction mixture was diluted with water (150 mL), followed by the addition of saturated citric acid solution to adjust the pH in between 3-4 and extracted with DCM (2 × 100 mL). The combined organic layer was washed brine (50 mL), then dried using anhydrous Na_2SO_4, filtered, and evaporated in vacuo. The crude product was purified by silica flash column chromatography (0-10% MeOH/CH_2Cl_2) to afford 780 mg of compound **3** (0.96 mmol, 79.3 %) as a brown viscous liquid.

^{1}H NMR (500 MHz, $CDCl_3$) δ 7.14 (s, 2H), 4.05–4.14 (m, 12H), 3.73 (t, J = 5.1 Hz, 4H), 3.67 (t, J = 5.1 Hz, 3H), 3.49–3.59 (m, 35H), and 2.31 (m, 3H). ^{13}C NMR (125 MHz, $CDCl_3$) δ 169.9, 152.3, 143.2, 124.2, 109.7, 79.7, 79.6, 74.7, 74.6, 72.5, 70.9, 70.8, 70.7, 70.6,

70.6S, 70.5, 70.4, 70.3, 69.7, 69.1, 68.9, 58.4, 58.3. ESI-MS *m/z*: calculated for $C_{40}H_{60}O_{17}$ [M-H]$^+$: 811.3752, found 811.3752.

Synthesis of (2R,3R,4S,5R,6R)-2-(acetoxymethyl)-6-(((2R,3R,4S,5R,6S)-3,4,5-triacetoxy-6-(azidomethyl)tetrahydro-2H-pyran-2-yl)oxy)tetrahydro-2H-pyran-3,4,5-triyl triacetate (4) : The compound (2R,3R,4S,5R,6R)-2-(acetoxymethyl)-6-(((2R,3R,4S,5S,6R)-3,4,5-triacetoxy-6-(bromomethyl)tetrahydro-2H-pyran-2-yl)oxy)tetrahydro-2H-pyran-3,4,5-triyl triacetate (18.66 g, 26.63 mmol) was dissolved in dry DMF (280 mL) and stirred under a nitrogen environment. Solid sodium azide (8.69 g, 133.63 mmol) was added to the reaction mixture, and the solution was heated at 55°C and stirred for 16 h. At completion of reaction, the solvent was removed under reduced pressure and the crude was dissolved in EtOAc (500 mL). The organic layer was washed 3 x 150mL with water and (3 x 100 mL) brine. The organic layer was dried with anhydrous Na_2SO_4 and concentrated under reduced pressure. The crude brown-solid was purified by column chromatography on silica gel (0-70% EtOAc/Hexane) to afford 12.20 g of **4** (18.44 mmol, 69.1%) as a white solid.

^{1}H NMR (500 MHz, DMSO-d$_6$) δ 5.20–5.38 (m, 4H), 4.97–5.09 (m, 4H), 4.16–4.23 (m, 1H), 3.94–4.06 (m, 3H), 3.47–3.54 (m, 1H), 3.39–3.46 (m, 1H), and 1.98–2.10 (m, 21H). ^{13}C NMR (125 MHz, DMSO-d$_6$) δ 170.5, 170.2, 170.0, 169.8, 92.5, 92.4, 79.6, 70.0, 69.6, 69.5, 69.1, 68.5, 68.4, 62.2, 50.3, 40.4, 40.2, 40.1, 20.9, 20.9, 20.8, 20.8.

Synthesis of compound 5: A stirred solution of compound **3** (614 mg, 1.0 eq, 0.75 mmol) in DMF (5 mL) was placed in a microwave vial, followed by the addition of compound 4 (1.7 g, 3.5 eq, 2.57 mmol) dissolved in DMF (5 mL). To this mixture, an aqueous solution of $CuSO_4{\cdot}5H_2O$ (5 mol% per alkyne, 1 mL DI water) was added, and after 2 minutes of stirring, a freshly prepared sodium ascorbate solution (10 mol% per alkyne in 0.5 mL DI water) was introduced. The reaction was then subjected to microwave irradiation at 50 °C for 12 h. Reaction progress was monitored by thin-layer chromatography (TLC). Upon completion, the mixture was diluted with dichloromethane (100 mL), washed sequentially with saturated EDTA solution (7 × 30 mL) and brine (1 × 50 mL), dried over anhydrous Na_2SO_4, filtered, and concentrated under reduced pressure. The crude product was purified by silica flash column chromatography (0-10% MeOH/CH_2Cl_2) to afford 1.87 g of compound **5** (0.68 mmol, 90%) as a white solid.

^{1}H NMR (500 MHz, DMSO-d$_6$) δ 8.00 (s, 3H), 7.21 (s, 2H), 5.28 (m, 10H), 5.01 (m, 14H), 4.84 (m, 3H), 4.65 (d, J = 14.4 Hz, 3H), 4.50 (m, 10H), 4.17 (m, 9H), 4.10 (m, 6H), 3.98 (m, 8H), 3.75 (m, 10H), 3.66 (t, J = 4.8 Hz, 2H), 3.60 (m, 4H), 3.53 (m, 29H), 2.05 (m, 20H), and 1.98 (m, 38H). ^{13}C NMR (125 MHz, $CDCl_3$) δ 170.7, 170.6, 170.0, 169.9, 169.8, 169.7,

169.6, 169.5, 169.4, 162.6, 145.5, 92.2, 91.5, 72.3, 70.9, 70.8, 70.7, 70.6, 70.5, 70.4, 70.0, 69.9, 69.8, 69.7, 68.9, 68.5, 68.3, 61.7, 36.5, 31.4, 20.9, 20.8, 20.7, 20.6, 20.5. ESI-MS *m/z*: calculated for $C_{120}H_{167}N_9O_{70}$ $[M-H]^+$: 2795.97, found 2794.97.

Synthesis of compound 7: In a flame-dried 50 mL round-bottom flask, compound **5** (200 mg, 1.0 eq, 0.08 mmol) was dissolved in 4 mL of anhydrous DCM under an argon atmosphere and cooled to 0 °C. To the stirred solution, EDC·HCl (22.5 mg, 1.5 eq, 0.12 mmol) and HOBt (16.7 mg, 1.4 eq, 0.11 mmol) were added, and the reaction mixture was stirred for 30 min at 0 °C. Compound **6** (47.6 mg, 2.0 eq, 0.16 mmol) dissolved in 1 mL of dry DCM was then added dropwise. The mixture was allowed to warm to room temperature and stirred for an additional 1.5 h. Reaction progress was monitored by TLC under UV light. Upon completion, the mixture was diluted with DCM and the organic layer was washed successively with water (5 × 30 mL) and brine (2 × 30 mL), dried over anhydrous Na_2SO_4, filtered, and concentrated under reduced pressure The crude product was purified by silica flash column chromatography (0-10% MeOH/CH_2Cl_2) to afford 178 mg of compound **7** (0.056 mmol, 80.6%) as a white solid.

^{1}H NMR (500 MHz, DMSO-d_6) δ 8.46 (t, 1H), 8.00 (s, 3H), 7.18 (s, 2H), 5.27 (m, 10H), 5.00 (m, 13H), 4.84 (d, J = 3.6 Hz, 3H), 4.65 (dd, J = 14.5, 2.9 Hz, 4H), 4.50 (d, J = 16.9 Hz, 10H), 4.16 (m, 12H), 3.98 (m, 10H), 3.75 (m, 6H), 3.66 (t, J = 5.0 Hz, 2H), 3.51 (m, 53H), and 1.97 (m, 63H). ^{13}C NMR (125 MHz, $CDCl_3$) 175.2, 175.0, 174.9, 174.7, 174.6, 174.5, 170.7, 156.9, 149.2, 145.1, 134.5, 130.2, 111.5, 96.5, 96.2, 77.0, 75.2, 75.1, 75.0, 74.9, 74.8, 74.7, 74.6, 74.4, 74.3, 74.2, 74.1, 74.0, 73.8, 73.5, 73.4, 73.2, 73.1, 68.5, 66.9, 55.2, 54.8, 25.7, 25.6, 25.5, 25.5, 25.4.

Synthesis of compound 8: In a flame-dried round-bottom flask, compound **7** (1.0 g, 1.0 eq, 0.32 mmol) was dissolved in 10 mL of dry methanol under stirring. To this solution, sodium methoxide (300 μL, 1.1 M in MeOH) was added dropwise until the pH reached approximately 8.5-9. The reaction mixture was stirred at room temperature for 16 h. Progress of the reaction was monitored by TLC. Upon completion, the pH was adjusted to 6-7 using Amberlite IR-120 resin, and the mixture was filtered to remove the resin. The resin was first removed by filtration, and the solvent was removed by rotary evaporation to yield 655 mg of compound **8** (0.296 mmol, 92.5 %) as a brown viscous liquid.

^{1}H NMR (500 MHz, D_2O) δ 8.05 (m, 3H), 7.16 (m, 2H), 5.09 (m, 3H), 4.65 (m, 12H), 4.26 (m, 4H), 4.11 (m, 4H), 3.93 (m, 4H), 3.74 (m, 90H), and 3.35 (m, 6H). ^{13}C NMR (125 MHz, D_2O) δ 169.3, 151.9, 143.7, 139.6, 126.0, 106.3, 93.3, 93.1, 72.5, 72.3, 72.1, 70.9, 70.8, 70.2, 70.1, 69.9, 69.8, 69.7, 69.6, 69.5, 69.4, 69.1, 68.8, 68.3, 62.9, 60.4, 50.7, 50.0, 39.6.

Synthesis of compound 9: The methanolic solution of the G1 PAMAM dendrimer was first evaporated, and residual methanol was removed under reduced pressure by co-evaporation with 1 mL of DMF. The dried PAMAM dendrimer (1.1 g, 1.0 eq, 0.77 mmol) was then dissolved in 5 mL of anhydrous DMF under a nitrogen atmosphere. In a separate vial, 5-hexynoic acid (2.0 g, 24 eq, 18.48 mmol) was dissolved in 5 mL of anhydrous DMF and activated by the addition of EDC (3.5 g, 24 eq, 18.48 mmol). The mixture was stirred for 15 min to allow activation, after which the activated solution was added dropwise to the dendrimer solution under continuous stirring. Following complete addition, DMAP (1.1 g, 12 eq, 9.24 mmol) was introduced to the reaction mixture, which was then stirred at room temperature for 24 h. Upon completion, the reaction mixture was purified by dialysis using a 1 kDa MWCO membrane against 20% DMF in water for 12 h. The last runs of dialysis were performed against DI water to remove the traces of DMF. The resulting aqueous solution was lyophilized to afford 1.4 g of compound **9** (0.64 mmol, 83%) as a brown amorphous solid.

^{1}H NMR (500 MHz, DMSO-d_6) δ 8.06 – 7.74 (m, 20H), 3.19 – 2.99 (m, 40H), 2.77 (t, J = 2.7 Hz, 8H), 2.65 (t, J = 7.3 Hz, 24H), 2.42 (t, J = 6.7 Hz, 12H), 2.28 – 2.08 (m, 55H), 1.65 (p, J = 7.3 Hz, 16H). ^{13}C NMR (125 MHz, DMSO-d_6) δ 172.1, 172.0, 171.7, 84.5, 71.9, 55.4, 52.6, 50.1, 50.0, 38.8, 38.7, 37.4, 34.6, 33.7, 24.6, 17.9.

Synthesis of compound 10: Compound **9** (7.0 mg, 1.0 eq, 0.0032 mmol) was dissolved in 1 mL of DMF and transferred to a 5 mL microwave vial. To this solution, compound **7** (100 mg, 10.0 eq, 0.033 mmol) dissolved in 2 mL of DMF was added. Subsequently, $CuSO_4 \cdot 5H_2O$ (5 mol% per alkyne, in 0.2 mL of water) was introduced dropwise, followed by the addition of sodium ascorbate (10 mol% per alkyne). The reaction mixture was subjected to microwave irradiation at 40 °C for 15 h. Reaction progress and product formation were confirmed by HPLC analysis. Upon completion, the mixture was purified by dialysis using a 3.5 kDa MWCO membrane against 20% DMF in water for 12 h, followed by dialysis against deionized water to remove residual DMF. The purified product was lyophilized to afford 75 mg of acetylated-trehalose (**10**) dendrimer (0.0027 mmol, 84%) as a brown solid.

^{1}H NMR (500 MHz, DMSO-d_6) δ 8.41 (t, J = 5.8 Hz, Tre-D-amides), 7.94 (m, Tre-D-amides + triazole H), 7.85 (m, 3H), 7.75 (s, 4H), 7.11 (s, 16H, Tre-D-Ar-H), 5.21 (m, sugar-H), 4.94 (m, sugar-H), 4.78 (d, J = 4.0 Hz, sugar-H), 4.58 (dd, J = 14.5, 2.9 Hz, sugar-H), 4.41 (m, sugar-H), 4.10 (m, sugar-H), 3.96 (m, sugar-H + PEG-H), 3.87 (m, sugar-H + PEG-H), 3.69 (m, sugar-H + core + PEG), 3.59 (t, J = 5.0 Hz, sugar-H + core + PEG), 3.51 (m, sugar-H + core), 3.43 (m, PEG-H), 3.00 (s, core-H), 1.91 (m, acetates), 1.17 (s, sugar-H). ^{13}C NMR (125 MHz, DMSO-d_6) δ 170.6, 170.2, 170.0, 152.2, 124.7, 106.7, 96.6, 72.6, 70.5, 70.4, 70.3,

70.2, 70.13, 70.1, 70.0, 69.8, 69.4, 69.3, 69.1, 68.9, 68.8, 67.7, 66.6, 63.9, 62.5, 49.7, 34.9, 21.1, 21.0, 20.9

Synthesis of compound 11:

Route 1: In a flame-dried round-bottom flask, acetylated trehalose dendrimer **10** (1.0 g, 1.0 eq, 0.32 mmol) was dissolved in 10 mL of dry methanol under stirring. Sodium methoxide (300 µL, 1.1 M in MeOH) was added dropwise until the pH reached approximately 8.5–9. The reaction mixture was stirred at room temperature overnight, and progress was monitored by TLC. Upon completion, the pH was adjusted to 6–7 using Amberlite IR-120 resin. The resin was removed by filtration through a cotton plug, and the solvent was evaporated under reduced pressure to afford trehalose dendrimer **11** in 90% yield.

Route 2: In a 10 mL microwave vial, compound **9** (26.6 mg, 1.0 eq, 0.012 mmol) was dissolved in 2 mL of DMF. To this solution, compound **8** (230 mg, 10.0 eq, 0.12 mmol) dissolved in 3 mL of deionized water was added. Subsequently, aqueous solutions of $CuSO_4·5H_2O$ (5 mol% per alkyne) and sodium ascorbate (10 mol% per alkyne) were introduced sequentially. The reaction mixture was subjected to microwave irradiation at 80 °C for 15 h. Reaction progress and product formation were confirmed by HPLC analysis. Upon completion, purification was performed by TFF using a 3 kDa MWCO membrane, initially with 20% DMF in water, followed by dialysis against deionized water. The aqueous solution was lyophilized to afford 205 mg of compound **11** (0.0104 mmol, 86%) as a brown amorphous solid.

^{1}H NMR (500 MHz, DMSO-d_6) δ 8.48 (t, *J* = 5.8 Hz, 8H, Tre-D-amides), 8.09 – 7.75 (m, 54H, Tre-D-core-amides + triazole H), 7.18 (s, 16H, D-Ar-H), 5.32 (s, sugar-H), 4.97 (s, sugar-H), 4.87 – 4.68 (m, sugar-H), 4.67 – 4.25 (m, sugar-H), 4.21 – 3.98 (m, PEG-H + sugar-H), 3.91 – 3.72 (m, PEG-H), 3.71 – 3.52 (m, PEG-H), 3.51 – 3.26 (m, PEG-H + core-H), 3.26 – 3.14 (m, core-H), 3.13 – 3.02 (m, core-H), 2.98 (t, *J* = 9.3 Hz, Core-H), 2.82 – 2.52 (m, core-H), 2.34 – 2.02 (m, 42H, sugar-H), 1.84 – 1.75 (m, 16H, sugar-H).

^{1}H NMR (500 MHz, D_2O) δ 7.89 (m, Tre-d-triazoles), 7.06 (s, 16H, Tre-D-Ar), 5.02 (t, J = 3.3 Hz, sugar-H), 4.55 (m, PEG-H), 3.58 (m, PEG-H), 2.35 (m, sugar-H), 1.80 (m, sugar-H), and 1.22 (s, sugar-H). ^{13}C NMR (125 MHz, DMSO-d_6) δ 166.0, 152.2, 144.1, 140.3, 129.7, 124.9, 124.7, 122.4, 106.7, 93.7, 73.2, 73.1, 72.3, 71.9, 71.8, 70.5, 70.4, 70.3, 70.2, 70.1, 69.4, 69.2, 68.8, 63.8, 61.2, 51.2, 49.6, 49.1, and 29.5.

Synthesis of compound 12: To activate, 5-hexynoic acid (85 mg, 15.0 eq, 0.76 mmol) was dissolved in anhydrous DMF (10mL) and treated with EDC·HCl (238.8 mg, 25.0 eq, 1.25 mmol). The reaction mixture was stirred for 10-15 minutes. Next, a solution of Trehalose dendrimer **11** (1.0 g, 1.0 eq, 0.05 mmol) in DMF (5 mL) was added dropwise to the stirred

activated hexynoic acid solution. Following this, DMAP (43 mg, 7.0 eq, 0.35 mmol) was added to the reaction mixture. The entire reaction was allowed to stir at room temperature for 24 h. The completion of the reaction was confirmed by HPLC analysis, as indicated by a shift in the retention time relative to the starting material. The crude dendrimer was purified by dialysis using a 1 kDa MWCO membrane, first against 20% DMF in water, followed by dialysis in deionized water with frequent solvent exchange to remove residual DMF. The product was lyophilized to afford 940 mg of compound **12** (0.45 mmol, 90%) as a brown amorphous solid.

^{1}H NMR (500 MHz, DMSO-d$_6$) δ 8.48 (s, 8H, Tre-D-amides), 8.00 – 7.79 (m, 54H, Tre-D-core-amides + triazole H), 7.18 (s, 16H, D-Ar-H), 5.43 – 4.91 (m, sugar-H), 4.91 – 4.65 (m, sugar-H), 4.69 – 4.28 (m, sugar-H), 4.28 – 3.88 (m, PEG-H), 3.87 – 3.41 (m, PEG-H+ core-H), 3.30 – 2.91 (m, PEG-H+ core-H), 2.87 – 2.75 (m, core-H), 2.71 – 2.53 (m, sugar-H + core-H), 2.48 – 2.29 (m, sugar-H + linker-H), 2.28 – 2.06 (m, sugar-H + linker-H), 1.82 – 1.65 (m, 42H, sugar-H + linker-H).

Synthesis of Tre-D-Cy5 (13): In a 5 mL round-bottom flask, compound **12** (25 mg, 1.0 eq, 0.0012 mmol) was dissolved in 1 mL of deionized (DI) water. A solution of Cy5-azide (3.1 mg, 3.0 eq, 0.0037 mmol) in 1 mL of DMF was then added. To this mixture, $CuSO_4 \cdot 5H_2O$ (10 mol% per alkyne, in 0.1 mL water) and sodium ascorbate (15 mol% per alkyne, in 0.1 mL water) were added sequentially. The reaction was stirred at 40 °C for 10 h. Upon completion, the mixture was purified by dialysis (1 kDa MWCO, against DI water for 12 h) and lyophilized to yield 24 mg of compound **13** (0.0011 mmol, 89%) as a blue solid.

^{1}H NMR (500 MHz, DMSO-d$_6$) δ 8.40 (s, 2H, Cy5-Ar), 8.30 (s, 4H, Cy5-Ar), 7.91 (s, 20H, Tre-D amides and triazoles + Cy5-Ar), 7.86 – 7.69 (m, 35H, Tre-D-core-amides), 7.64 – 7.53 (m, 12H, Tre-D + Cy5-Ar), 7.42 (d, *J*=8.1, 10H, Tre-D + Cy5-Ar), 7.31 – 7.22 (m, 12H, Cy5), 7.11 (s, 16H, Tre-D-Ar), 6.55 – 6.48 (m, Cy5-H), 6.24 (d, *J*=13.9, Cy5), 5.24-5.23 (m, sugar-H), 4.89 (s, sugar-H), 4.78 – 4.67 (m, core + sugar-H), 4.57 – 4.37 (m, core + sugar-H), 4.29 (s, core + sugar-H), 4.17 – 3.94 (m, sugar-H), 3.64 (d, *J*=46.8, PEG-H), 3.57 – 3.38 (m, PEG-H), 3.19 – 2.89 (m, core + sugar-H), 2.36 (s, core + Cy5-H), 2.11 – 1.85 (m, Cy5-H + linker-H), 1.62 (s, Cy5-H), 1.56 – 1.41 (m, linker-H), 1.32 – 1.00 (m, linker-H + Cy5-H).

Synthesis of Compound 16: A nitrogen-purged reactor vessel was charged with solution of (E)-6-iodo-3-(2-(pyridin-2-yl)vinyl)-1-(tetrahydro-2H-pyran-2-yl)-1H-indazole **14** (500 mg, 1.0 eq, 1.16 mmol), and K_3PO_4 (615 mg, 2.5 eq, 2.90 mmol) in DMF (2 mL). $Pd(dppf)Cl_2 \cdot CH_2Cl_2$ (47 mg, 0.05 eq, 0.058 mmol) and 2-mercapto-*N*-methylbenzamide **15** (293 mg, 1.5 eq, 1.75 mmol) were added and the reaction mixture was purged with N_2 for an additional 5 minutes. The reaction mixture was heated at 80 °C for 12 h. Once TLC indicated

the reaction was complete, the reaction mixture was evaporated using rotary evaporator and purified on a silica column (0-5% MeOH/CH_2Cl_2) to afford 400 mg of compound **16** (0.85 mmol, 73%) as a brown solid.

^{1}H NMR (500 MHz, DMSO-d_6) δ 8.62 (d, *J* = 4.3 Hz, 1H), 8.32 (d, *J* = 8.2 Hz, 1H), 8.10 (s, 1H), 8.01 – 7.91 (m, 2H), 7.83 (t, *J* = 7.2 Hz, 1H), 7.71 (d, *J* = 7.8 Hz, 1H), 7.64 (d, *J* = 16.3 Hz, 1H), 7.41 (t, *J* = 7.5 Hz, 1H), 7.38 – 7.25 (m, 3H), 6.83 (d, *J* = 8.1 Hz, 1H), 5.96 (dd, *J* = 9.7, 2.4 Hz, 1H), 3.90 (s, 4H), 3.84 – 3.72 (m, 1H), 2.49 – 2.39 (m, 1H), 2.09 – 1.97 (m, 2H), 1.82 – 1.68 (m, 1H), 1.59 (s, 2H). ^{13}C NMR (125 MHz, DMSO-d_6) δ 166.52, 155.07, 150.09, 142.20, 142.00, 141.72, 137.42, 133.45, 131.22, 130.98, 130.90, 128.58, 127.98, 127.14, 125.60, 123.36, 123.23, 123.17, 122.97, 122.66, 117.88, 84.47, 67.16, 52.81, 29.38, 25.19, 22.61. MS (ESI) *m/z*: calculated for $C_{27}H_{25}N_3O_3S$ $[M+H]^+$: 472.17, found 472.16.

Synthesis of Compound 17: To a stirred solution of compound **16** (350 mg, 1.0 eq, 0.74 mmol) in MeOH:THF:H_2O (1:1:1) (5 mL) was added NaOH (30 mg, 2.0 eq, 1.48 mmol). The reaction mixture was stirred at room temperature for 2 h. After confirming completion of the reaction by TLC, the reaction mixture was acidified with saturated citric acid solution, extracted with ethyl acetate (20 mL x 3), dried over Na_2SO_4, and concentrated to obtain a crude product. The crude was purified on a silica column (0-10% MeOH/CH_2Cl_2) to afford 305 mg of compound **17** (0.67 mmol, 90%) as a brown solid.

^{1}H NMR (500 MHz, DMSO-d_6) δ 13.28 (s, 1H, -NH), 8.63 (d, *J* = 4.2 Hz, 1H), 8.32 (d, *J* = 8.4 Hz, 1H), 8.10 (s, 1H), 8.02 – 7.90 (m, 2H), 7.83 (t, *J* = 7.6 Hz, 1H), 7.71 (d, *J* = 7.8 Hz, 1H), 7.65 (d, *J* = 16.3 Hz, 1H), 7.42 – 7.33 (m, 2H), 7.33 – 7.28 (m, 1H), 7.23 (t, *J* = 7.5 Hz, 1H), 6.77 (d, *J* = 8.1 Hz, 1H), 5.97 (d, *J* = 10.1 Hz, 1H), 3.95 – 3.86 (m, 1H), 3.84 – 3.72 (m, 1H), 2.47 – 2.38 (m, 1H), 2.08 – 1.95 (m, 2H), 1.76 (tt, *J* = 14.6, 6.7 Hz, 1H), 1.66 – 1.55 (m, 2H). ^{13}C NMR (125 MHz, DMSO-d_6) δ 167.9, 155.1, 150.1, 142.2, 142.2, 141.8, 137.4, 133.0, 131.4, 131.3, 130.9, 128.8, 127.9, 127.5, 125.3, 123.4, 123.2, 123.2, 122.9, 122.6, 117.9, 84.5, 67.2, 29.4, 25.2, 22.6. MS (ESI) *m/z*: calculated for $C_{26}H_{23}N_3O_3S$ $[M+H]^+$: 458.17, found 458.15.

Synthesis of Compound 18: The compounds **17** (250 mg, 1.0 eq, 0.55 mmol) and azido-PEG5 amine (215 mg, 1.3 eq, 0.71 mmol) were dissolved in dry DMF (5 mL). DIPEA (0.2 mL, 1.0 eq, 0.74 mmol) and HATU (316 mg, 1.0 eq, 0.83 mmol) were added to the solution, and the mixture was stirred at room temperature for 1 h. The completion of the reaction was confirmed by TLC analysis. EtOAc (100 mL) was added, washed with water and brine, dried over Na_2SO_4. After filtration and evaporation of the solvent, the crude material was purified on a

silica column (0-100% EtOAc/Hexane) to afford 220 mg of compound **18** (0.29 mmol, 53%) as a viscous yellow liquid.

^{1}H NMR (500 MHz, DMSO-d_6) δ 8.62 (d, *J* = 5.3 Hz, 1H), 8.51 (t, *J* = 5.7 Hz, 1H), 8.22 (d, *J* = 8.5 Hz, 1H), 7.98 – 7.90 (m, 2H), 7.82 (t, *J* = 7.6 Hz, 1H), 7.69 (d, *J* = 7.8 Hz, 1H), 7.61 (d, *J* = 16.3 Hz, 1H), 7.51 (d, *J* = 7.5 Hz, 1H), 7.37 – 7.23 (m, 3H), 7.22 (d, *J* = 8.5 Hz, 1H), 7.00 (d, *J* = 7.9 Hz, 1H), 5.96 – 5.88 (m, 1H), 3.90 (d, *J* = 11.3 Hz, 1H), 3.83 – 3.72 (m, 1H), 3.60 – 3.34 (m, 24H), 2.10 – 1.97 (m, 2H), 1.65 – 1.54 (m, 2H), 1.30 – 1.21 (m, 2H). ^{13}C NMR (125 MHz, DMSO-d_6) δ 167.9, 162.8, 162.1, 155.1, 150.1, 142.1, 141.6, 137.4, 136.7, 136.5, 133.3, 130.9, 130.7, 130.0, 128.4, 127.2, 126.4, 123.3, 123.2, 122.5, 122.1, 115.7, 84.5, 70.3, 70.3, 70.2, 70.2, 70.2, 70.1, 70.1, 70.0, 69.7, 69.3, 69.2, 67.1, 50.4, 44.6, 38.7, 36.3, 31.2, 29.4, 25.2, 22.6.

Synthesis of Compound 19: Compound **18** (200 mg, 1.0 eq, 0.27 mmol) was dissolved in trifluoroacetic acid (2 mL), and the reaction mixture was stirred at 60 °C for 1 h until completion. The completion of the reaction was monitored using TLC. Trifluoroacetic acid (TFA) was then evaporated under vacuum distillation. The saturated aqueous sodium bicarbonate solution was added dropwise to the reaction mixture until the pH reached 7-8. The resulting compound was extracted with ethyl acetate (3 × 20 mL), washed with brine, dried over anhydrous Na_2SO_4, and concentrated under reduced pressure to obtain the crude product. The crude product was purified on a silica column (0-5% MeOH/CH_2Cl_2) to afford 124 mg of compound **19** (0.19 mmol, 70%) as a yellow solid.

^{1}H NMR (500 MHz, DMSO-d_6) δ 13.47 (s, 1H), 8.70 (d, *J* = 5.0 Hz, 1H), 8.56 (t, *J* = 5.7 Hz, 1H), 8.26 (d, *J* = 8.4 Hz, 1H), 8.06 (d, *J* = 16.5 Hz, 1H), 7.98 (t, *J* = 7.9 Hz, 1H), 7.86 (d, *J* = 8.1 Hz, 1H), 7.66 (t, *J* = 8.2 Hz, 2H), 7.56 (dd, *J* = 7.5, 1.8 Hz, 1H), 7.47 – 7.32 (m, 3H), 7.26 (d, *J* = 8.4 Hz, 1H), 7.13 (d, *J* = 7.6 Hz, 1H), 3.87 – 3.33 (m, 24H). MS (ESI) *m/z*: calculated for $C_{33}H_{39}N_7O_6S$ $[M+H]^+$: 686.25, found 686.29.

Synthesis of Compound 20: In a 5 mL round-bottom flask, compound **12** (150 mg, 1.0 eq, 0.007 mmol) was dissolved in 1 mL of deionized (DI) water. A solution of compound **19** (60 mg, 13 eq, 0.09 mmol) prepared in 1 mL of DMF was then added. Subsequently, $CuSO_4 \cdot 5H_2O$ (10 mol% per alkyne, in 0.1 mL water) and sodium ascorbate (15 mol% per alkyne, in 0.1 mL water) were added sequentially. The reaction mixture was stirred at 40 °C for 10 h. After completion, the crude product was purified using a 3 kDa dialysis membrane first dialyzed against 20% dimethylacetamide (DMAc) and then against water overnight to afford the purified compound. The aqueous solution was lyophilized to afford 174 mg of compound **20**

(0.0063 mmol, 90%) as a light brown solid. HPLC purity: 99.7%, retention time: 11.02 minutes.

^{1}H NMR (500 MHz, DMSO-d$_6$) δ 13.29 (s, 10H, Axi-NH), 8.53 (d, *J* = 5.0 Hz, 10H, Axi-H), 8.47 – 8.37 (m, 18H, Axi-H), 8.13 (d, *J* = 8.5 Hz, 10H, Axi-H), 7.97 – 7.80 (m, 45H, Triazoles + amides + Axi-H), 7.80 – 7.64 (m, 33H, Triazoles + amides), 7.59 (d, *J* = 7.9 Hz, 10H, Axi-H), 7.55 – 7.47 (m, 17H, Axi-H), 7.45 – 7.37 (m, 12H, Axi-H), 7.29 – 7.15 (m, 30H, Axi-H), 7.15 – 7.05 (m, 26H, Axi-H + D-Ar-H), 6.98 (d, *J* = 7.8 Hz, 10H, Axi-H), 5.17 – 4.86 (m, sugar-H), 4.85 – 4.65 (m, Sugar-H), 4.62 – 4.26 (m, sugar-H), 4.22 – 3.90 (m, sugar-H + PEG-H), 3.89 – 3.48 (m, sugar-H + PEG-H), 3.41 – 3.18 (m, PEG-H), 3.17 – 2.86 (m, PEG-H), 2.77 – 2.62 (m, core-H), 2.62 – 2.44 (m, core-H), 2.29 (s, 41H, core-H + linker-H), 2.16 – 1.93 (m, 42H, core-H + linker-H), 1.84 – 1.66 (m, core-H + linker-H, 42H). ^{13}C NMR (125 MHz, DMSO-d$_6$) δ 168.0, 166.1, 155.3, 152.2, 150.0, 144.1, 140.3, 137.4, 135.9, 133.1, 130.8, 130.6, 129.7, 128.4, 126.7, 125.9, 124.8, 123.1, 123.0, 122.2, 120.7, 106.7, 97.2, 93.7, 79.7, 79.4, 79.2, 73.3, 73.1, 73.0, 72.3, 71.9, 71.8, 70.5, 70.4, 70.3, 70.2, 70.1, 70.0, 69.5, 69.4, 69.2, 68.8, 63.8, 61.2, 51.2, 49.7, 38.8, 33.3, 25.6, 24.8, 21.9.

Synthesis of Tre-D-Axi-Cy5 (compound 21): Compound **20** (50 mg, 1.0 eq, 0.0019 mmol) was dissolved in 1 mL of deionized water in a 10 mL microwave vial. To this solution, Cy5-azide (5.5 mg, 3.5 eq, 0.0066 mmol) dissolved in 1 mL of DMF was added. Subsequently, aqueous solutions of $CuSO_4 \cdot 5H_2O$ (10 mol% per alkyne) and sodium ascorbate (15 mol% per alkyne) were introduced sequentially under stirring. The reaction mixture was subjected to microwave irradiation at 40 °C for 10 h. Upon completion, as confirmed by HPLC analysis, the reaction mixture was purified by dialysis using a 1 kDa MWCO membrane against deionized water for 12 h to remove residual reagents and solvents. The resulting aqueous solution was lyophilized to afford 47 mg of compound **21** (0.0017 mmol, 89%) as a blue solid.

^{1}H NMR (500 MHz, DMSO-d$_6$) δ 8.70 – 8.36 (m, 26H, Axi-H + Cy5-H), 8.36 – 8.01 (m, 25H, Axi-H), 8.03 – 7.69 (m, 83H, Tre-D-triazoles + Axi-H), 7.67 – 7.33 (m, 32H, Tre-D-triazoles + amides), 7.33 – 7.18 (m, 21H, Axi-Ar + Tre-D-triazoles), 7.18 – 7.11 (m, 17H, Axi-Ar), 7.11 (s, 16H, Tre-D-Ar), 7.03 – 6.89 (m, 10H, Axi-Ar), 6.50 (s, 2H, Cy5-H), 6.24 (d, *J*=13.4, 4H, Cy5-H), 5.48 – 4.68 (m, sugar-H + core-H), 4.59 – 4.28 (m, PEG + sugar-H), 4.23 – 3.94 (m, PEG), 3.84 – 3.40 (m, PEG-H), 3.18 – 2.81 (m, PEG + sugar-H + core-H), 2.63 – 2.50 (m, core + linker-H), 2.35 – 1.91 (m, core + Cy5-H+ linker-H), 1.88 – 1.59 (m, Cy5-H+ linker-H, core-H), 1.55 – 1.41 (m, Cy5-H+ linker-H), 1.27 – 0.99 (m, Cy5-H+ linker-H).

1.4 Stability studies

Tre-D-Axitinib formulation shelf-stability studies

Tre-D-Axitinib formulations (50 mg/mL in PBS) were passed through 0.4 µm sterile filters and stored at room temperature, 4°C and 37°C. The aliquots were analyzed *via* HPLC for the purity at 0 h, 7-day, 15-day, and 30day time points **(Figure S47-48)**.

Tre-D-Axitinib formulation stability studies at physiological conditions

In vitro drug release studies were performed under two conditions: plasma conditions using phosphate-buffered saline (PBS) at pH 7.4, and intracellular conditions using citrate buffer at pH 5.5, supplemented with esterase at 37°C in an incubator. The Tre-D-Axi conjugate was dissolved at a concentration of 2 mg/mL in each respective buffer and underwent incubation at 37°C with continuous shaking to replicate the physiological conditions. At predetermined time intervals, samples were withdrawn, promptly quenched with an equivalent volume of methanol, and stored at -20°C until further analysis. The released drug was quantified using high-performance liquid chromatography (HPLC), by comparing the results to a standard curve established for the free drug, Axitinib, on the HPLC system.

2. Supporting Figures

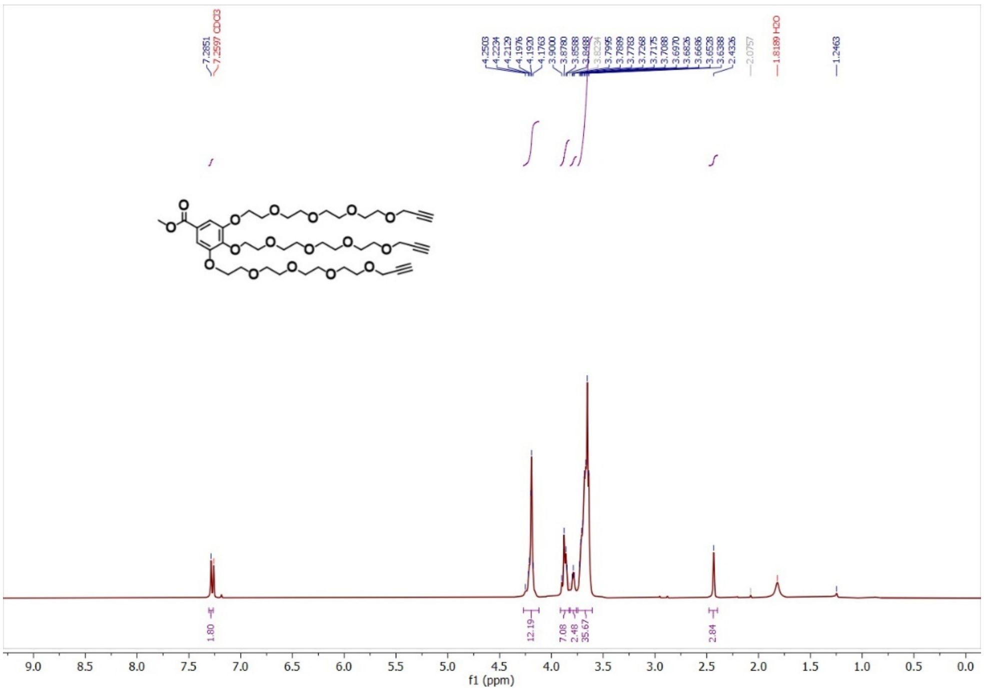

Figure S1. ^{1}H NMR (500 MHz, $CDCl_3$) spectrum of compound **2**.

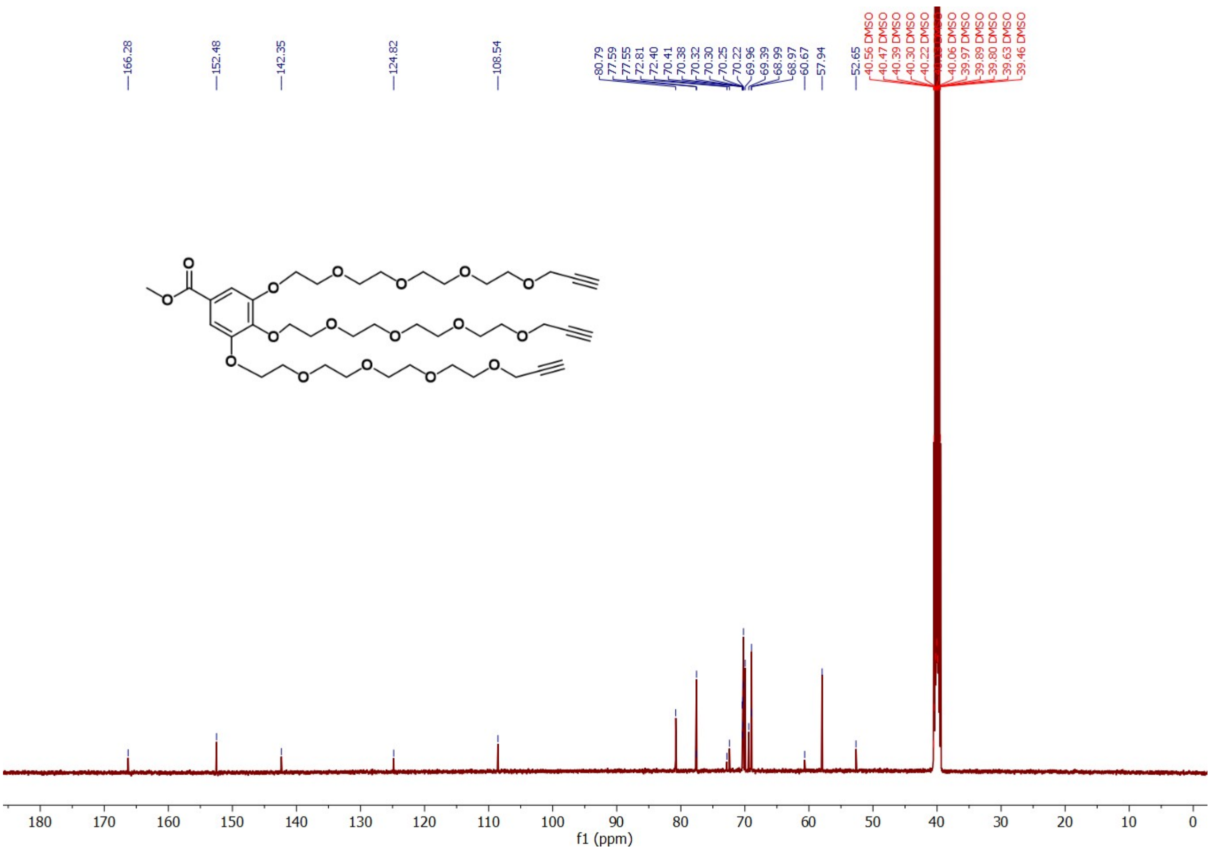

Figure S2. ^{13}C NMR (125 MHz, $CDCl_3$) spectrum of compound **2**.

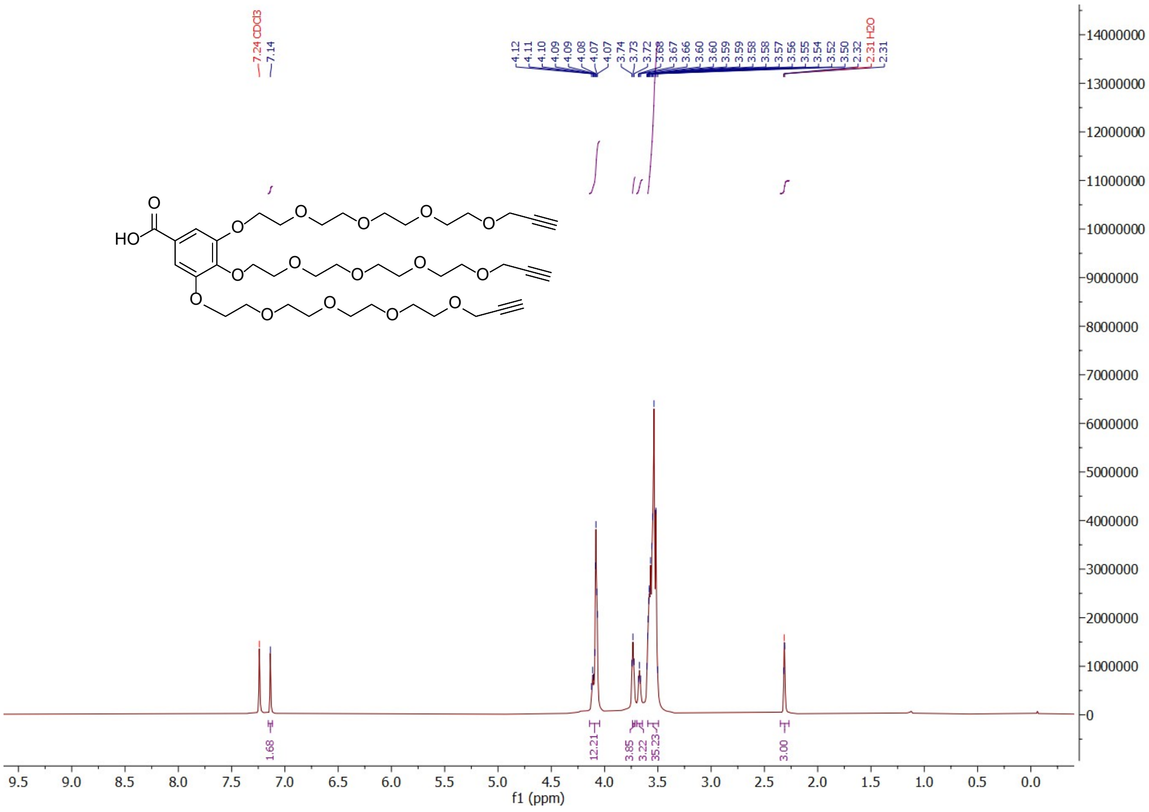

Figure S3. ^{1}H NMR (500 MHz, $CDCl_3$) spectrum of compound **3**.

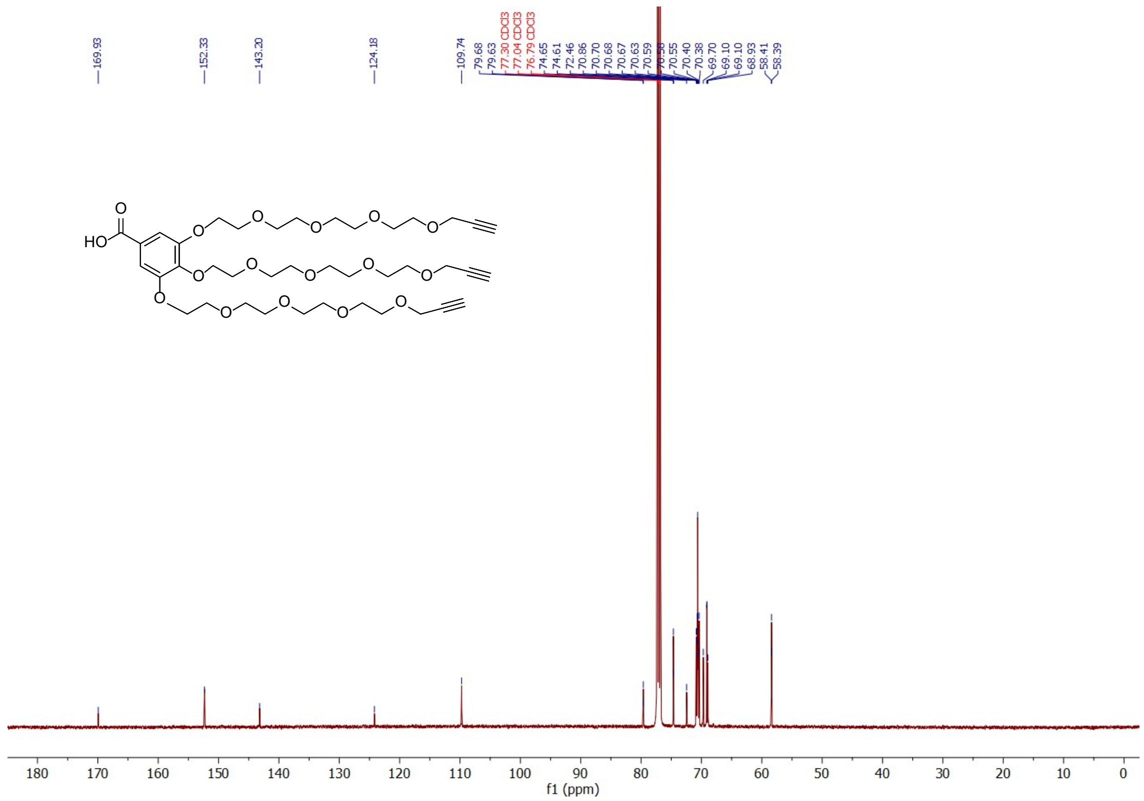

Figure S4. ^{13}C NMR (125 MHz, $CDCl_3$) spectrum of compound **3.**

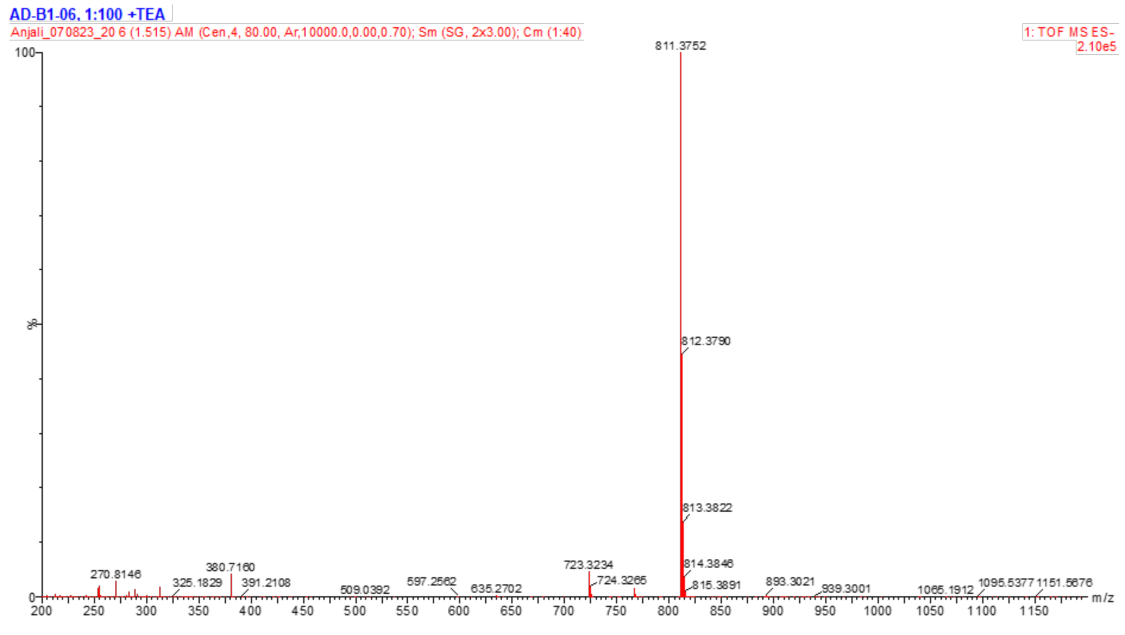

Figure S5. Mass spectra (ESI-MS) of compound **3**.

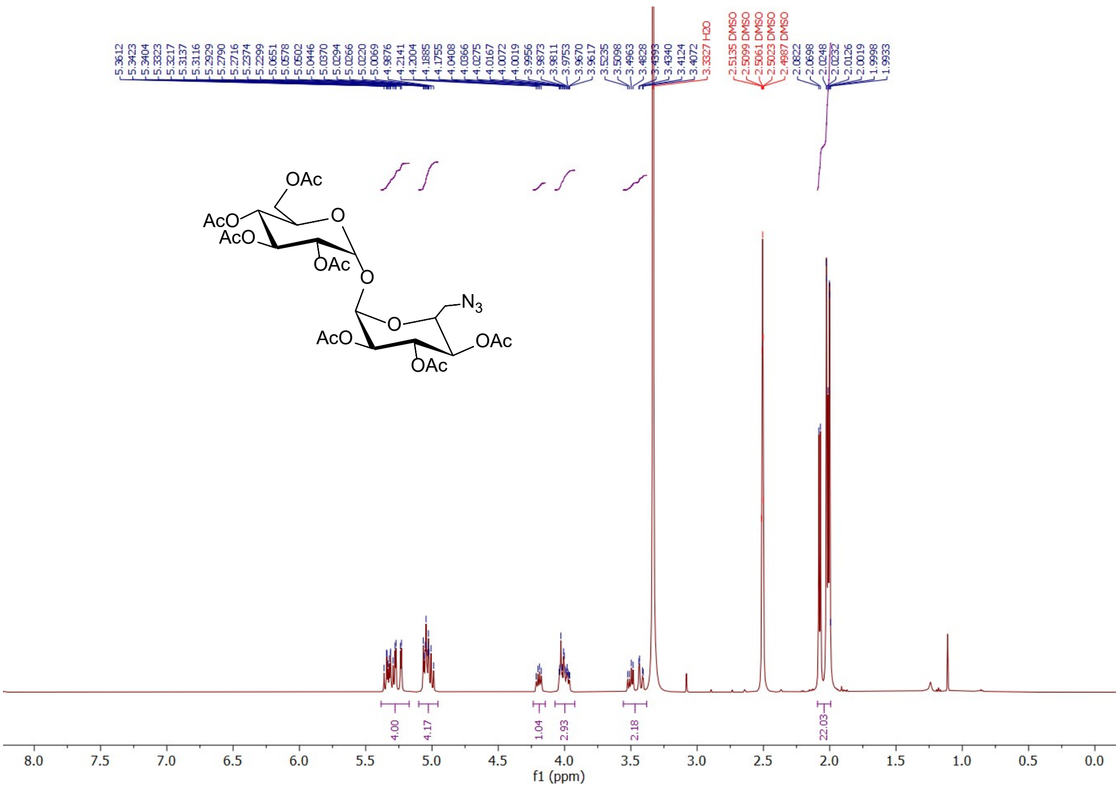

Figure S6. ^{1}H NMR (500 MHz, DMSO-d$_6$) spectrum of compound **4**.

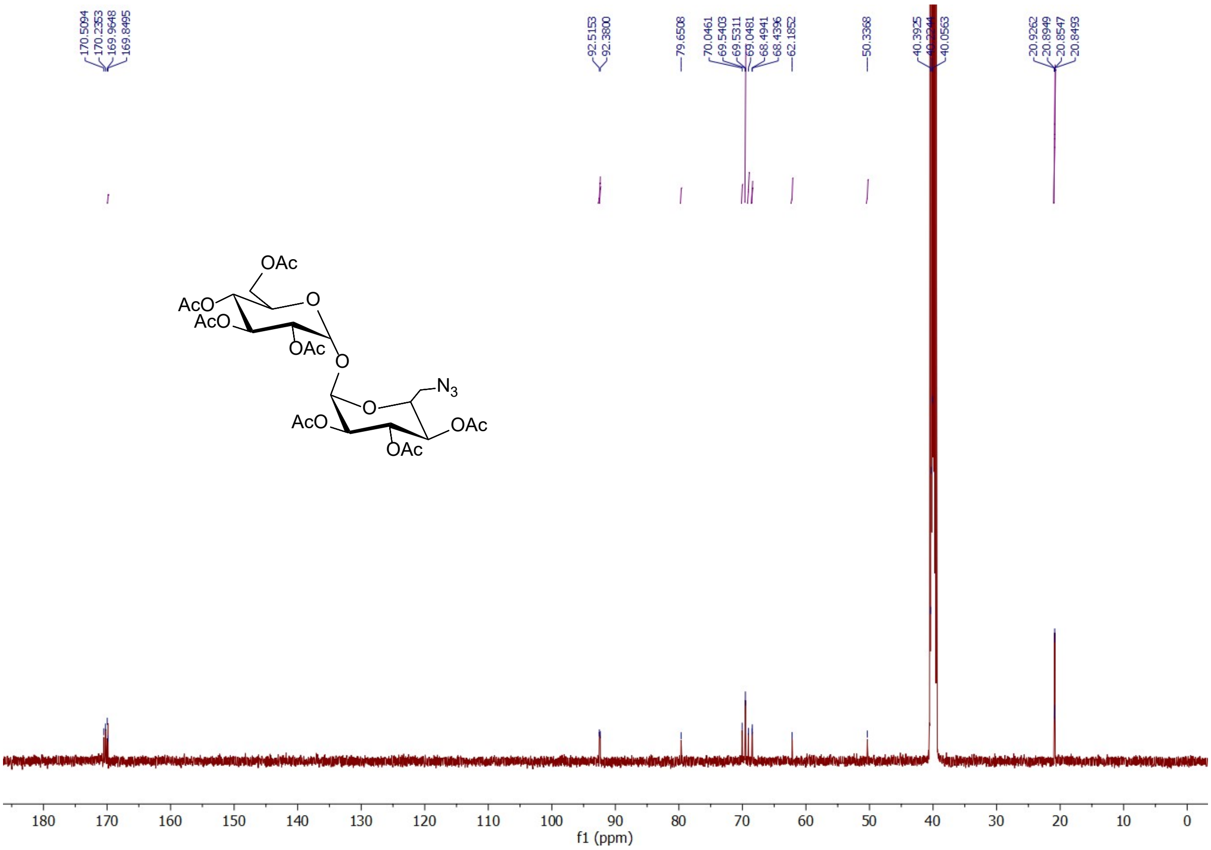

Figure S7. ^{13}C NMR (125 MHz, DMSO-d$_6$) spectrum of compound **4**.

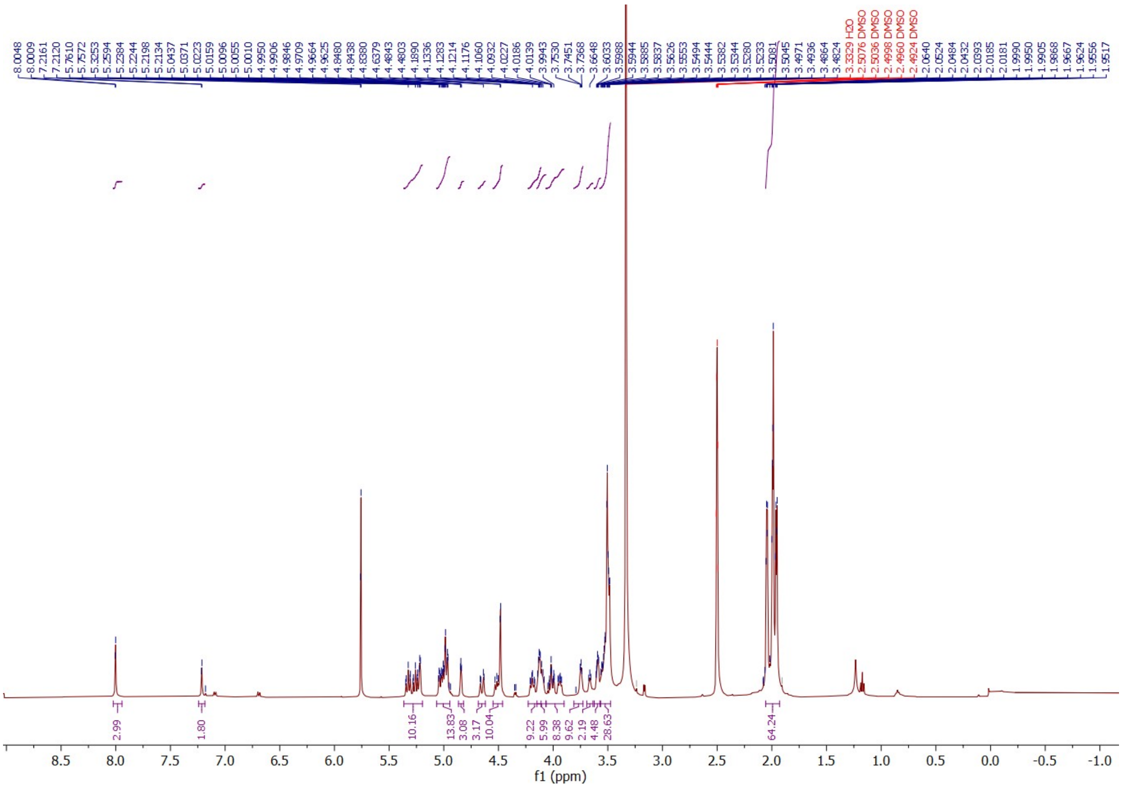

Figure S8. ^{1}H NMR (500 MHz, DMSO-d_6) spectrum of compound **5**.

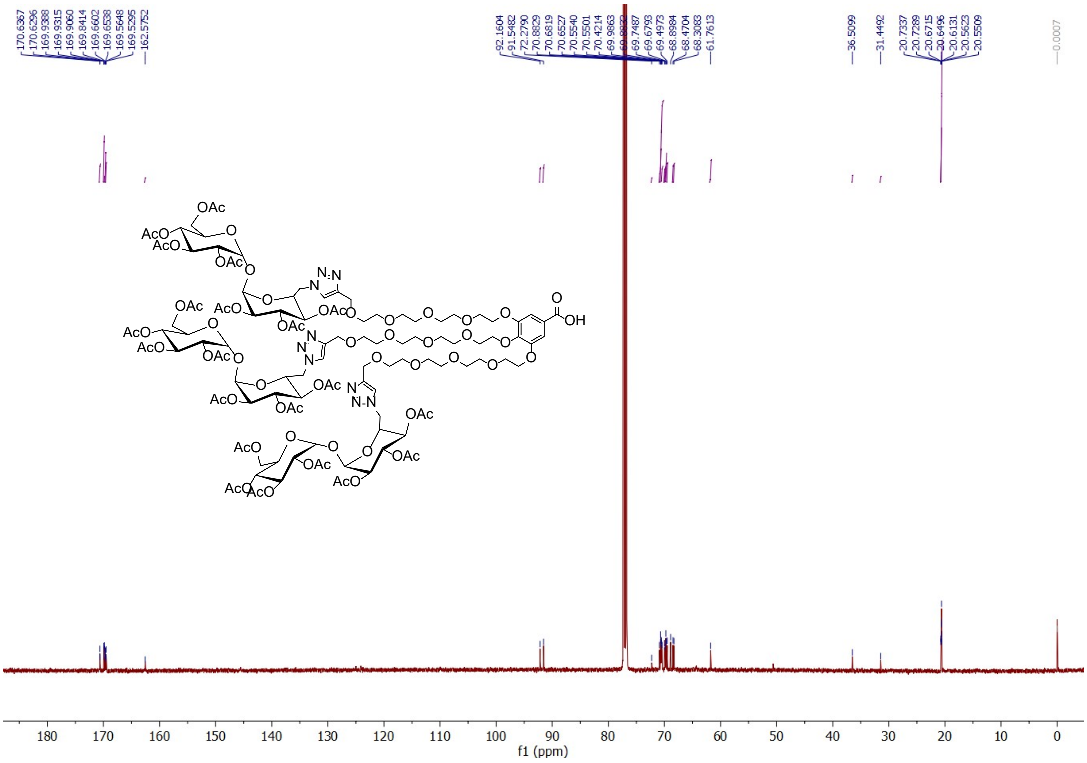

Figure S9. ^{13}C NMR (125 MHz, $CDCl_3$) spectrum of compound **5**.

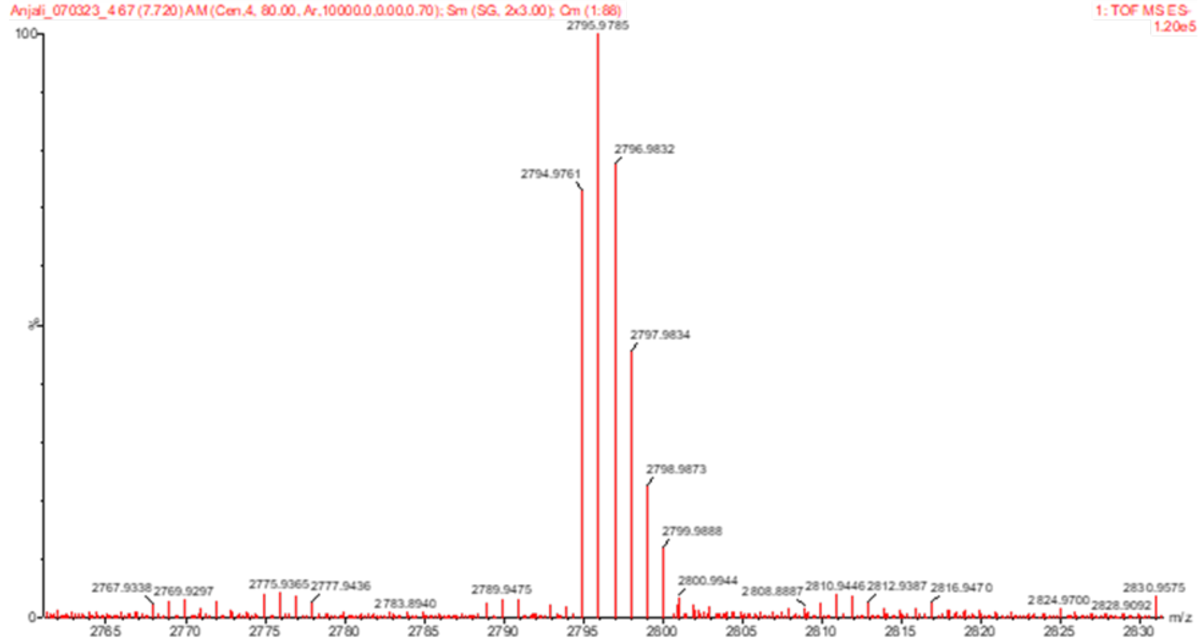

Figure S10. Mass spectra (ESI-MS) of compound **5.**

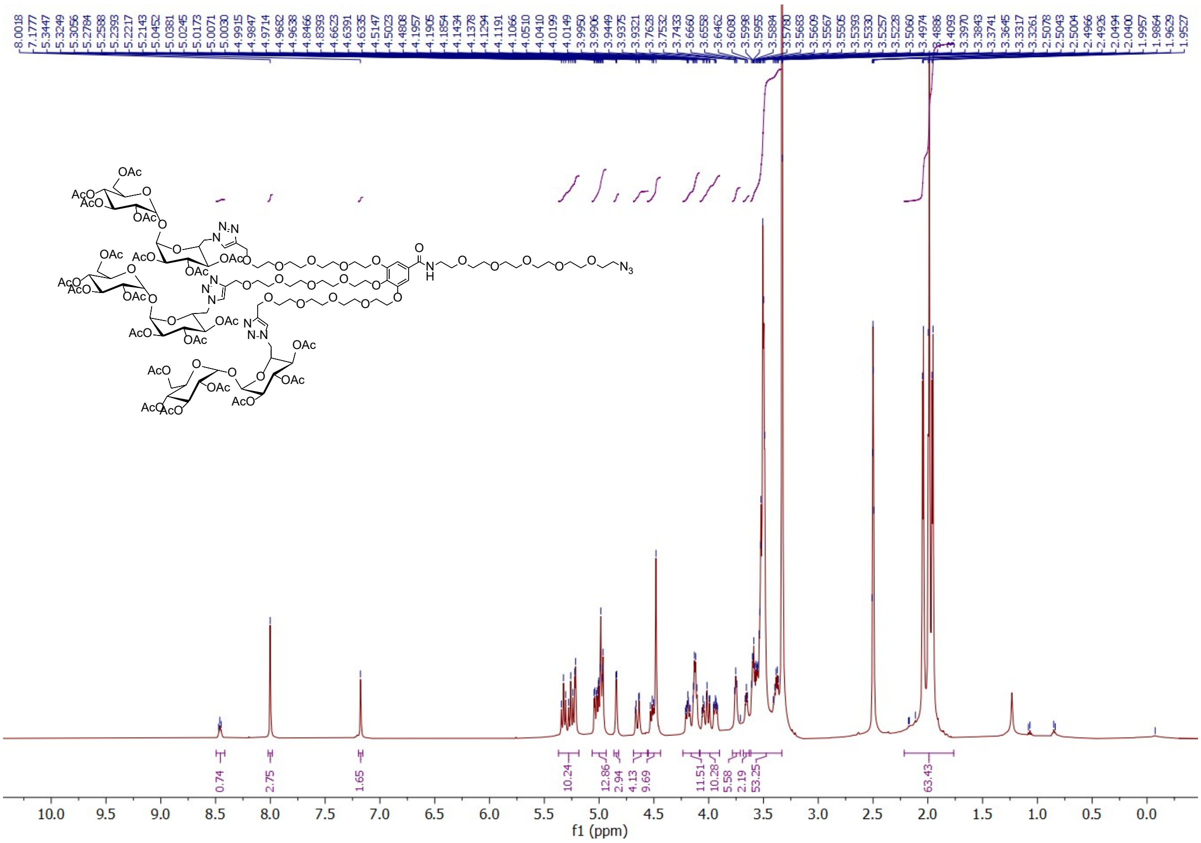

Figure S11. ^{1}H NMR (500 MHz, DMSO-d_6) spectrum of compound **7**.

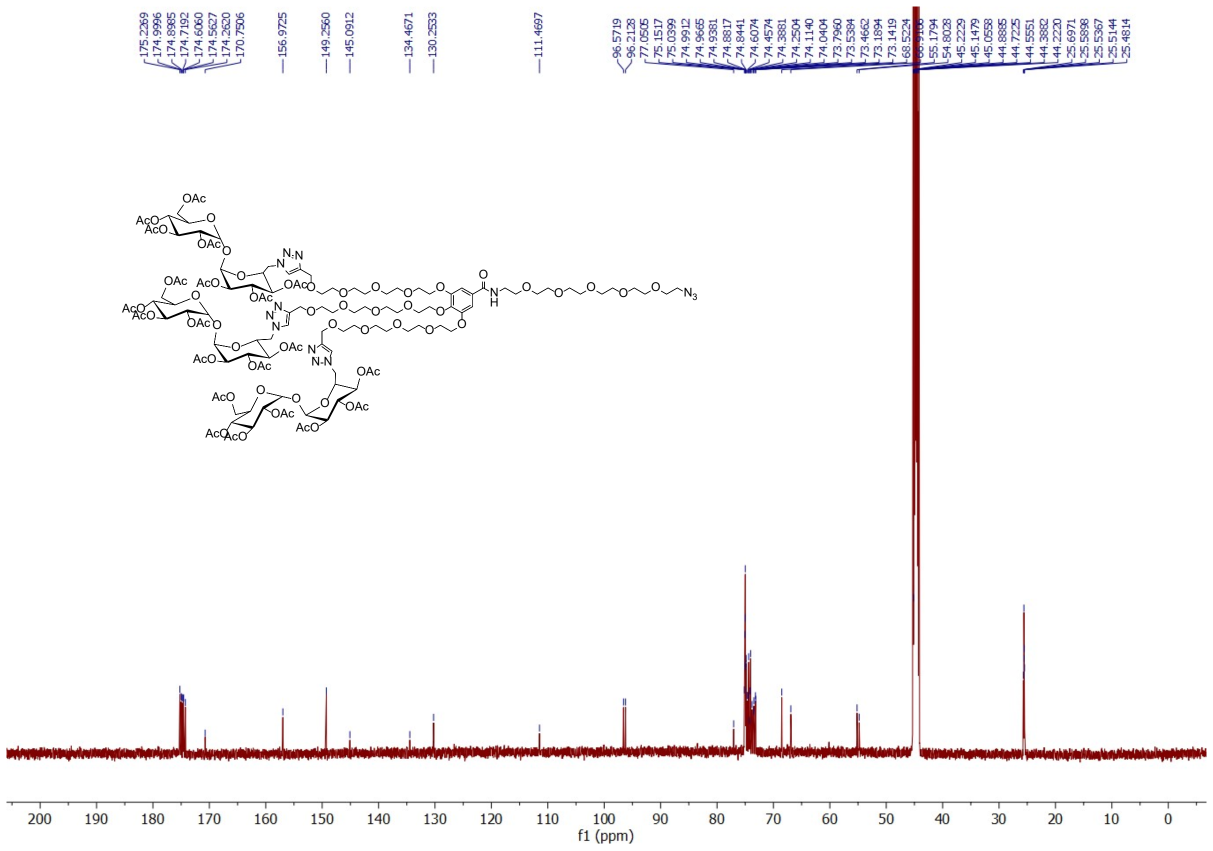

Figure S12. ^{13}C NMR (125 MHz, DMSO-d$_6$) spectrum of compound **7**.

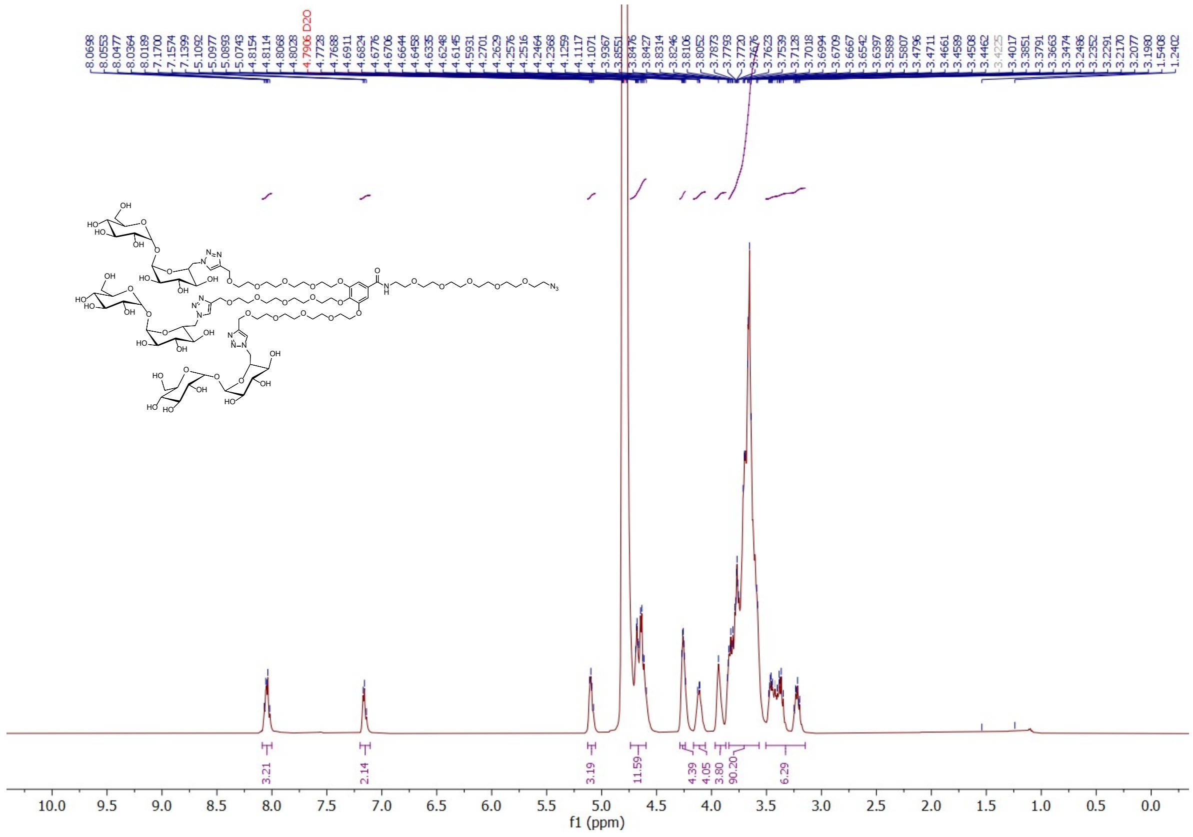

Figure S13. ^{1}H NMR (500 MHz, D$_2$O) spectrum of compound **8**.

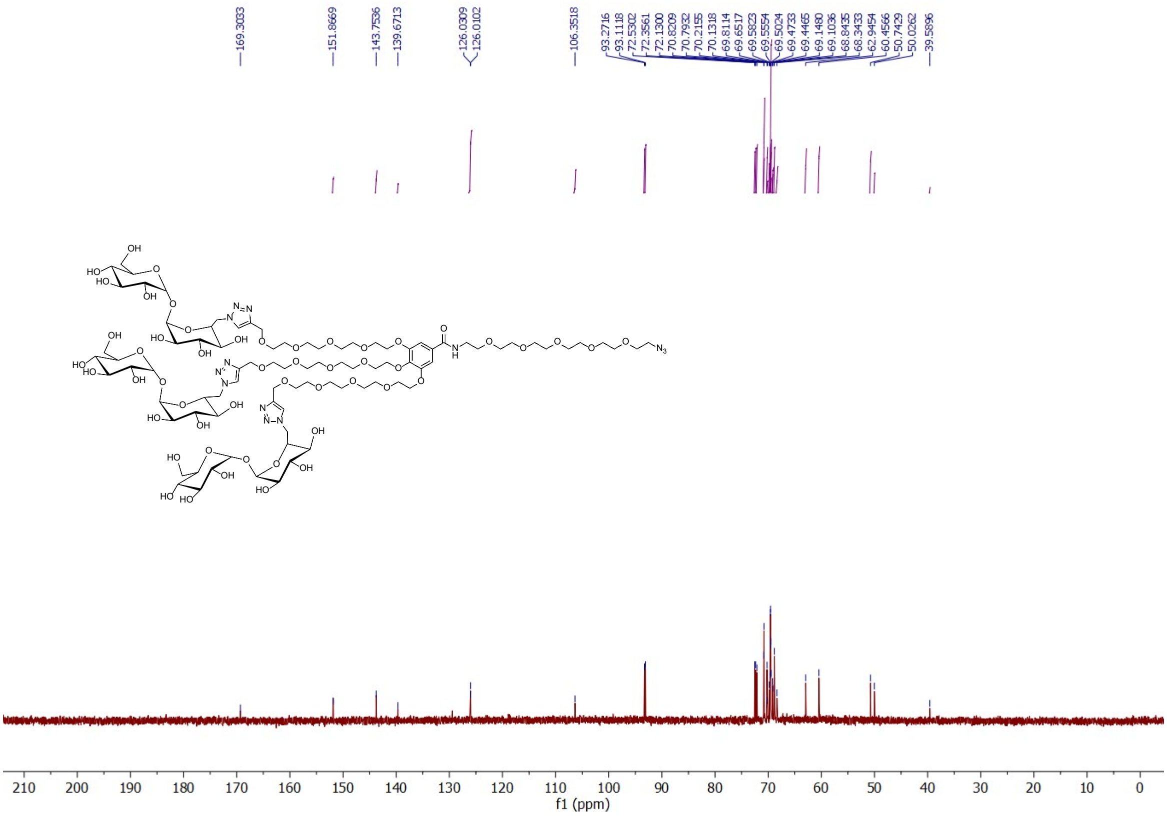

Figure S14. ^{13}C NMR (125 MHz, D_2O) spectrum of compound **8**.

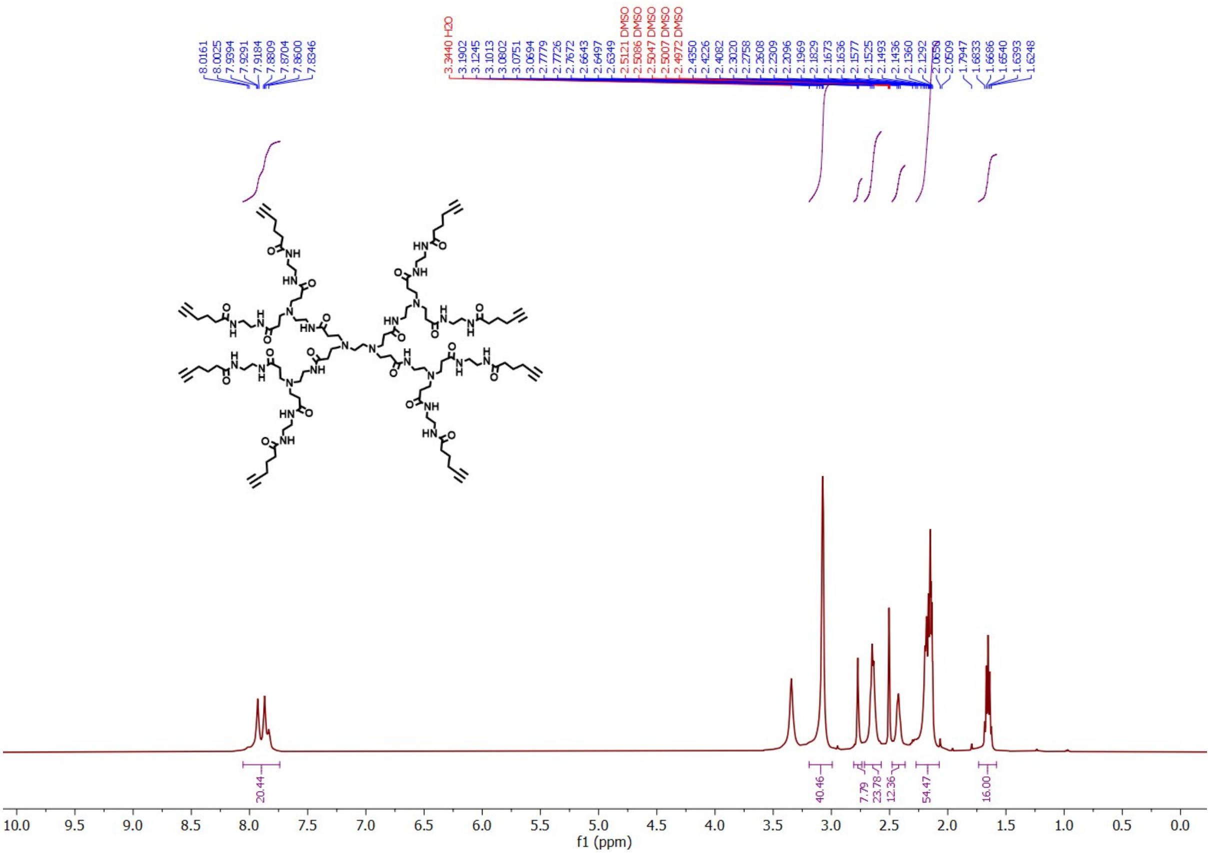

Figure S15. ^{1}H NMR (500 MHz, DMSO-d_6) spectrum of compound **9**.

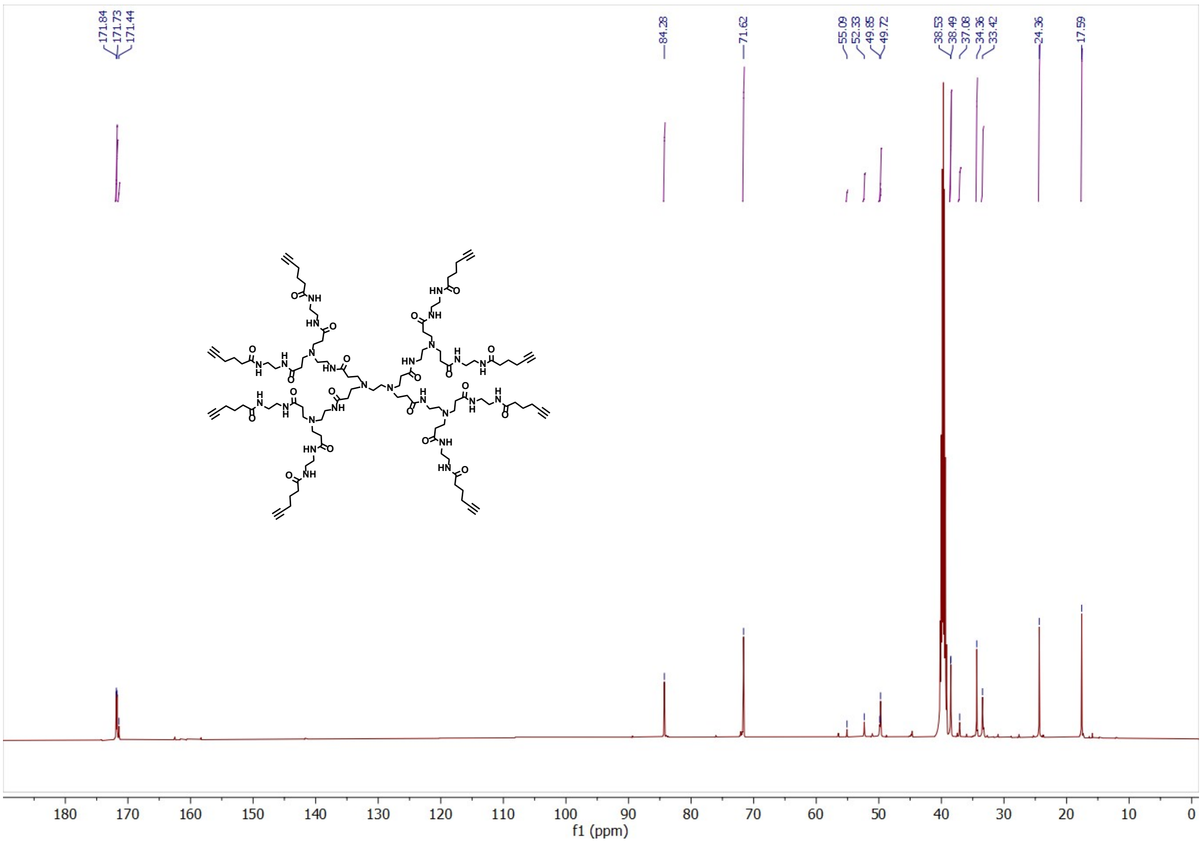

Figure S16. ^{13}C NMR (125 MHz, DMSO-d$_6$) spectrum of compound **9**.

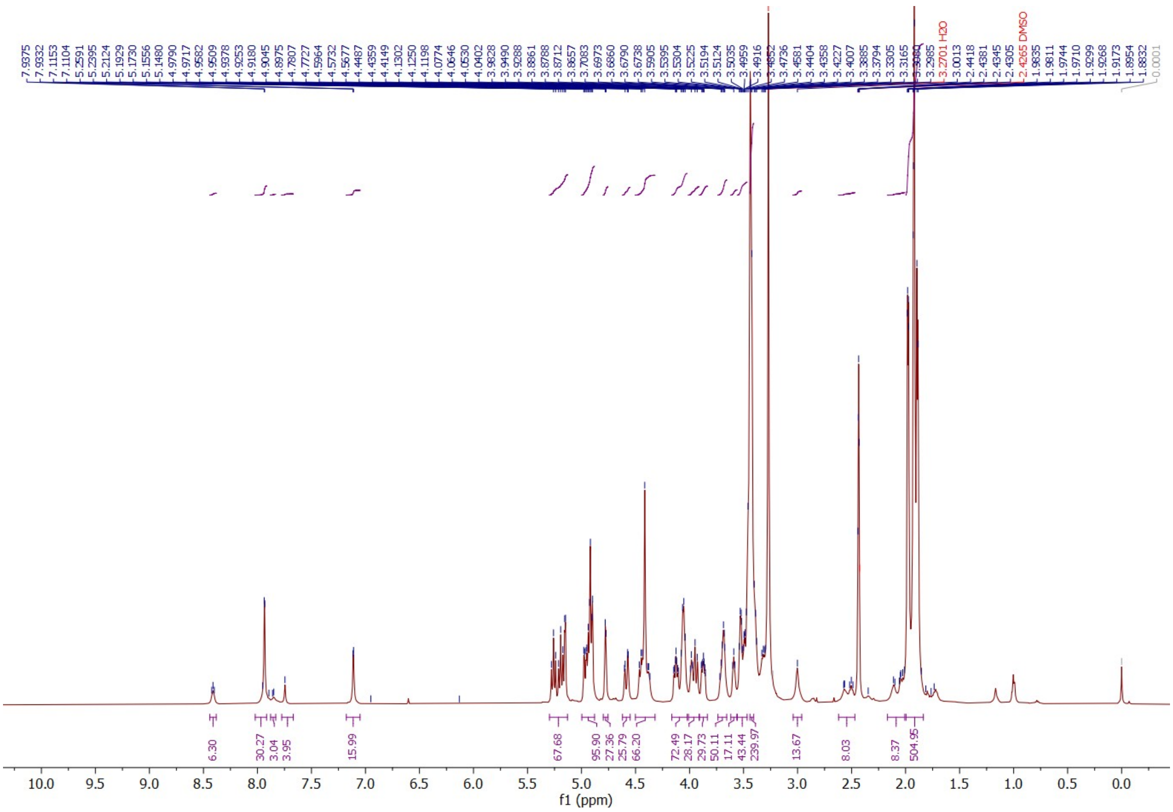

Figure S17. ^{1}H NMR (500 MHz, DMSO-d$_6$) spectrum of protected trehalose dendrimer (compound **10**).

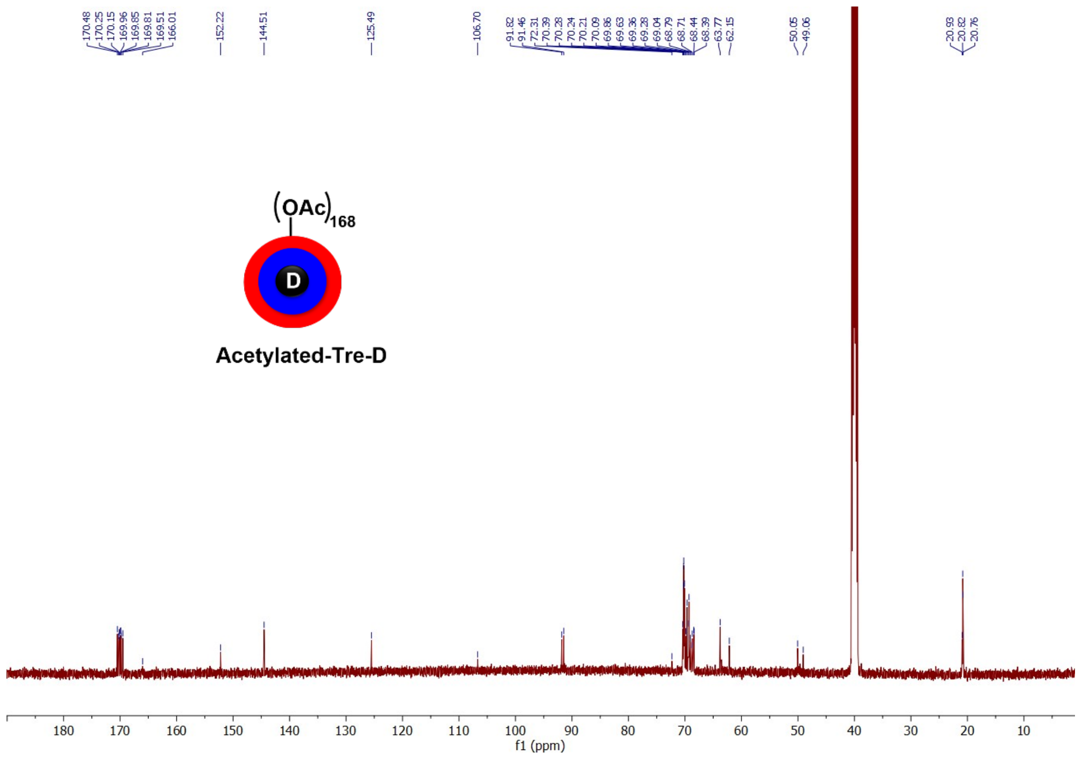

Figure S18. ^{13}C NMR (125 MHz, DMSO-d$_6$) spectrum of compound **10**.

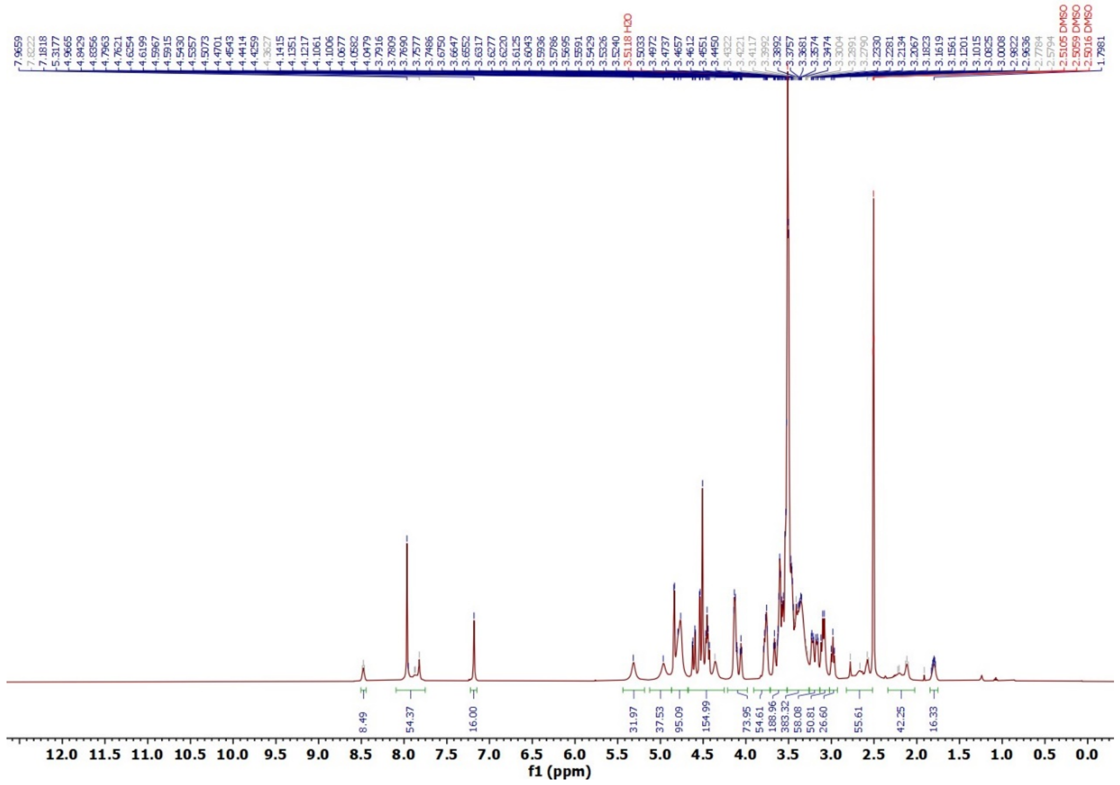

Figure S19. ^{1}H NMR (500 MHz, DMSO-d$_6$) spectrum of compound **11**.

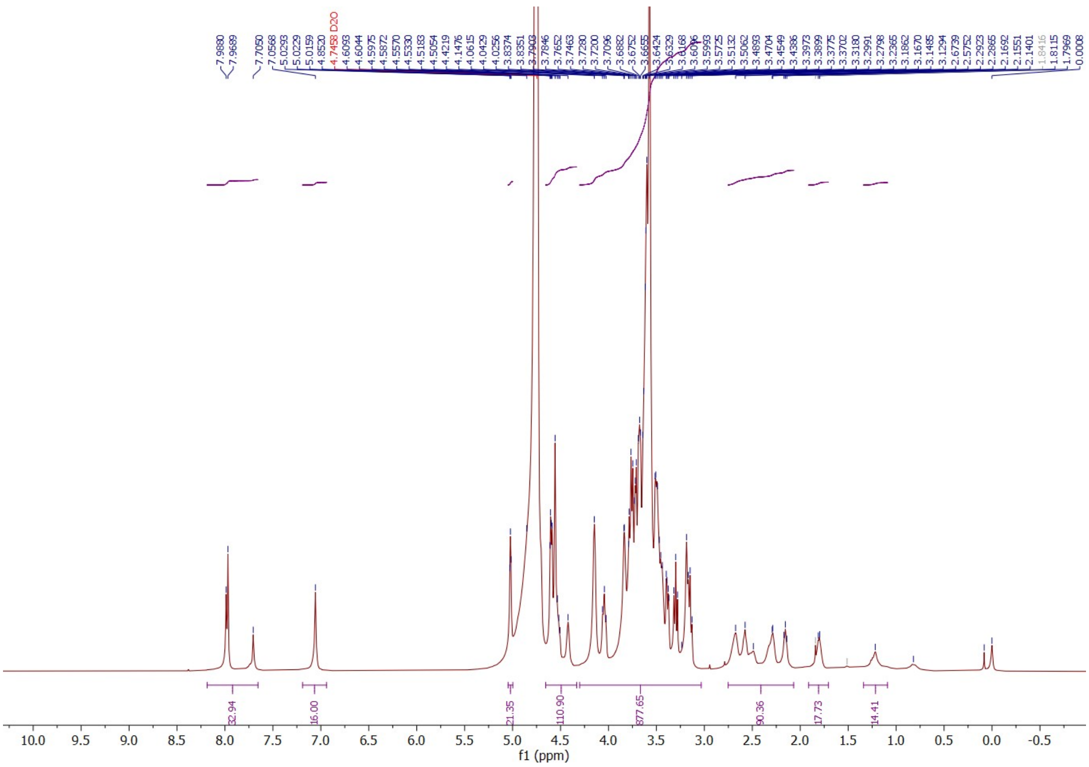

Figure S20. ^{1}H NMR (500 MHz, D_2O) spectrum of compound **11** after deprotection.

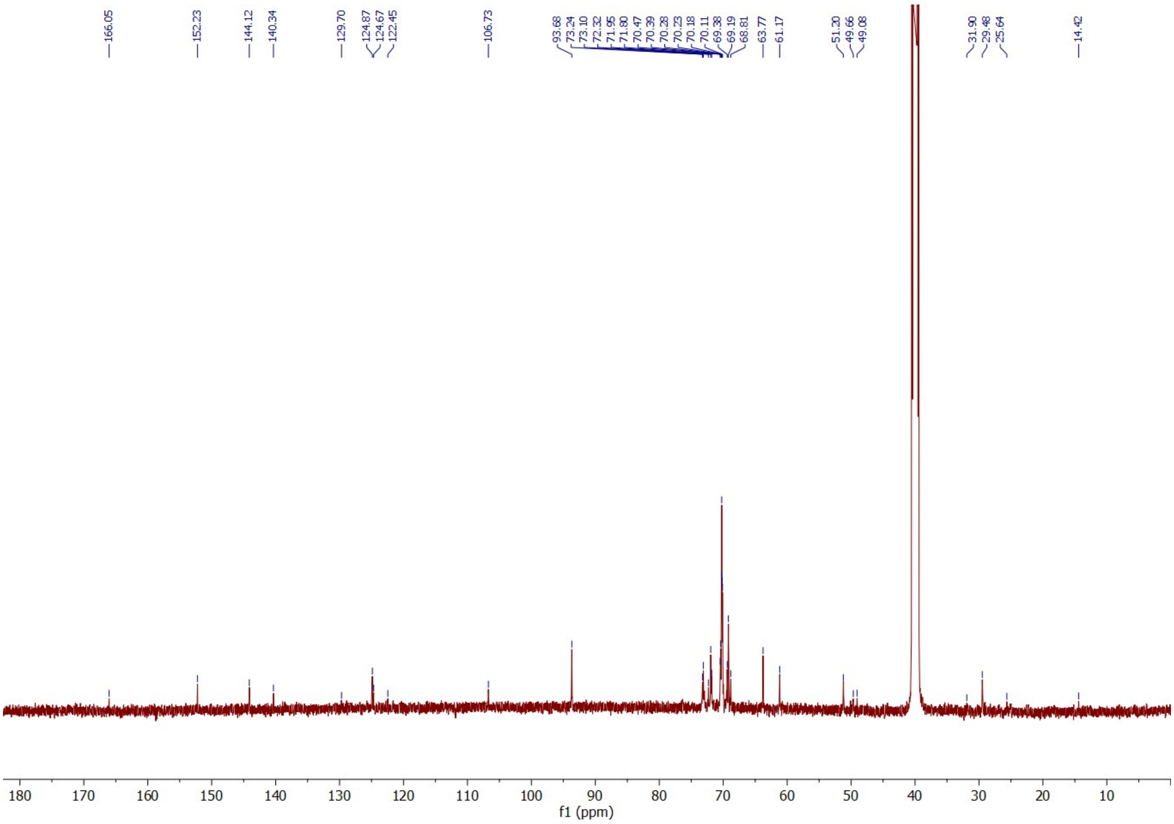

Figure S21. ^{13}C NMR (125 MHz, DMSO-d_6) spectrum of compound **11**.

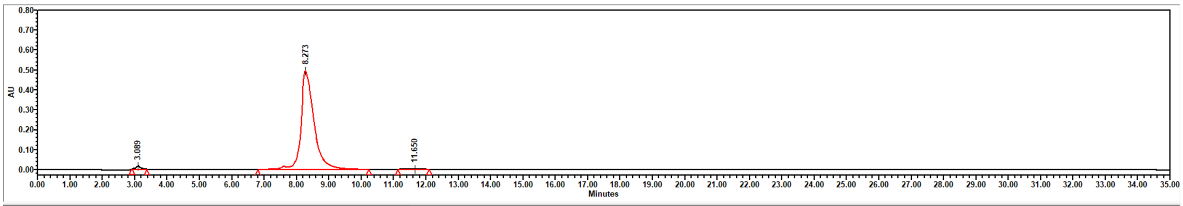

	Name	Retention Time (min)	Area (µV*sec)	% Area	Height (µV)	Int Type	Amount	Units	Peak Type	Peak Codes
1		3.089	154586	1.08	15076	bb			Unknown	
2		8.273	14065421	98.45	497006	bb			Unknown	
3		11.650	66740	0.47	1996	bb			Unknown	

Figure S22. HPLC chromatogram confirming >98% purity of compound **11**.

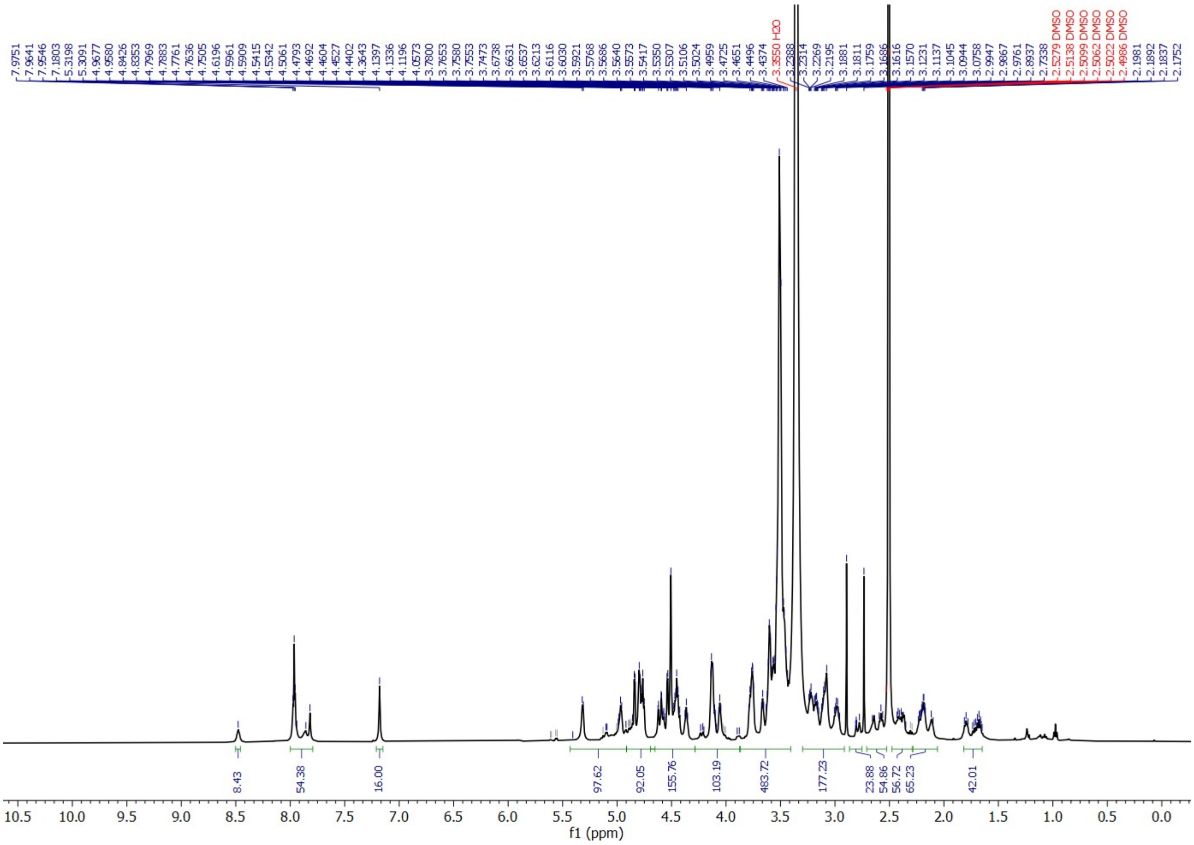

Figure S23. ^{1}H NMR (500 MHz, DMSO-d$_6$) spectrum of compound **12**.

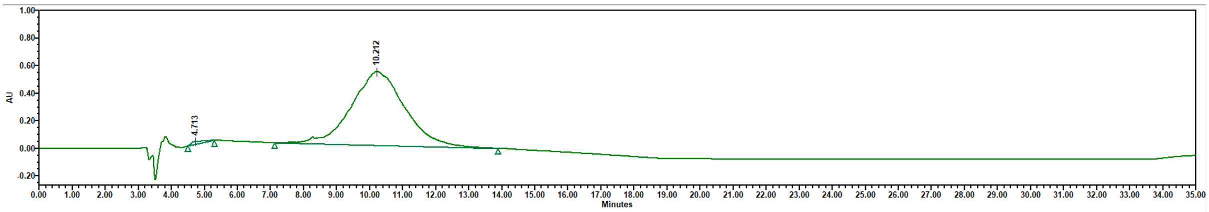

	Name	Retention Time (min)	Area (µV*sec)	% Area	Height (µV)	Int Type	Peak Type
1		4.713	530290	0.88	20048	bb	Unknown
2		10.212	59842829	99.12	533317	bb	Unknown

Figure S24. HPLC chromatogram of compound **12** exhibiting >99% purity.

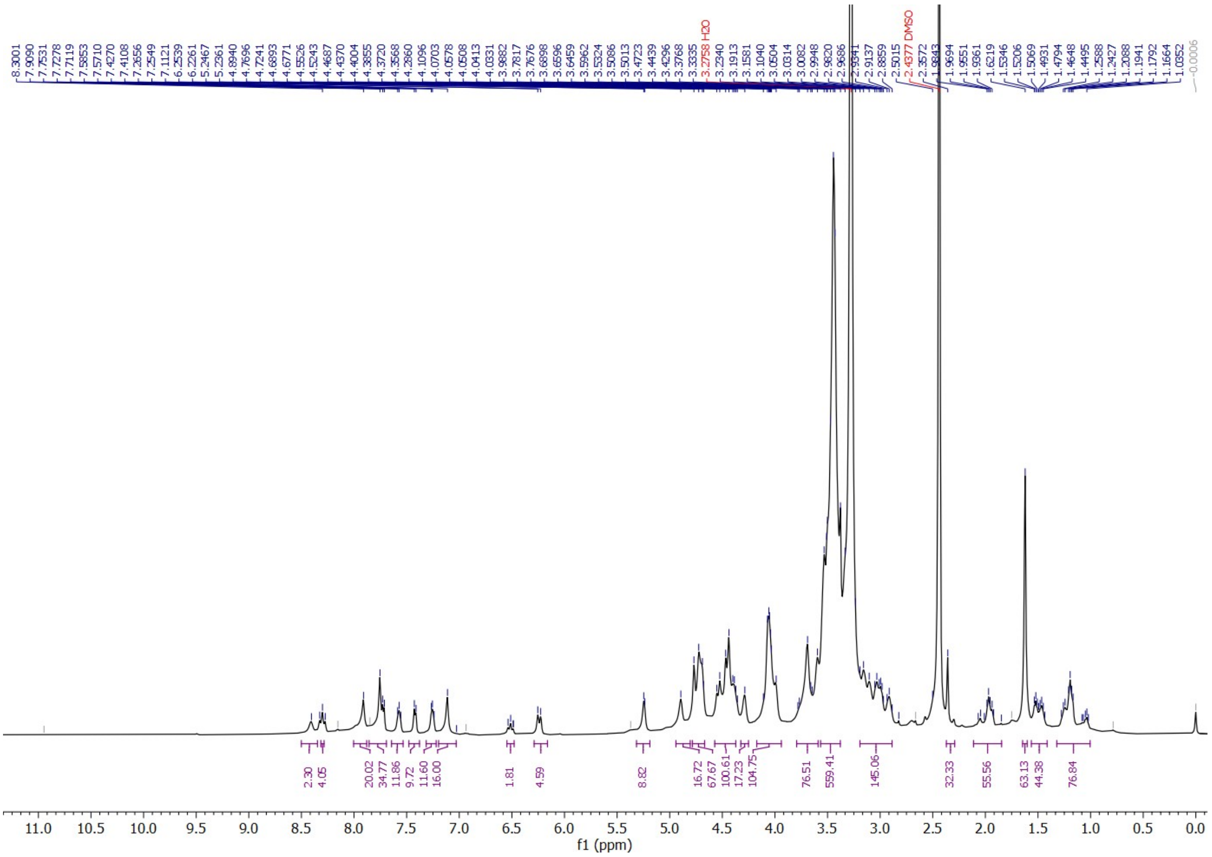

Figure S25. ^{1}H NMR (500 MHz, DMSO-d_6) spectrum of compound **13**.

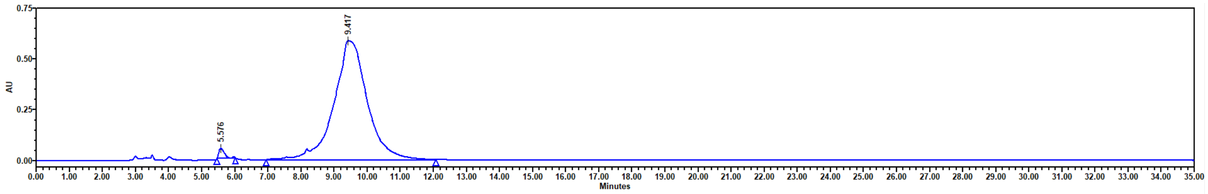

	Name	Retention Time (min)	Area (µV*sec)	% Area	Height (µV)	Int Type	Amount	Units	Peak Type	Peak Codes
1		5.576	585768	1.41	49716	bb			Unknown	
2		9.417	40979325	98.59	586247	bb			Unknown	

Figure S26. HPLC chromatogram of Tre-D-Cy5 (compound **13**) indicating >99% purity.

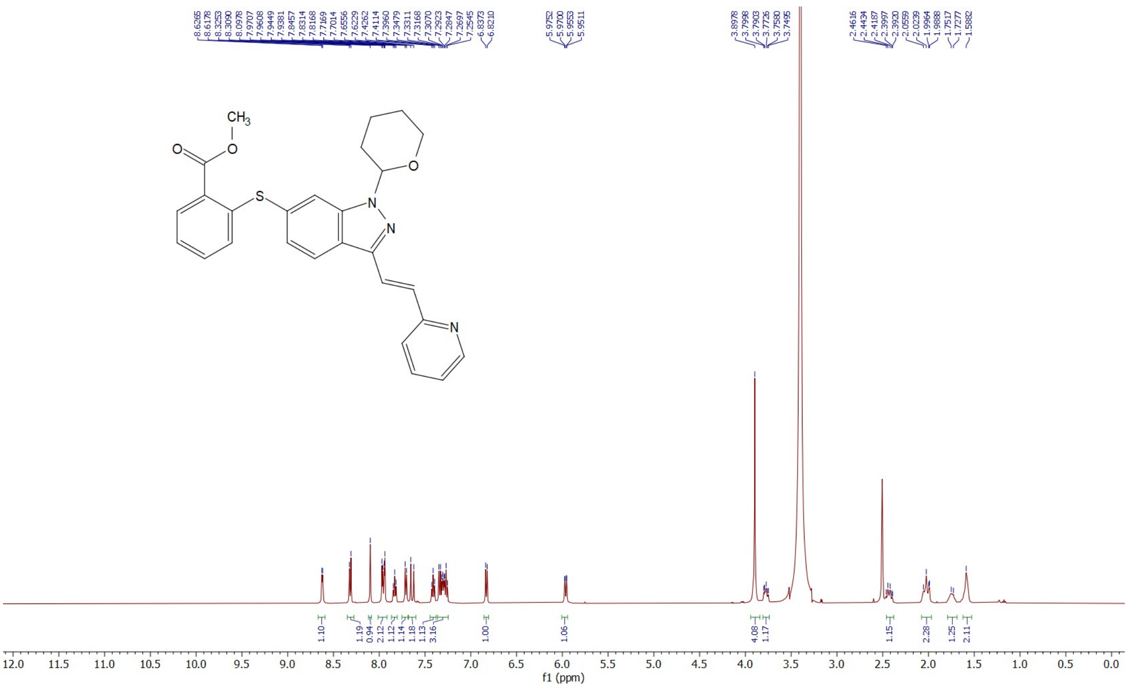

Figure S27. ^{1}H NMR (500 MHz, DMSO-d$_6$) spectrum of compound **16**.

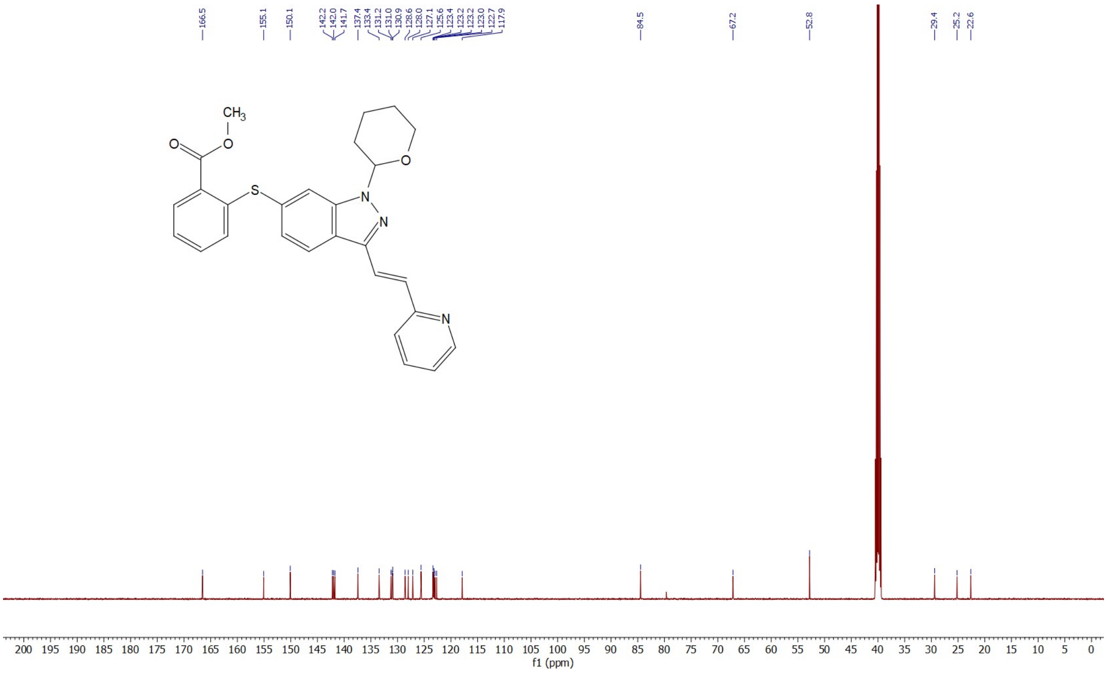

Figure S28. ^{13}C NMR (125 MHz, DMSO-d$_6$) spectrum of compound **16**.

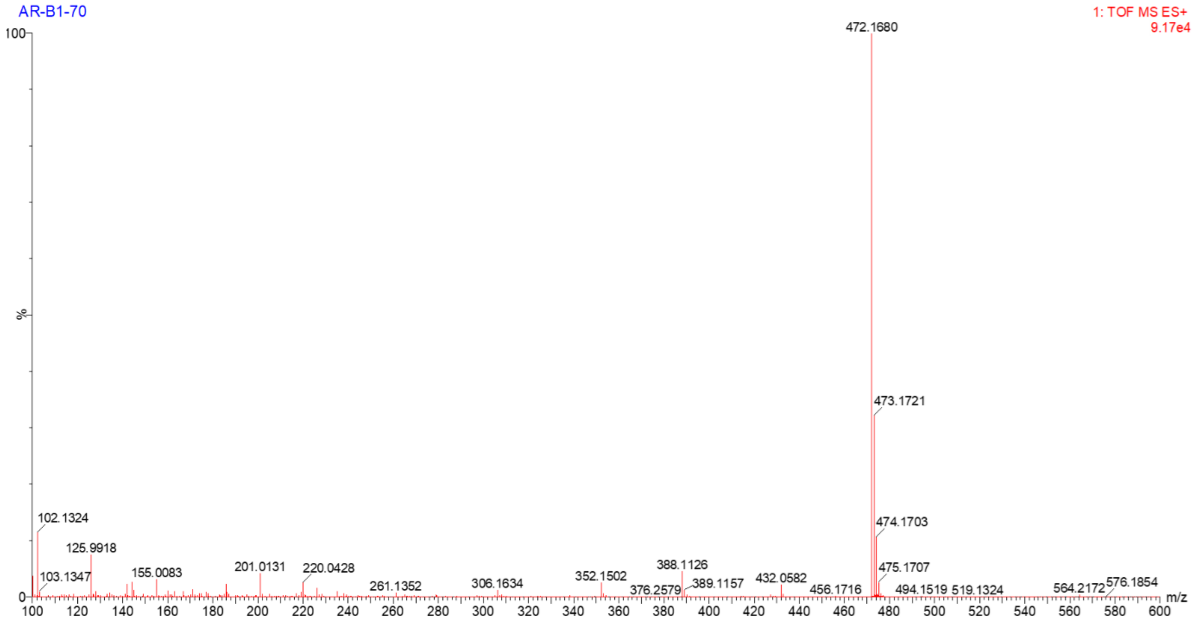

Figure S29. Mass spectra (ESI-MS) of compound **16**.

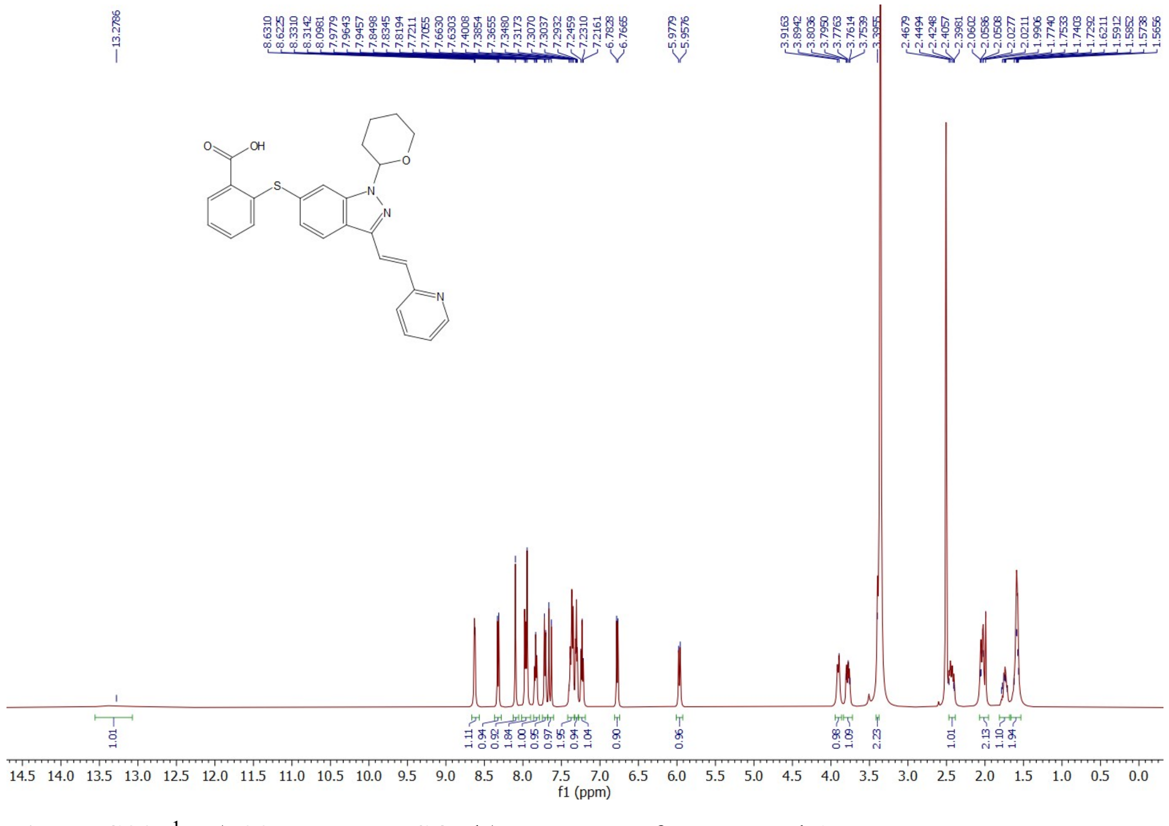

Figure S30. ^{1}H (500 MHz, DMSO-d_6) spectrum of compound **17**.

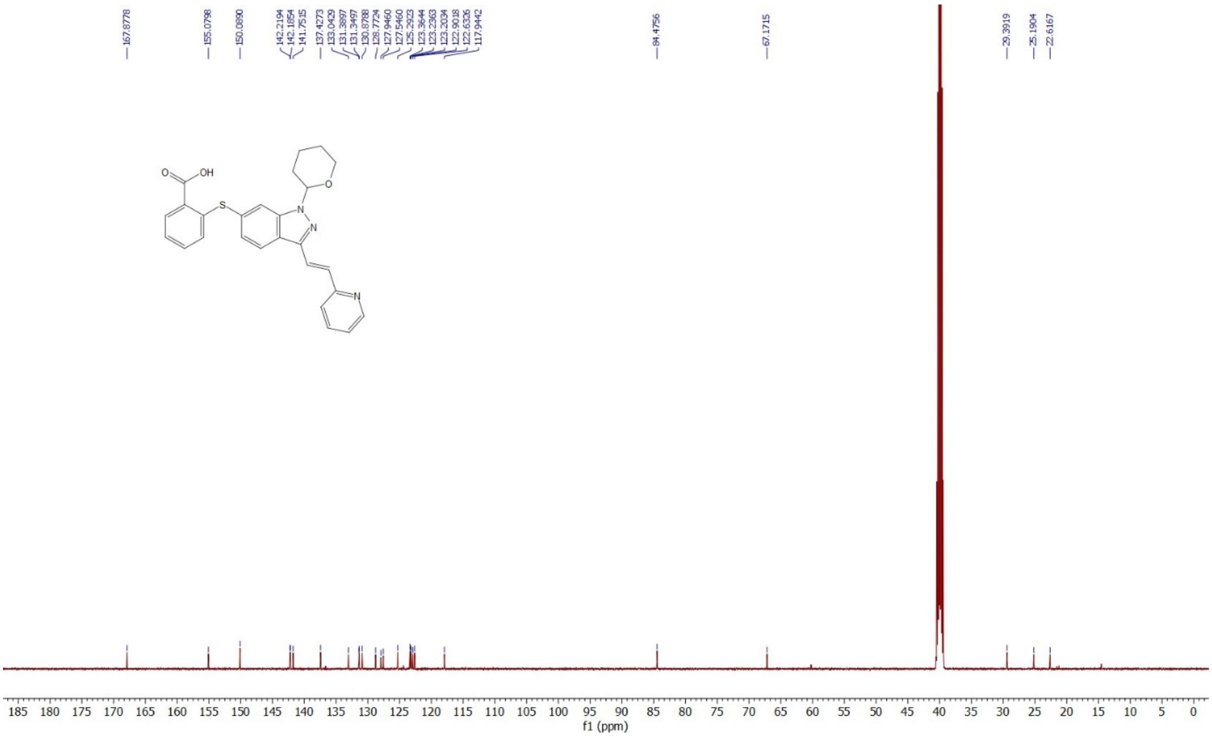

Figure S31. ^{13}C NMR (125 MHz, DMSO-d$_6$) spectrum of compound **17**.

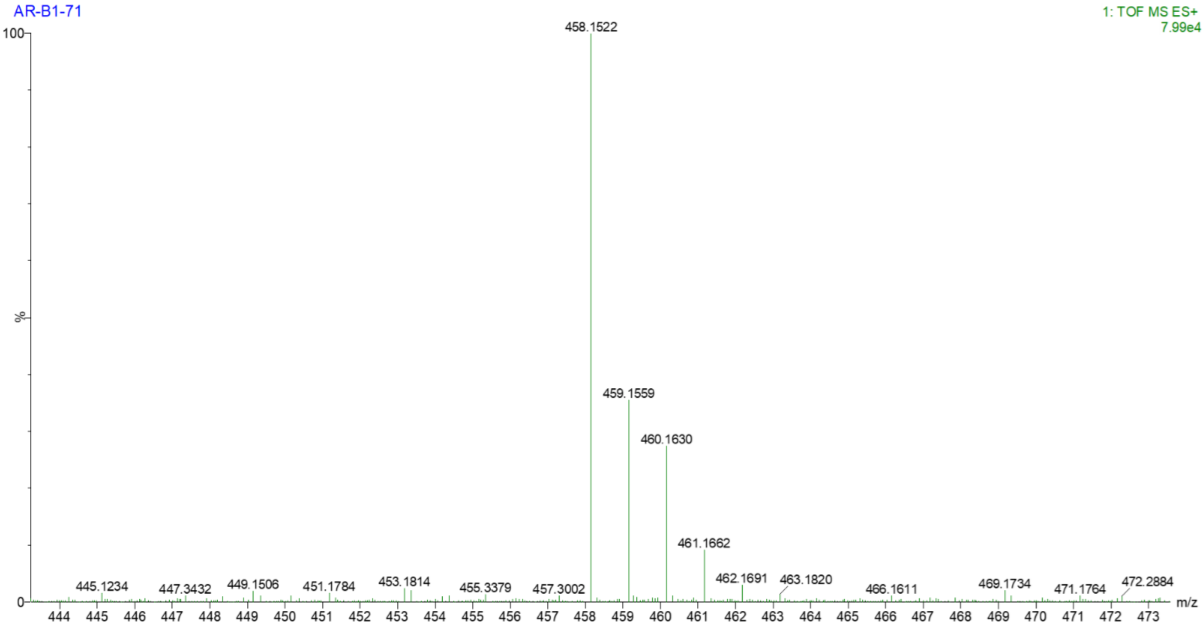

Figure S32. Mass spectra (ESI-MS) of compound **17**.

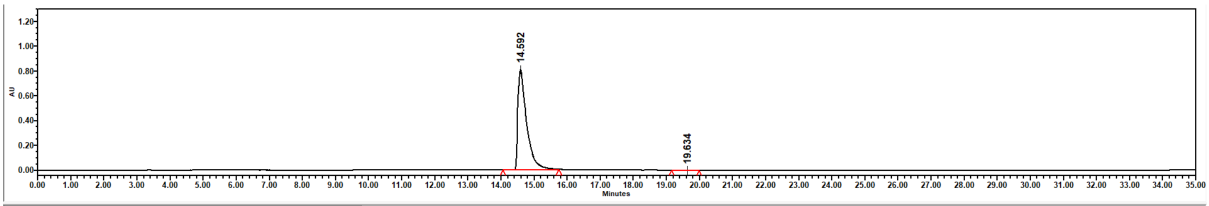

	Name	Retention Time (min)	Area (µV*sec)	% Area	Height (µV)	Int Type	Amount	Units	Peak Type	Peak Codes
1		14.592	14897803	99.98	811771	bb			Unknown	
2		19.634	2775	0.02	141	bb			Unknown	

Figure S33. HPLC chromatogram confirming >99% purity of compound **17**.

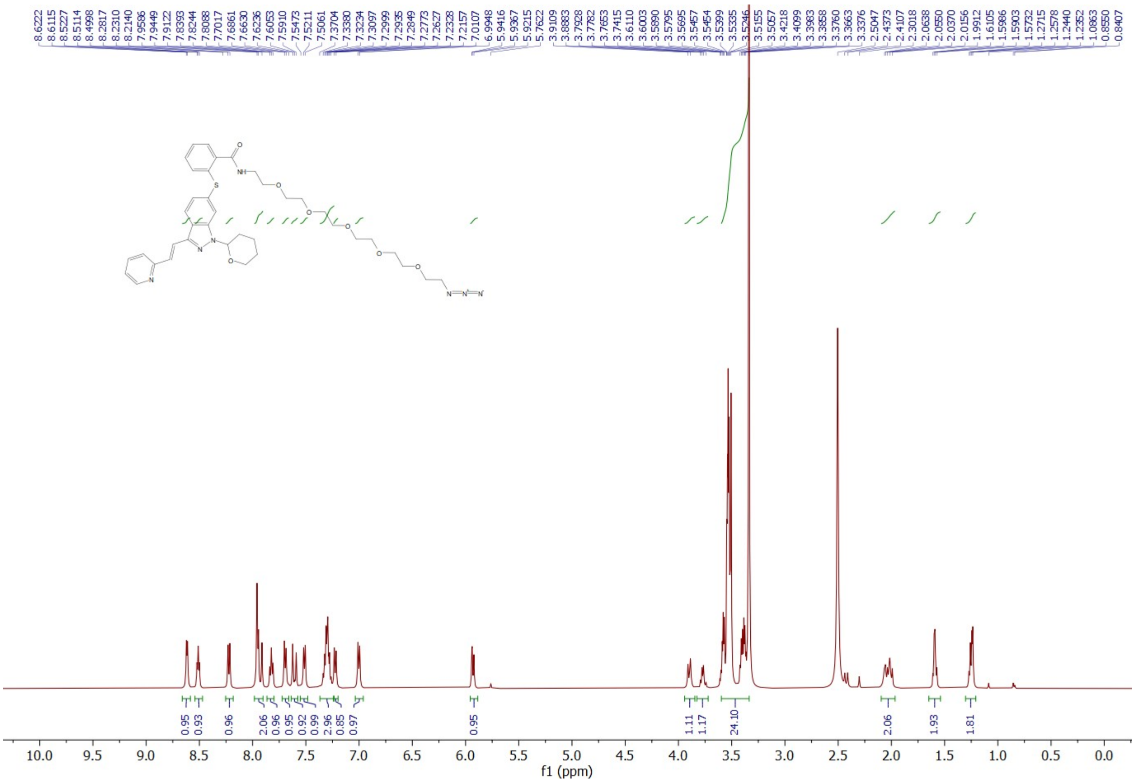

Figure S34. ^{1}H NMR (500 MHz, DMSO-d_6) spectrum of compound **18**.

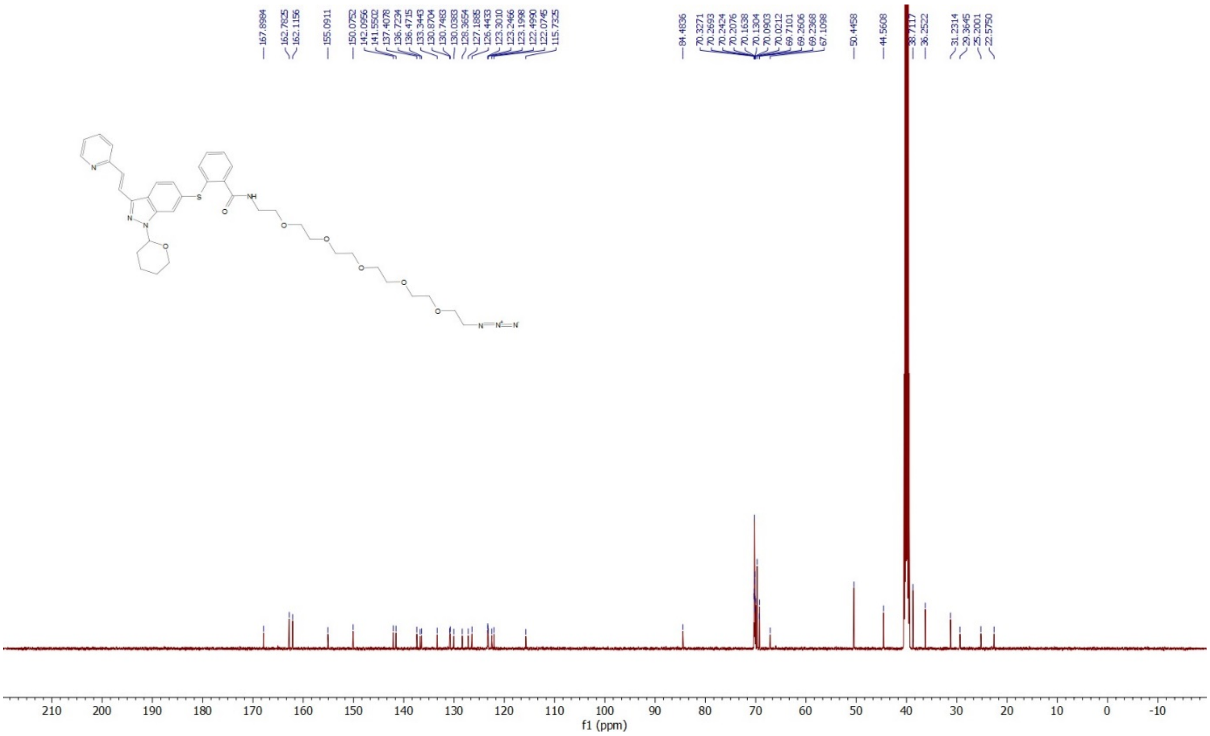

Figure S35. ^{13}C NMR (125 MHz, DMSO-d$_6$) spectrum of compound **18**.

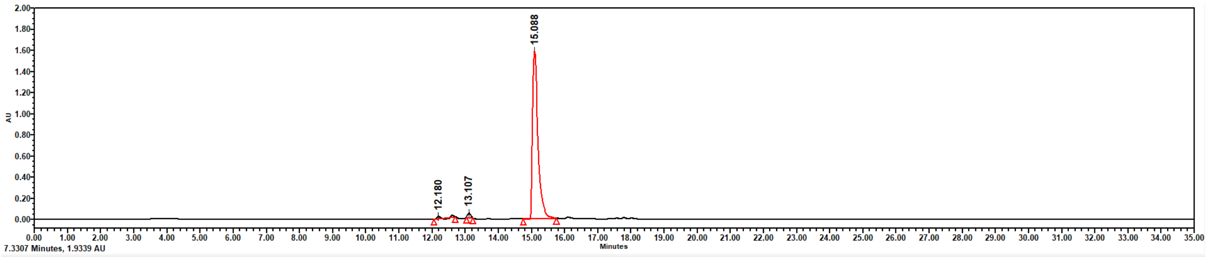

	Name	Retention Time (min)	Area (µV*sec)	% Area	Height (µV)	Int Type	Amount	Units	Peak Type	Peak Codes
1		12.180	367956	1.96	22906	bb			Unknown	I08
2		13.107	270097	1.44	42384	bb			Unknown	
3		15.088	18180035	96.61	1586144	bb			Unknown	

Figure S36. HPLC chromatogram of compound **18** showing >96% purity.

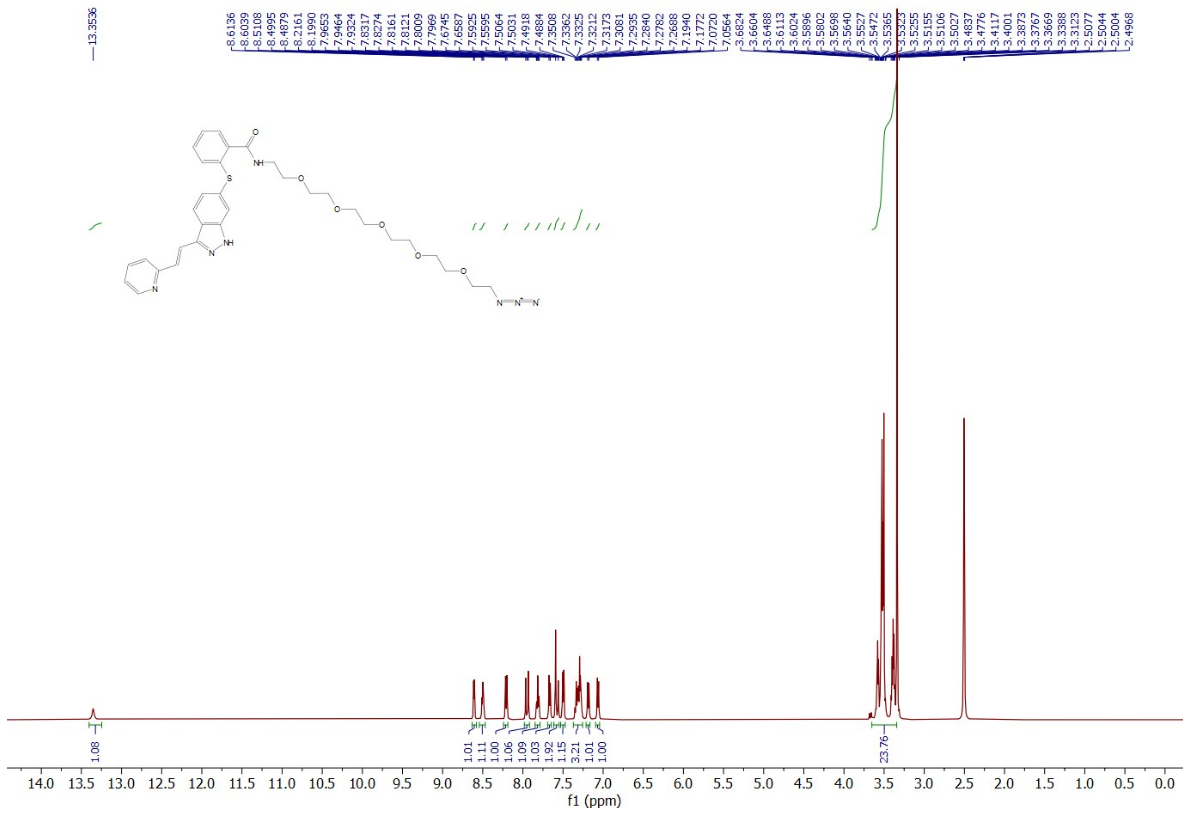

Figure S37. ^{1}H NMR (500 MHz, DMSO-d$_6$) spectrum of compound **19**.

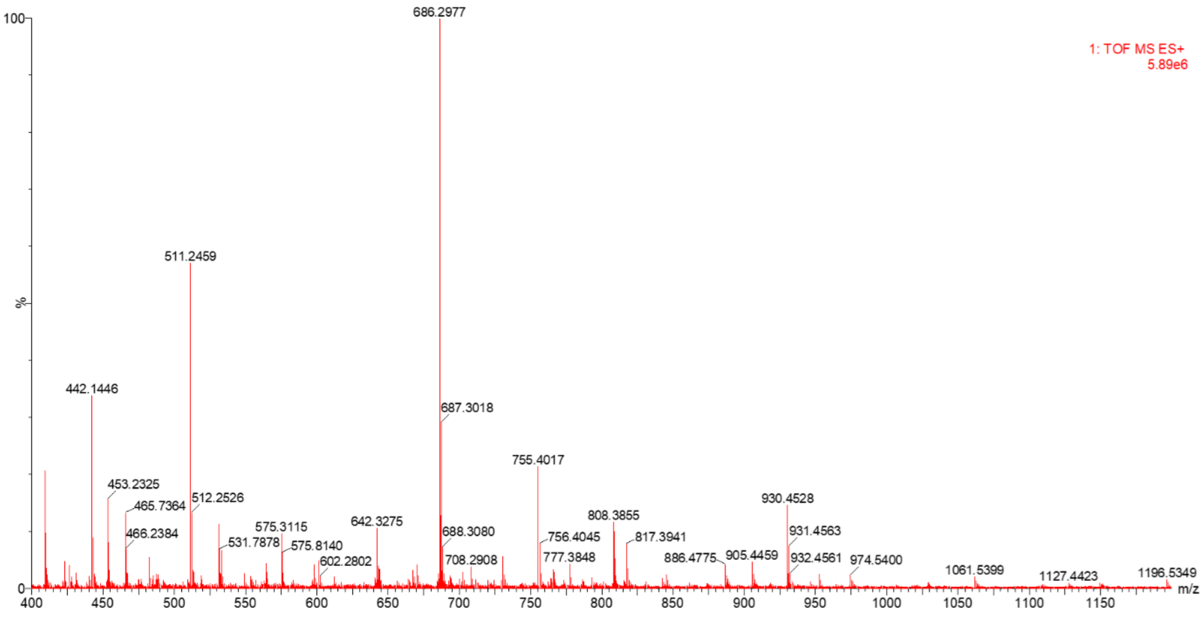

Figure S38. Mass spectra (ESI-MS) of compound **19**.

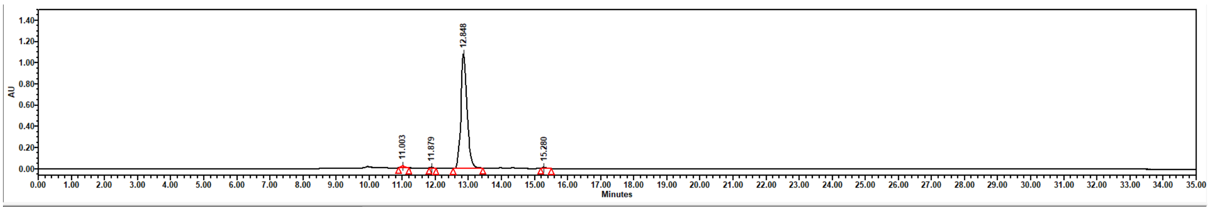

	Name	Retention Time (min)	Area (µV*sec)	% Area	Height (µV)	Int Type	Peak Type	Peak Codes
1		11.003	155921	1.10	18123	bb	Unknown	
2		11.879	40241	0.28	6180	bb	Unknown	I08
3		12.848	13924699	98.07	1078593	bb	Unknown	
4		15.280	78591	0.55	9250	bb	Unknown	I08

Figure S39. HPLC chromatogram of compound **19** displaying >98% purity.

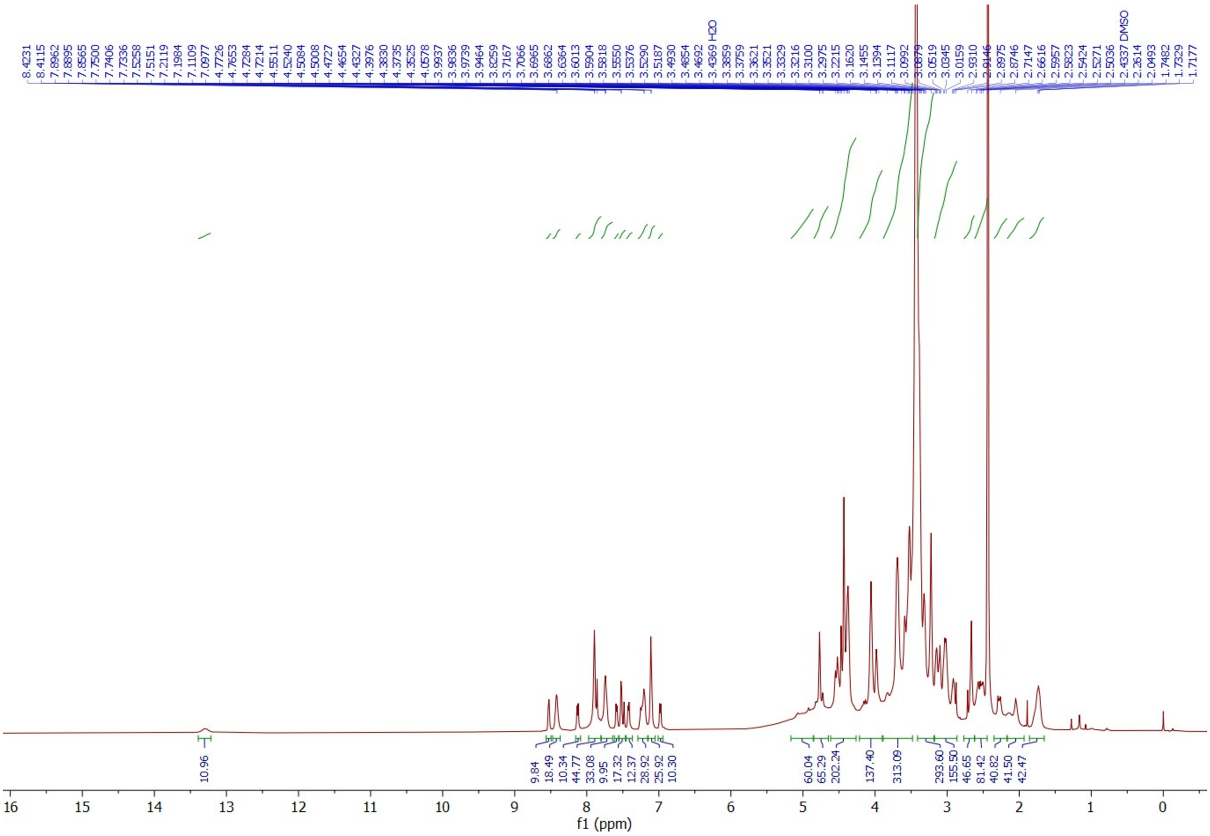

Figure S40. ^{1}H NMR (500 MHz, DMSO-d_6) spectrum of compound **20**.

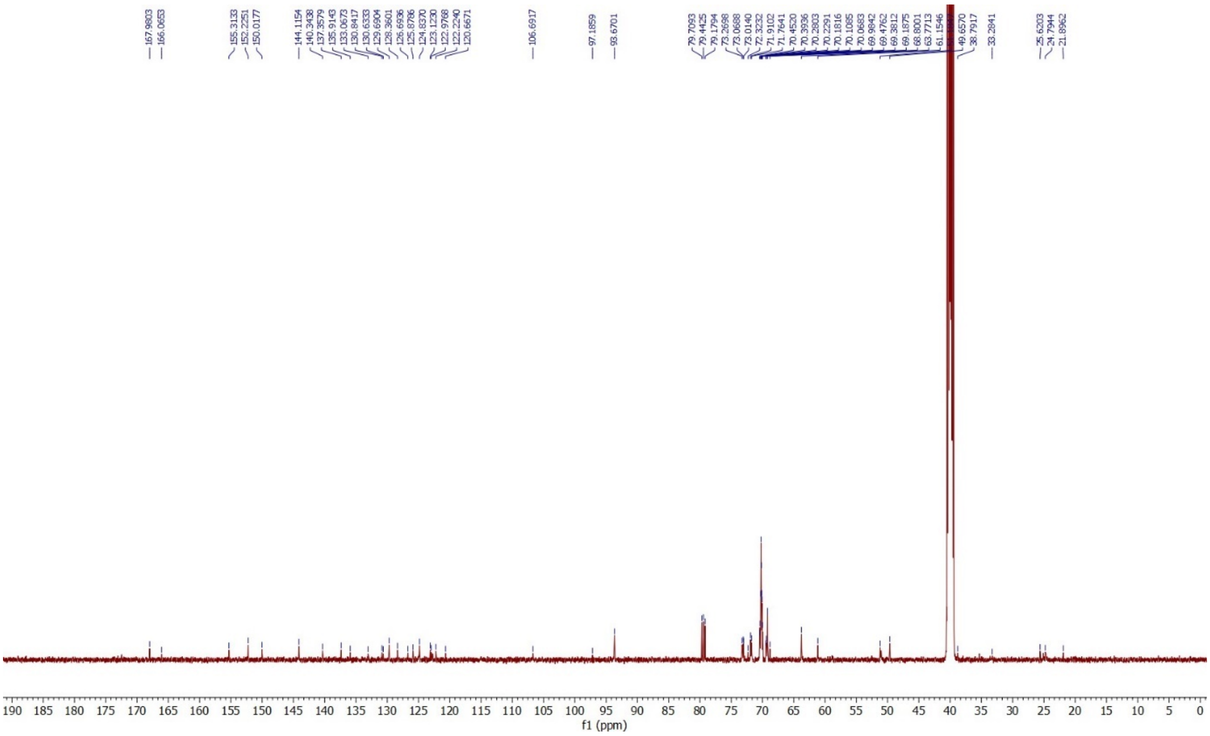

Figure S41. ^{13}C NMR (125 MHz, DMSO-d$_6$) spectrum of compound **20**.

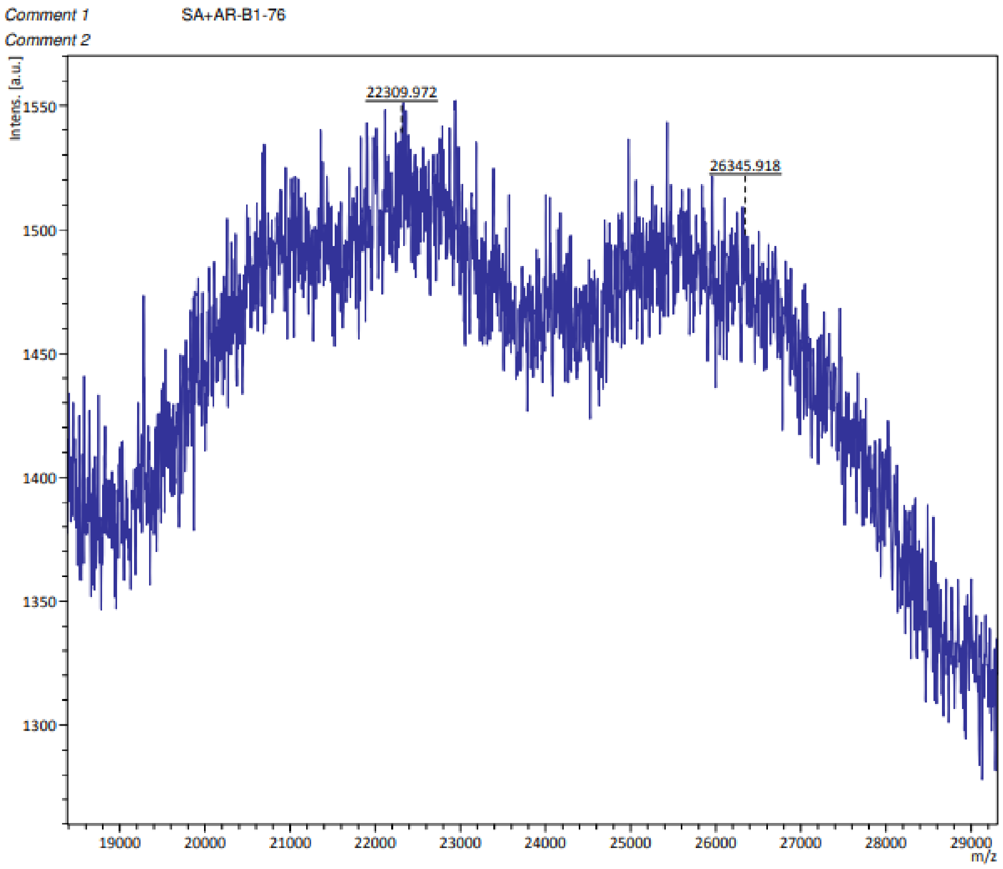

Figure S42. MALDI-TOF spectra dendrimer **20**.

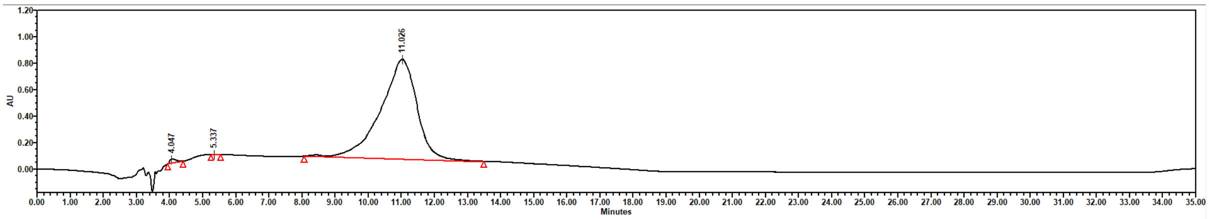

Name	Retention Time (min)	Area (µV*sec)	% Area	Height (µV)	Int Type	Peak Type
1	4.047	374421	0.65	28411	bb	Unknown
2	5.337	7106	0.01	845	bb	Unknown
3	11.026	57201622	99.34	754433	bb	Unknown

Figure S43. HPLC chromatogram confirming >99% purity of compound **20**.

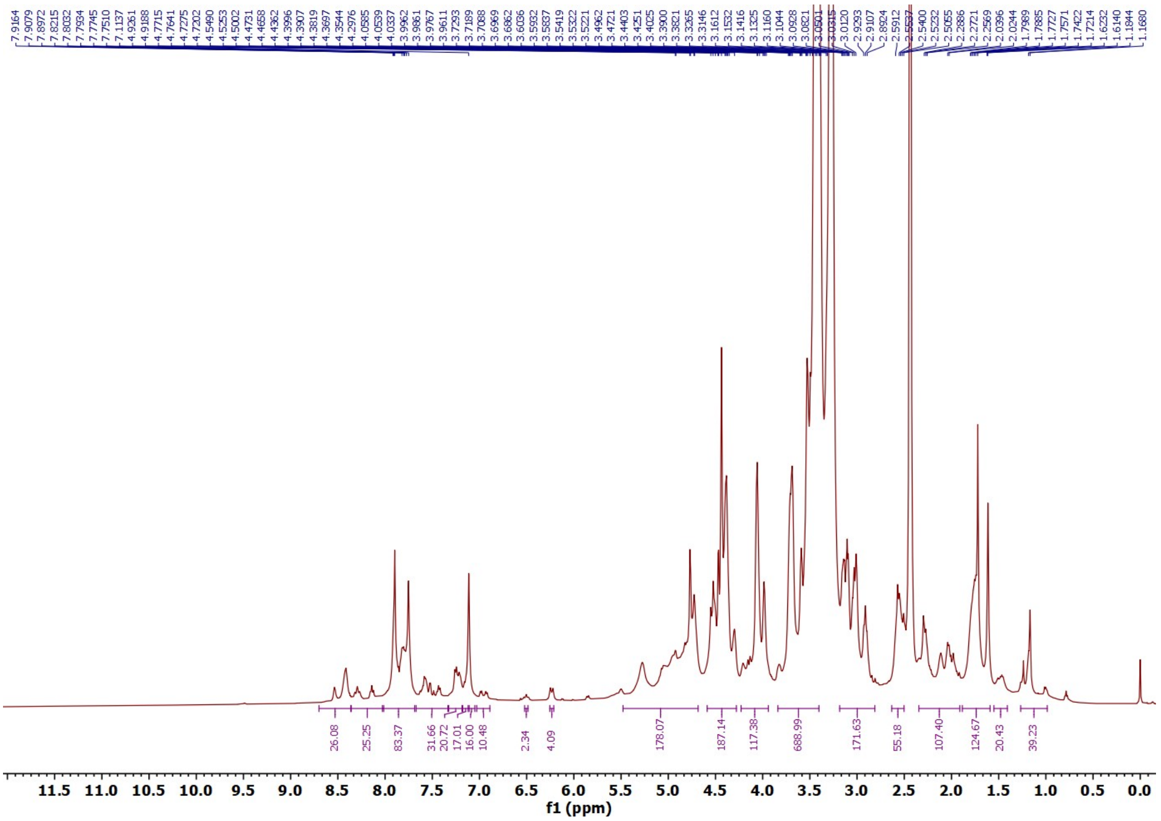

Figure S44. ^{1}H NMR (500 MHz, DMSO-d$_6$) spectrum of compound **21**.

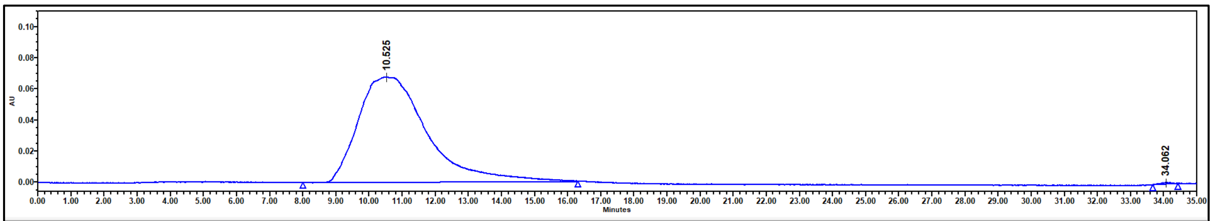

Name	Retention Time (min)	Area (µV*sec)	% Area	Height (µV)	Int Type	Amount	Units	Peak Type	Peak Codes
1	10.525	9740088	99.77	67608	bb			Unknown	
2	34.062	22524	0.23	960	bb			Unknown	

Figure S45. HPLC chromatogram verifying >99% purity of compound **21**.

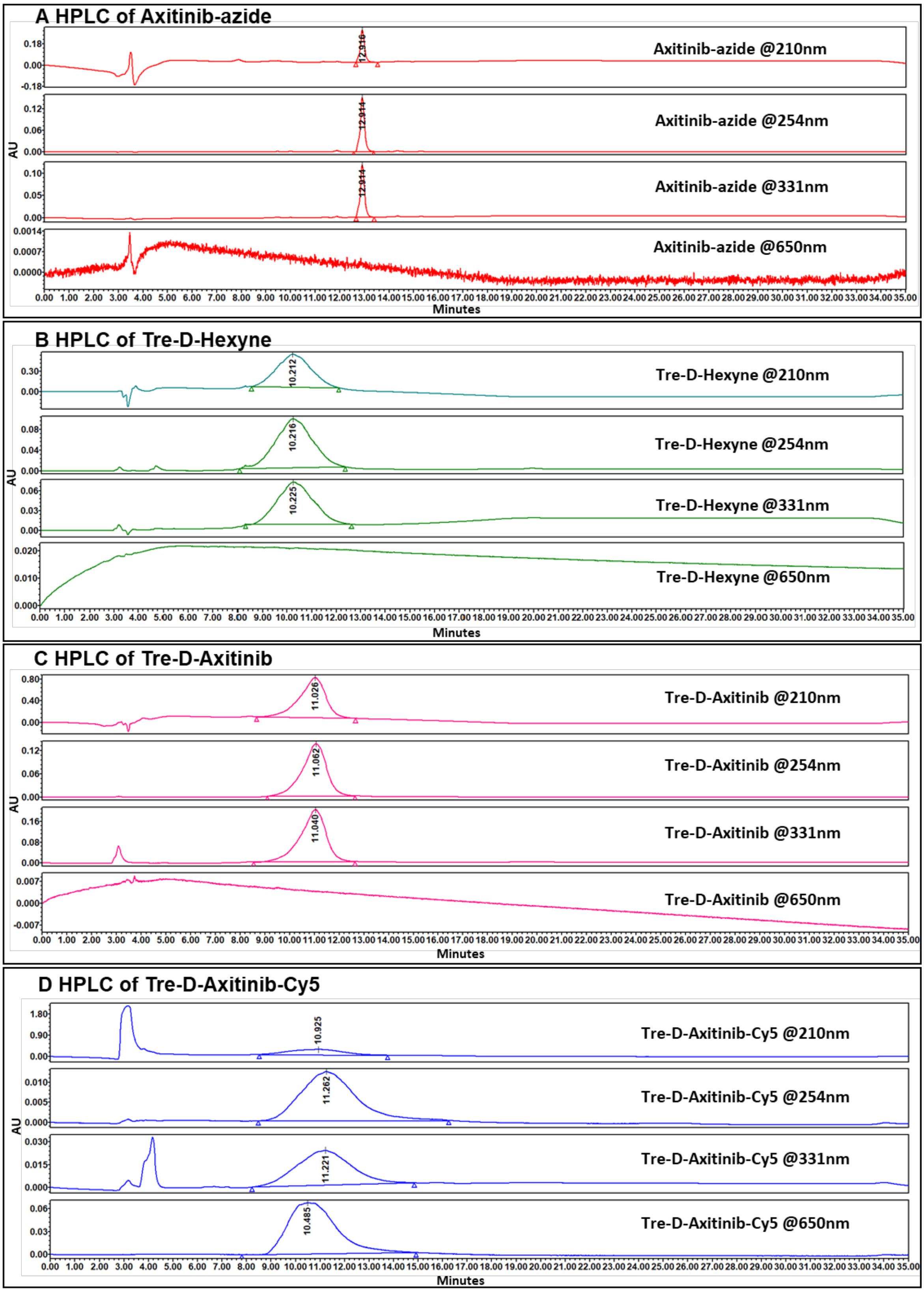

Figure S46: HPLC chromatogram of the intermediates and final dendrimer conjugates at multiple wavelengths [210 (dendrimer), 254 (aromatic), 31 (axitinib), and 650nm (Cy5).

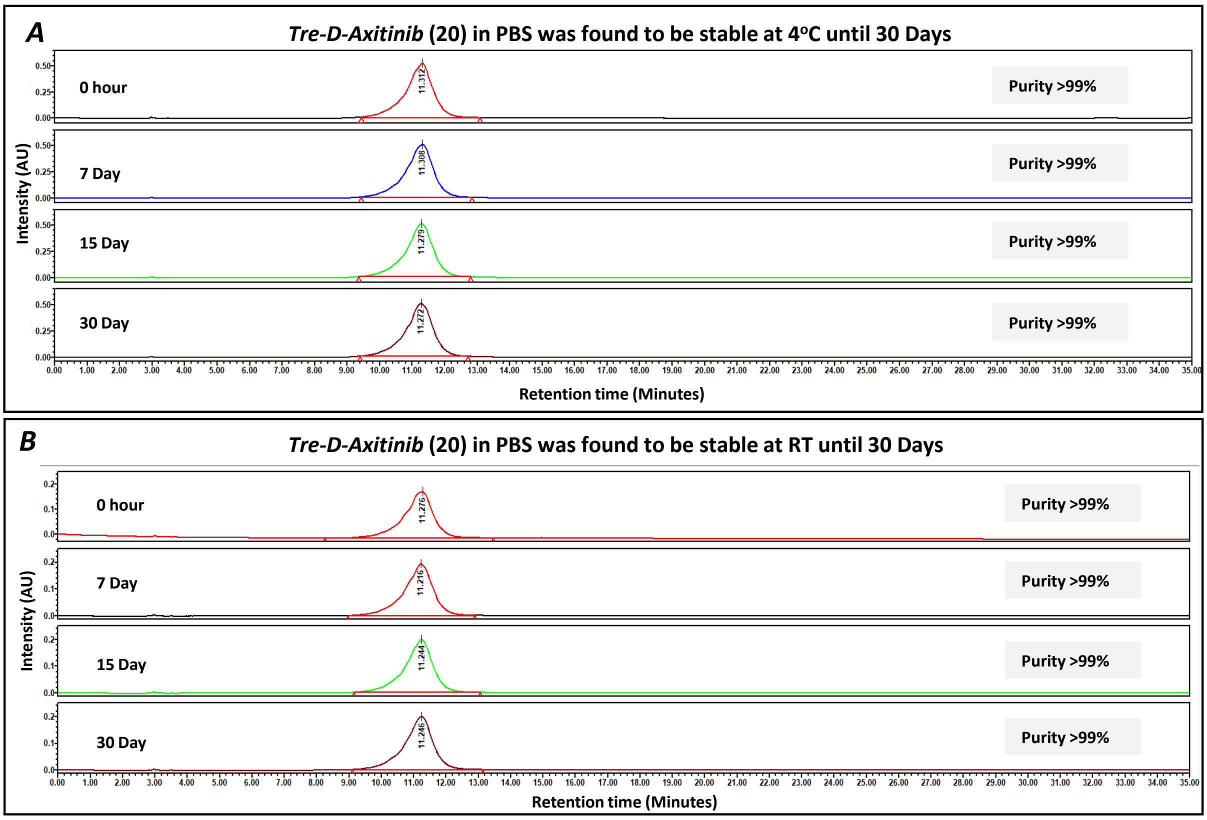

Figure S47: HPLC analysis (UV detection at 331 nm) showing that Tre-D-Axitinib (**20**) formulated in PBS remains stable at 4 °C (**A**) and RT (**B**) for up to 30 days.

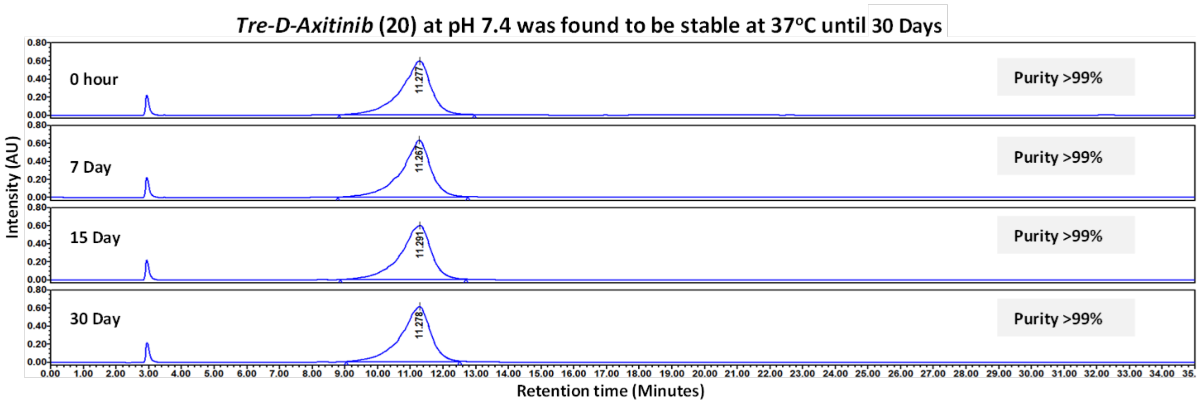

Figure S48: HPLC analysis (UV detection at 331 nm) showing that Tre-D-Axitinib (**20**) remains stable under physiological conditions (37 °C) for up to 30 days.

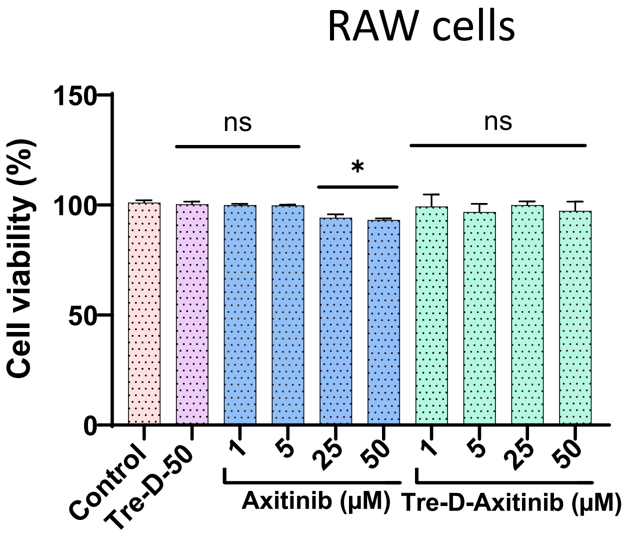

Figure S49: *In vitro* cellular compatibility evaluation of *Tre-D-Axitinib* in RAW macrophages.
